# Catalytic enantioselective radical coupling of activated ketones with *N*-aryl glycines†
†Electronic supplementary information (ESI) available. CCDC 1836991 and 1843336. For ESI and crystallographic data in CIF or other electronic format see DOI: 10.1039/c8sc02948b


**DOI:** 10.1039/c8sc02948b

**Published:** 2018-08-27

**Authors:** Yang Liu, Xiangyuan Liu, Jiangtao Li, Xiaowei Zhao, Baokun Qiao, Zhiyong Jiang

**Affiliations:** a Key Laboratory of Natural Medicine and Immuno-Engineering of Henan Province , Henan University , Kaifeng , Henan 475004 , P. R. China . Email: chmjzy@henu.edu.cn; b Henan Key Laboratory of Organic Functional Molecule and Drug Innovation , School of Chemistry and Chemical Engineering , Henan Normal University , Xinxiang , Henan 453007 , P. R. China

## Abstract

Asymmetric H-bonding catalysis as a viable strategy for enantioselective radical coupling of ketones is demonstrated. With a visible-light-mediated dual catalytic system involving a dicyanopyrazine-derived chromophore (DPZ) photosensitizer and a chiral phosphoric acid (CPA), *N*-aryl glycines with a variety of 1,2-diketones and isatins underwent a redox-neutral radical coupling process and furnished two series of valuable chiral 1,2-amino tertiary alcohols in high yields with good to excellent enantioselectivities (up to 97% ee). In this catalysis platform, the formation of neutral radical intermediates between ketyl and H-bonding catalyst CPA is responsible for presenting stereocontrolling factors. Its success in this work should provide inspiration for expansion to other readily accessible ketones to react with various radical species, thus leading to a productive approach to access chiral tertiary alcohol derivatives.

## Abstract 2

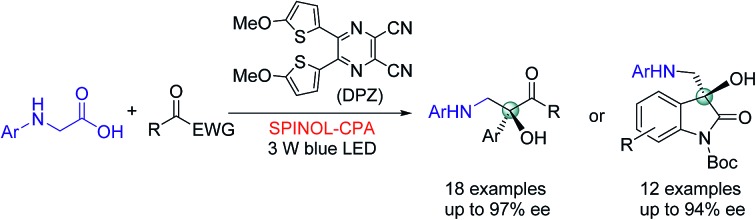
Asymmetric H-bonding catalysis as a viable strategy for enantioselective radical coupling of ketones is demonstrated.

## Introduction

The radical coupling reaction,^1^ wherein the nearly zero activation energy^2^ of connecting two distinct odd-electron partners enables a strong tendency to generate a new chemical bond, often furnishes an efficient and direct synthetic pathway to molecules with high functional group tolerance. Therefore, the development of compatible strategies to produce two types of radical species and facilitate the coupling reaction has attracted much attention from chemists over the last few decades.^1^–^3^ In 2011, the MacMillan group made a significant breakthrough^4^ in which such a transformation was realized *via* visible-light-driven photoredox catalysis.^5^ This convenient and sustainable platform promptly inspired a number of elegant efforts focusing on radical coupling.^6^ However, the enantioselective manifold still remains underdeveloped given that few examples have been established.^7^ This lack of development is mainly due to the high reactivity that results in an elusive precise absolute stereocontrol of the reaction. In 2015, Ooi and co-workers7*a* reported the first example with excellent enantioselectivity, wherein asymmetric α-coupling of *N*-arylaminomethanes with aldimines through cooperative catalysis^7^–^10^ of an Ir-centered photosensitizer and an ionic Brønsted acid led to chiral 1,2-diamine derivatives featuring a tertiary carbon stereocenter. Almost at the same time, the Meggers group described an asymmetric radical coupling of tertiary amines with 2-trifluoroacetyl imidazoles catalyzed by a chiral iridium complex.7*b* They also developed a cooperative Ru-centered photosensitizer and Rh-based chiral Lewis acid catalyst for the cross-coupling of 2-acyl imidazoles with α-silylamines.7*d* In their elegant work, α-aminoalkyl moieties were successfully introduced onto the more challenging quaternary carbon stereogenic center. Inarguably, the judicious use of imidazole as the substituent of ketones is crucial for the enantioselectivity, as its N atom was shown to enhance the ability of stereocontrol by interacting with the chiral Lewis acid. Although the groups of Xiao,6*f* Shah6*g* and Yang6*h* have demonstrated the viability of radical coupling of various readily accessible ketones with different substrates in a racemic manner, an enantioselective variety still constitutes a formidable challenge.

**Scheme 1:**
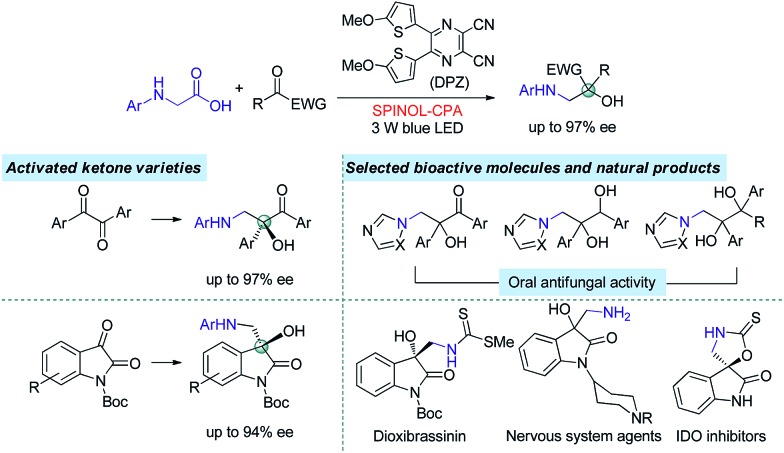
Outline of this work.

In the putative reaction mechanism of the radical-coupling transformations, ketones always experience a single-electron reduction to generate the corresponding ketyl variants.^6^,^7^ In 2013, the Knowles group9*f* revealed the strong basicity of ketyls and their capability of forming neutral ketyl radicals with a chiral Brønsted acid which could provide efficient enantioselective control for the coupling reaction with hydrazones. This prominent H-bonding catalytic strategy recently inspired us to accomplish a highly enantioselective photoreduction of 1,2-diketones.10*c* Accordingly, we speculated that such an approach would offer the possibility to address the desired radical coupling of undecorated ketones. If so, it should be a promising and general strategy as it would allow diverse radical species to connect with ketones, thus opening a fruitful avenue for the synthesis of the important chiral tertiary alcohols. Here, we report the development of a redox-neutral, enantioselective, radical coupling of *N*-aryl glycines with activated ketones, including acyclic 1,2-diketones and cyclic isatins (Scheme 1). The association of a dicyanopyrazine-derived chromophore (DPZ) as the photoredox catalyst and a 1,1′-spirobiindane-7,7′-diol (SPINOL)-based chiral phosphoric acid (CPA) as the H-bonding catalyst was demonstrated as being a workable catalytic system. Two series of chiral 1,2-amino tertiary alcohols that are significant structural scaffolds in synthetic and medicinal chemistry^11^ were prepared in high yields with good to excellent enantioselectivities.

## Results and discussion

**Scheme 2:**
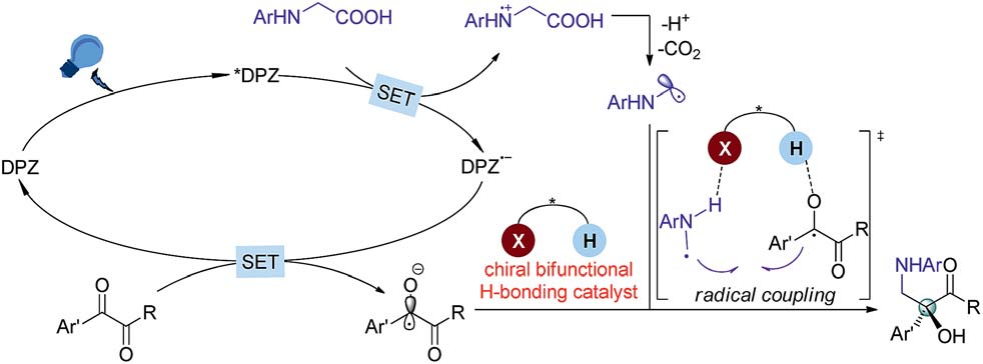
Design plan based on mechanistic considerations.

At the onset of our study, we sought to explore the coupling of ketyls derived from 1,2-diketones with α-amino radicals to provide the valuable enantioenriched 1,2-amino tertiary alcohols,11*a* for which no catalytic asymmetric synthesis has yet been developed. Our developed metal-free DPZ7g,^10^ was selected as the photosensitizer since 1,2-diketones (*e.g.* benzil, *E*red1/2 = –1.169 V and –1.251 V *vs.* a saturated calomel electrode (SCE) in CH_3_CN)10*c* could be directly reduced by DPZ˙^–^ (*E*red1/2 = –1.45 V *vs.* SCE in CH_2_Cl_2_) when the transformation experiences a reductive quenching cycle (Scheme 2). Furthermore, we intend to use *N*-aryl glycines (*e.g. N*-phenyl glycine, carboxylate ion, *E*_p_ = +0.52 [for the N moiety] and +1.09 V [for the COO^–^ moiety] *vs.* Ag/AgCl in CH_3_CN) as the precursors of α-amino radicals given the prominent ability to generate the radical species^12^*via* single-electron oxidative decarboxylation by the visible-light-activated DPZ, *i.e.* *DPZ (*E*^t^(S*/S˙^–^) = +0.91 V *vs.* in CH_2_Cl_2_).7g,10d,e More importantly, the formed α-amino radicals contain a H-bonding donor (N–H) which will be more nucleophilic *via* a possible interaction with the H-bonding acceptor (X = N, O, *etc*.) of a chiral bifunctional catalyst, thus facilitating coupling with the electrophilic α-ketone radicals.9s,^13^ Of note, the use of tertiary amines was susceptible to reduce 1,2-diketones to α-hydroxy ketones under photoredox catalysis.10c,^14^

To this end, we began the investigation with *N*-phenyl glycine **1a** and benzil **2a** in the presence of DPZ as the photoredox catalyst (Table 1).10c–e The initial investigation showed that the transformation furnished racemic product **3a** in 62% yield after 12 h when only using 0.5 mol% DPZ at 25 °C, indicating the feasibility of the reaction and the high reactivity (entry 1). Upon examining a range of chiral H-bonding catalysts and the reaction parameters (see Table S1 in the ESI†), we observed that the reaction conducted in CPME at 10 °C for 36 h in the presence of 1.5 mol% DPZ and 10 mol% chiral SPINOL-CPA^15^**C1** and with the combination of 30 mol% TBPB, 0.5 equiv. Na_2_S_2_O_4_ and 25 mg 5 Å MS as additives affords the desired chiral product **3a** in 78% yield with 93% ee (entry 2). Other SPINOL-CPAs (*e.g.*, **C2** and **C3**) with distinct substituents at the 6,6′-positions presented **3a** with lower ee values (entries 3–4). [Ru(bpy)_3_]Cl_2_ and Rose Bengal as plausible photoredox catalysts were evaluated (entries 5–6), but both the yield and enantioselectivity were decreased. Each of the three additives, which could regulate the strength of H-bonding interaction by exploiting the salt effect (*i.e.* TBPB10*d* and Na_2_S_2_O_4_) or diminishing the moisture of the reaction system (*i.e.* molecular sieves7*g*), was found to slightly affect the enantioselectivity to the same degree (entries 7–9). The results reveal that all of the additives jointly exerted a positive influence on the stereocontrol of **C1**. The control experiments confirmed that DPZ, visible light, and an oxygen-free environment are indispensable for the transformation to occur (entries 10–12). Note that the reaction performed under the standard conditions but in the absence of catalyst **C1** still produced *rac*-**3a** in 53% yield (entry 13), suggesting the existence of a considerable competitive achiral background reaction.

**Table 1:** Optimization of the reaction conditions^*a*^

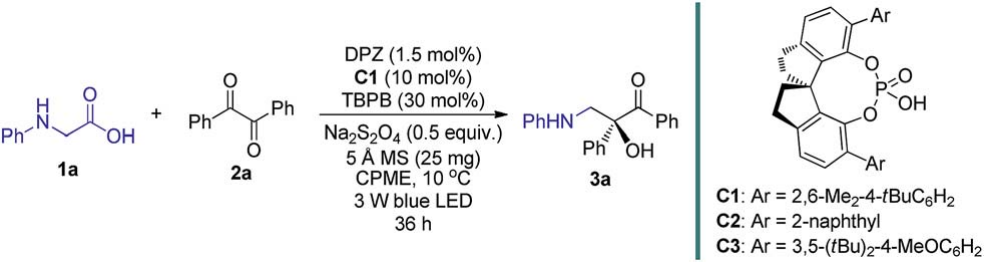			
Entry	Variation from standard conditions	Yield^*b*^ (%)	ee^*c*^ (%)
1	0.5 mol% DPZ in CH_2_Cl_2_ at 25 °C and without **C1** and any additives, 12 h	62	N.A.
2	None	78	93
3	**C2** instead of **C1**	74	76
4	**C3** instead of **C1**	73	67
5	Ru(bpy)_3_Cl_2_·6H_2_O instead of DPZ	58	89
6	Rose Bengal instead of DPZ	69	91
7	No TBPB	76	89
8	No Na_2_S_2_O_4_	75	90
9	No 5 Å MS	76	90
10	No DPZ	35	88
11	No light	0^*d*^	N.A.
12	Under air	0^*e*^	N.A.
13	No **C1**	53	N.A.

^*a*^Reaction conditions: **1a** (0.075 mmol), **2a** (0.05 mmol), degassed CPME (1.0 mL), 10 °C, irradiation with blue LED (3 W, 450 nm), 36 h.

^*b*^Yield of the isolated product.

^*c*^Determined by HPLC analysis on a chiral stationary phase.

^*d*^No reaction was detected.

^*e*^ **1a** was consumed, but no **3a** was obtained. TBPB = tetra-*n*-butylphosphonium bromide. CPME = cyclopentyl methyl ether. N.A. = not available.

**Scheme 3:**
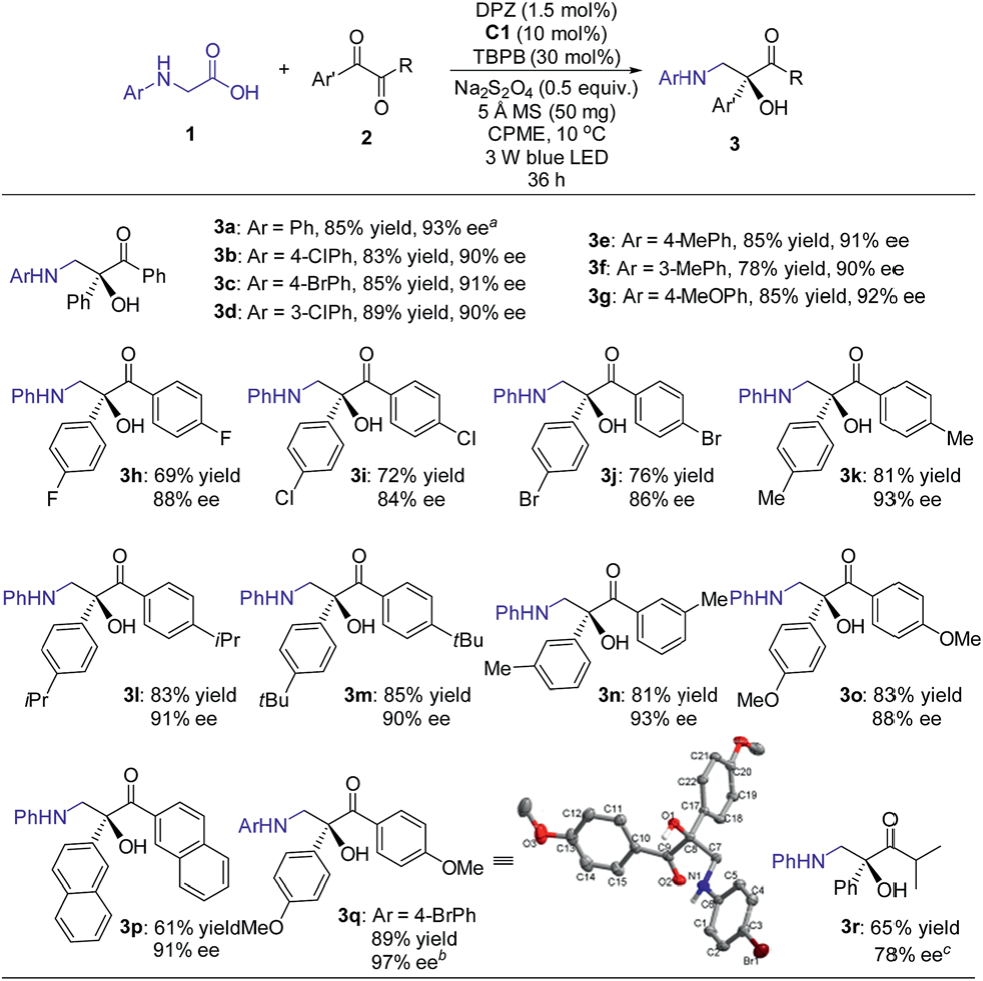
Reactions of *N*-aryl glycines with 1,2-diketones. Reaction conditions: **1** (0.15 mmol), **2** (0.1 mmol), DPZ (1.5 mol%), **C1** (10 mol%), TBPB (30 mol%), Na_2_S_2_O_4_ (0.5 equiv.), 5 Å MS (50 mg), degassed CPME (2.0 mL), 10 °C, irradiation with a blue LED (3 W, 450 nm), 36 h. The yield amount was isolated by flash column chromatography on a silica gel. ee was determined by HPLC analysis on a chiral stationary phase. ^*a*^On a 1.0 mmol scale, 48 h, yield of **3a** = 87%, ee of **3a** = 93%. ^*b*^The ee value was obtained after a single recrystallization. Initial data: 85% ee. ^*c*^Reaction conditions: **1** (0.15 mmol), **2** (0.1 mmol), DPZ (1.5 mol%), **C2** (10 mol%), 4 Å MS (50 mg), degassed CPME (2.0 mL), –5 °C, 36 h. Under the previous reaction conditions, ee = 34%.

With the optimal reaction conditions in hand, the scope of this H-bonding catalysis-enabled asymmetric radical coupling strategy was examined (Scheme 3). The reactions of **2a** with a variety of *N*-aryl glycines **1** furnished adducts **3a–g** in 78 to 89% yields with 90 to 93% ees within 36 h. Electron-deficient or electron-donating substituents at the *para*- and *meta*-positions of the aryl ring presented similar reactivities and enantioselectivities. An attempt at performing the reaction of **1a** with **2a** in a 1.0 mmol scale presented a similar reactivity and enantioselectivity as **3a**, with 87% yield with 93% ee achieved after 48 h (footnote *a*). With respect to symmetric 1,2-diketones, the reaction tolerated a wide range of aryl substituents regardless of their electronic properties and substitution patterns, and corresponding products **3h–q** were obtained in 61 to 89% yields with 84 to 97% ees. Based on the persistent radical effect,^13^ the slightly lower enantioselectivity for 1,2-diketones (**3h–j**) with electron-withdrawing substituents is likely due to the stronger racemic background reaction, as the higher stability of these ketyl intermediates would facilitate a coupling with the unstable α-amino radical. For 3-methyl-1-phenyl-1,2-butanedione as a representative of unsymmetrical 1,2-diketones, product **3r** was obtained in only 34% ee. However, the modified reaction conditions, that are **C2** as the chiral catalyst and 4 Å MS as an additive in CPME at –5 °C, could furnish **3r** in 78% ee. The stereochemistry of these adducts was assigned based on the structure of **3q**, as solved by single crystal X-ray diffraction.^16^

**Scheme 4:**
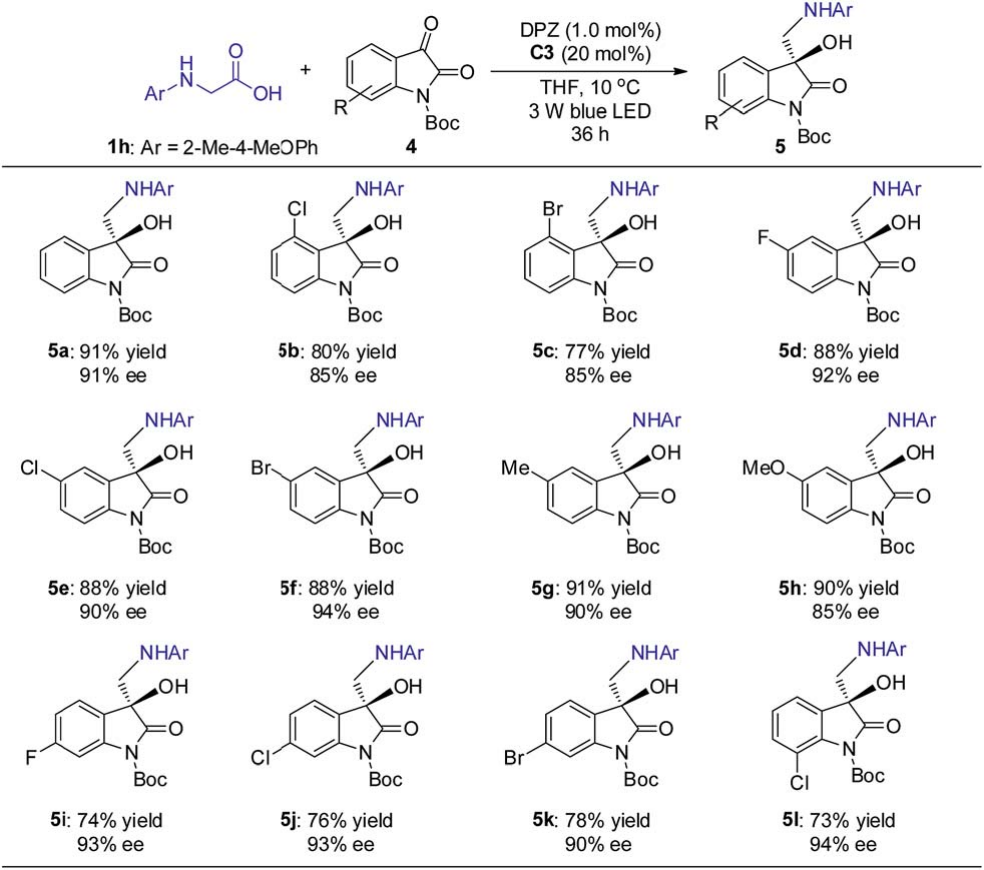
Reactions of *N*-aryl glycine with isatins. ^+^Reaction conditions: **1h** (0.15 mmol), **4** (0.1 mmol), DPZ (1.0 mol%), **C1** (20 mol%), degassed THF (2.0 mL), 10 °C, irradiation with a blue LED (3 W, 450 nm), 36 h. The yield amount was isolated by flash column chromatography on silica gel. ee was determined by HPLC analysis on a chiral stationary phase.

The promising results inspired us to further evaluate this catalytic strategy for isatins, a representative cyclic ketone, to first construct the important chiral 3-hydroxy-3-aminoalkylindolin-2-one derivatives in a direct manner.11b–g As depicted in Scheme 4, under the same catalysis platform but with modified reaction conditions (1.0 mol% DPZ, 20 mol% SPINOL-CPA **C3** in THF at 10 °C), the transformations of *N*-aryl glycine **1h** with *N*-Boc-substituted isatins **4** were complete within 36 h, providing the desired adducts **5a–l** with diverse substituents on the aromatic ring of the isatins in 73 to 91% yields with 85 to 94% ees. It was observed that the substituent group at the 4-position (*e.g.*, **5b–c**) presented a slightly decreased enantioselectivity, probably owing to the steric hindrance.

**Scheme 5:**
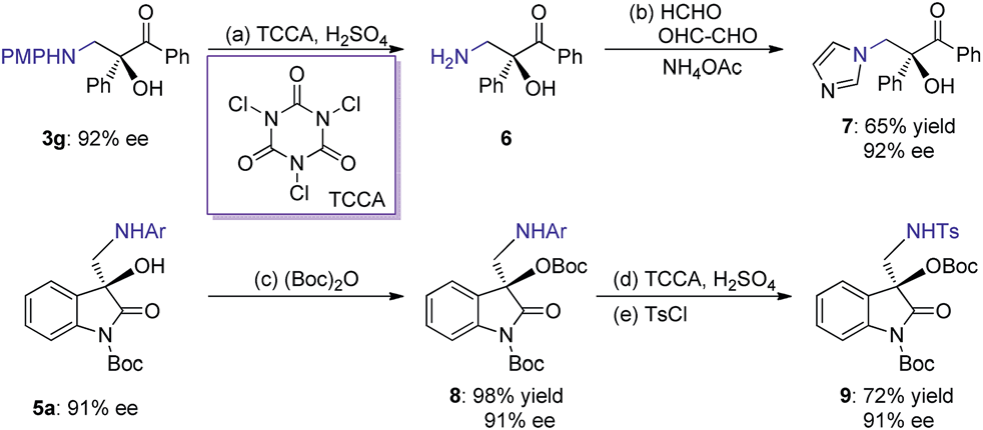
Synthetic applications. (a) TCCA (0.5 equiv.), H_2_SO_4_ (1 M, aq., 2.0 equiv.), CH_3_CN/H_2_O = 1 : 1, 16 h. (b) HCHO (2.0 equiv.), OHC–CHO (2.0 equiv.), NH_4_OAc (2.0 equiv.), MeOH, 80 °C, 5 h, 65% yield, 92% ee. (c) (Boc)_2_O (1.1 equiv.), DMAP (0.2 equiv.), DCM, 0 °C, 0.5 h, 98% yield, 91% ee. (d) TCCA (0.5 equiv.), H_2_SO_4_ (1 M, aq., 2.0 equiv.), CH_3_CN/H_2_O = 1 : 1, 16 h. (e) TsCl (2.0 equiv.), Et_3_N (2.0 equiv.), EtOAc, 0 °C to r.t., 5 h, 72% yield in two steps, 91% ee. PMP = *para*-methoxyphenyl; TCCA = *N*,*N*′,*N*′′-trichloroisocyanuric acid; (Boc)_2_O = di-*tert*-butyl dicarbonate; Boc = *tert*-butyl carbonate; DMAP = 4-dimethylaminopyridine; TsCl = *p*-toluenesulfonyl chloride; Ts = tosyl.

The two series of enantiomerically enriched 1,2-amino-alcohols featuring oxo-hetero-quaternary carbon stereocenters are direct precursors to many bioactive natural and non-natural compounds (Scheme 1). For example, the treatment of adduct **3g** derived from 1,2-diketone using TCCA readily cleaved the PMP *N*-protective group (Scheme 5). The resultant amine **6** was then transformed into chiral 1,2-imidazolyl tertiary alcohol **7** that possesses oral antifungal activity11*a* in 65% yield over two steps with 92% ee. The transformation of adducts from isatins was also carried out. The Boc-protected product **8** was rapidly obtained in 98% yield by treating **5a** with (Boc)_2_O and DMAP. The replacement of 2-methyl-4-methoxyphenyl with Ts as the *N*-protective group was performed through a sequential process involving the use of TCCA for the deprotection and TsCl for the protection, furnishing product **9** in 72% yield and without diminishing the ee. The results clearly indicate that chiral products **5** are excellent synthetic intermediates for conveniently synthesizing the biologically important 3-hydroxy-3-aminoalkylindolin-2-one variants as shown in Scheme 1.11b–g

**Scheme 6:**
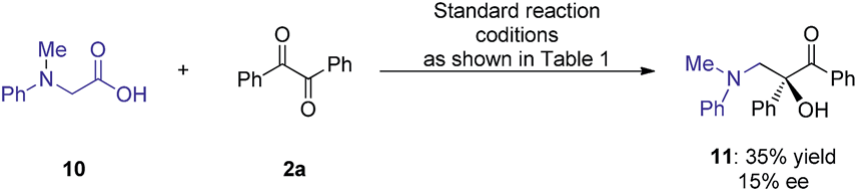
Transformation of **10** and **2a**.

Based on the previous examples^10^,^12^ and the product structure, the reaction between *N*-aryl glycines and ketones that underwent a single electron transfer (SET) redox radical coupling process to form the products is reasonable. The Stern–Volmer experiments^17^ confirmed that the catalytic cycle was triggered from the reductive quenching of *DPZ (Scheme 2). To better understand the role of the chiral CPA in asymmetric induction, the transformation of *N*-phenyl-*N*-methyl glycine **10** with benzil **2a** under the standard reaction conditions as shown in Table 1 was carried out, and adduct **11** was obtained in 35% yield with 15% ee (Scheme 6). The lower yield than that achieved with the transformation of *N*-phenyl glycine **1a** (entry 2, Table 1) was due to the deteriorated chemoselectivity, as the reduced product of **2a**, *i.e.*, benzoin, was obtained in a considerable amount. Note that in the absence of catalyst **C1**, **3a** was also produced in a decreased yield in the reaction of **1a** with **2a** (entry 13, Table 1). According to the persistent radical effect,^13^ the results suggest the existence of an interaction between N–H of **1a** as a H-bonding donor and P=O of the chiral CPA as a H-bonding acceptor, thus increasing the nucleophilicity of the α-amino radicals. In this context, the chiral CPA should serve as a bifunctional catalyst to activate the reaction and provide stereocontrol for the new C–C bond formation,9*s* for which a ternary transition state as shown in Scheme 2 is plausible.

## Conclusions

In summary, we developed an enantioselective radical coupling of *N*-aryl glycines with diverse activated ketones, including acyclic 1,2-diketones and cyclic isatins, *via* visible-light-driven cooperative photoredox and chiral H-bonding catalysis. A range of significant enantioenriched 1,2-amino alcohols that feature an oxo-hetero-quaternary carbon stereocenter were obtained in high yields and ees. This work robustly demonstrates the viability of H-bonding catalysis for the highly reactive radical-coupling of ketones by directly providing a stereocontrolled environment for ketones. We believe that this dual catalytic system can serve as a powerful tool to address a variety of radical coupling reactions of diverse ketones through flexibly selecting a photoredox catalyst and a H-bonding catalyst, thus providing a direct and productive approach to access various chiral tertiary alcohols. We also anticipate that this work will inspire the pursuit of novel enantioselective radical coupling for other oxidative substrates with feasible H-bonding acceptor moieties.

## Conflicts of interest

There are no conflicts to declare.

## Supplementary Material

Supplementary information

Crystal structure data
