# Supplementary material for: Catalytic enantioselective radical coupling of activated ketones with N-aryl glycines
Source: Chem Sci. 2018 Aug 27;9(42):8094–8. doi: 10.1039/c8sc02948b (PMC6238713; doi:10.1039/c8sc02948b)
Supplement: Supplementary file 1 [file SC-009-C8SC02948B-s001.pdf]

Electronic Supplementary Material (ESI) for Chemical Science.
This journal is © The Royal Society of Chemistry 2018

Supporting Information

Catalytic Enantioselective Radical Coupling of Activated Ketones with *N*-Aryl Glycines

Yang Liu,[a] Xiangyuan Liu,[a] Jiangtao Li,[a] Xiaowei Zhao,[a] Baokun Qiao,[a] and Zhiyong Jiang[a,b,]*

[a]Key Laboratory of Natural Medicine and Immuno-Engineering of Henan Province, Henan University, Jinming Campus, Kaifeng, Henan, 475004, P. R. China

[b]Henan Key Laboratory of Organic Functional Molecule and Drug Innovation, School of Chemistry and Chemical Engineering, Henan Normal University, Xinxiang, Henan, P. R. China, 453007

E-mail: chmjzy@henu.edu.cn

Table of Contents

1. General information S3-S4
2. Optimization of reaction conditions S5-S7
3. General experimental procedures S8-S9
4. Mechanism studies S10
5. Procedures of synthetic applications S11-S12
6. Determination of the absolute configurations S13-14
7. Characterization of adducts S15-S48
8. Copies of NMR spectra S49-S82

1. General information

General procedures and methods

Experiments involving moisture and/or air sensitive components were performed under a positive pressure of argon in oven-dried glassware equipped with a rubber septum inlet. Dried solvents and liquid reagents were transferred by oven-dried syringes or hypodermic syringe cooled to ambient temperature in a desiccator. Reactions mixtures were stirred in 10 mL sample vial with Teflon-coated magnetic stirring bars unless otherwise stated. Moisture in non-volatile reagents/compounds was removed in high *vacuo* by means of an oil pump and subsequent purging with nitrogen. Solvents were removed *in vacuo* under ~30 mmHg and heated with a water bath at 30–35 ^{o}C using rotary evaporator with aspirator. The condenser was cooled with running water at 0 ^{o}C.

All experiments were monitored by analytical thin layer chromatography (TLC). TLC was performed on pre-coated plates, 60 F_{254}. After elution, plate was visualized under UV illumination at 254 nm for UV active material. Further visualization was achieved by staining $Ce(SO_4)_2$ and anisaldehyde solution. For those using the aqueous stains, the TLC plates were heated on a hot plate.

Columns for flash chromatography (FC) contained *silica gel* 200–300 mesh. Columns were packed as slurry of *silica gel* in petroleum ether and equilibrated solution using the appropriate solvent system. The elution was assisted by applying pressure of about 2 atm with an air pump.

Instrumentations

Proton nuclear magnetic resonance (^{1}H NMR) and carbon NMR (^{13}C NMR) were recorded in $CDCl_3$ otherwise stated. Chemical shifts are reported in parts per million (ppm), using the residual solvent signal as an internal standard: $CDCl_3$ (^{1}H NMR: δ 7.26, singlet; ^{13}C NMR: δ 77.16, triplet). Multiplicities were given as: *s* (singlet), *d* (doublet), *t* (triplet), *q* (quartet), *quintet*, *m* (multiplets), *dd* (doublet of doublets), *dt* (doublet of triplets), and *br* (broad). Coupling constants (*J*) were recorded in Hertz (Hz). The number of proton atoms (*n*) for a given resonance was indicated by *n*H. The number of carbon atoms (*n*) for a given resonance was indicated by *n*C. HRMS (Analyzer: TOF) was reported in units of mass of charge ratio (m/z). Mass samples were dissolved in CH_3CN (HPLC Grade) unless otherwise stated.

Optical rotations were recorded on a polarimeter with a sodium lamp of wavelength 589 nm and reported as follows; $[\alpha]_{\lambda}^{T\,°C}$ (c = g/100 mL, solvent). Melting points were determined on a melting point apparatus.

Enantiomeric excesses were determined by chiral High Performance Liquid Chromatography (HPLC) analysis. UV detection was monitored at 254 nm and 210 nm at the same time. HPLC samples were dissolved in HPLC grade isopropanol (IPA) unless otherwise stated.

Materials

All commercial reagents were purchased with the highest purity grade. They were used without further purification unless specified. All solvents used, mainly petroleum ether (PE) and ethyl acetate (EtOAc) were distilled. Anhydrous DCM, CH_3CN were freshly distilled from CaH_2 and stored under N_2 atmosphere. THF, Et_2O, MTBE, 1,2-dimethoxyethane, CPME, and toluene were freshly distilled from sodium/benzophenone before use. All compounds synthesized were stored in a −20 °C freezer and light-sensitive compounds were protected with aluminium foil.

2. Optimization of reaction conditions

Table S1. Optimization of Reaction Conditions.[a]

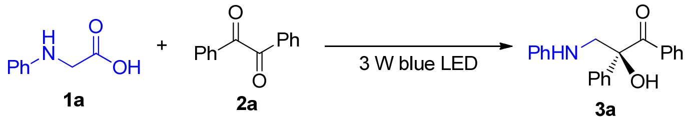

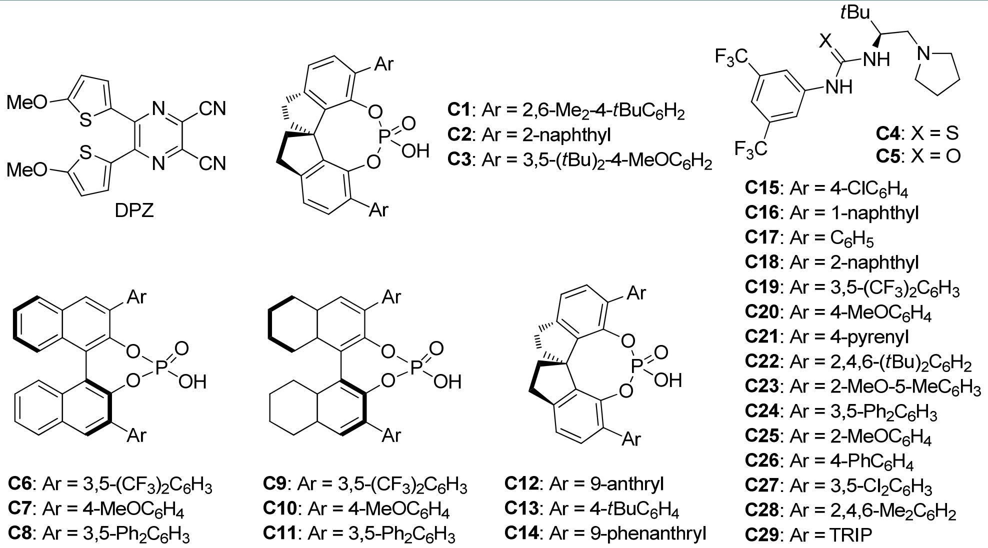

Entry	PC (mol%)	Cat.	Solvent	*T* (°C)	Additive (equiv/mg)	ee (%)[b]
1	DPZ [1.0]	--	CH_2Cl_2	25	--	--[c]
2	DPZ [1.0]	**C4**	CH_2Cl_2	25	--	0
3	DPZ [1.0]	**C5**	CH_2Cl_2	25	--	0
4	DPZ [1.0]	**C6**	CH_2Cl_2	25	--	7
5	DPZ [1.0]	**C7**	CH_2Cl_2	25	--	12
6	DPZ [1.0]	**C8**	CH_2Cl_2	25	--	34
7	DPZ [1.0]	**C9**	CH_2Cl_2	25	--	18
8	DPZ [1.0]	**C10**	CH_2Cl_2	25	--	16
9	DPZ [1.0]	**C11**	CH_2Cl_2	25	--	23
10	DPZ [1.0]	**C12**	CH_2Cl_2	25	--	36
11	DPZ [1.0]	**C13**	CH_2Cl_2	25	--	2
12	DPZ [1.0]	**C14**	CH_2Cl_2	25	--	45
13	DPZ [1.0]	**C15**	CH_2Cl_2	25	--	11
14	DPZ [1.0]	**C16**	CH_2Cl_2	25	--	18
15	DPZ [1.0]	**C17**	CH_2Cl_2	25	--	15
16	DPZ [1.0]	**C18**	CH_2Cl_2	25	--	15
17	DPZ [1.0]	**C19**	CH_2Cl_2	25	--	32
18	DPZ [1.0]	**C20**	CH_2Cl_2	25	--	11
19	DPZ [1.0]	**C21**	CH_2Cl_2	25	--	24
20	DPZ [1.0]	**C14**	Et_2O	25	--	41
21	DPZ [1.0]	**C14**	Tol.	25	--	40
22	DPZ [1.0]	**C14**	CH_3CN	25	--	61
23	DPZ [1.0]	**C22**	CH_3CN	25	--	65
24	DPZ [1.0]	**C23**	CH_3CN	25	--	41

25	DPZ [1.0]	**C24**	CH_3CN	25	--	51
26	DPZ [1.0]	**C25**	CH_3CN	25	--	41
27	DPZ [1.0]	**C26**	CH_3CN	25	--	27
28	DPZ [1.0]	**C27**	CH_3CN	25	--	33
29	DPZ [1.0]	**C28**	CH_3CN	25	--	62
30	DPZ [1.0]	**C28**	THF	25	--	67
31	DPZ [1.0]	**C28**	DCM	25	--	57
32	DPZ [1.0]	**C28**	Tol.	25	--	44
33	DPZ [1.0]	**C28**	Et_2O	25	--	66
34	DPZ [1.0]	**C28**	CPME	25	--	69
35	DPZ [1.0]	**C28**	MTBE	25	--	70
36	DPZ [1.0]	**C28**	MTBE	10	--	74
37	DPZ [1.0]	**C28**	MTBE	10	5Å MS (25 mg)	80
38	DPZ [1.0]	**C28**	MTBE	10	4Å MS (25 mg)	78
39	DPZ [1.0]	**C28**	MTBE	10	3Å MS (25 mg)	68
40	DPZ [1.0]	**C28**	MTBE	10	5Å MS (35 mg)	75
41	DPZ [1.0]	**C28**	MTBE	10	5Å MS (25 mg) + K_3PO_4 (0.2 equiv)	23
42	DPZ [1.0]	**C28**	MTBE	10	5Å MS (25 mg) + K_2HPO_4 (0.2 equiv)	80
43	DPZ [1.0]	**C28**	MTBE	10	5Å MS (25 mg) + KH_2PO_4 (0.2 equiv)	80
44	DPZ [1.0]	**C28**	MTBE	10	5Å MS (25 mg) + KPF_6 (0.2 equiv)	82
45	DPZ [1.0]	**C28**	MTBE	10	5Å MS (25 mg) + $NaPF_6$ (0.2 equiv)	82
46	DPZ [1.0]	**C28**	MTBE	10	5Å MS (25 mg) + $LiPF_6$ (0.2 equiv)	80
47	DPZ [1.0]	**C28**	MTBE	10	5Å MS (25 mg) + $LiBF_4$ (0.2 equiv)	75
48	DPZ [1.0]	**C28**	MTBE	10	5Å MS (25 mg) + NaBArF (0.2 equiv)	79
49	DPZ [1.0]	**C28**	MTBE	10	5Å MS (25 mg) + NaOAc (0.2 equiv)	78
50	DPZ [1.0]	**C28**	MTBE	10	5Å MS (25 mg) + $PhCO_2Na$ (0.2 equiv)	75
51	DPZ [1.0]	**C28**	MTBE	10	5Å MS (25 mg) + NaF (0.2 equiv)	82
52	DPZ [1.0]	**C28**	MTBE	10	5Å MS (25 mg) + NaBr (0.2 equiv)	82
53	DPZ [1.0]	**C28**	MTBE	10	5Å MS (25 mg) + $Na_2S_2O_4$ (0.2 equiv)	85
54	DPZ [1.0]	**C28**	MTBE	10	5Å MS (25 mg) + $Na_2S_2O_8$ (0.2 equiv)	81
55	DPZ [1.0]	**C28**	MTBE	10	5Å MS (25 mg) + NaI (0.2 equiv)	80
56	DPZ [1.0]	**C28**	MTBE	10	5Å MS (25 mg) + KBr (0.2 equiv)	77
57	DPZ [1.0]	**C28**	MTBE	20	5Å MS (25 mg) + $Na_2S_2O_4$ (0.2 equiv)	80
58	DPZ [1.0]	**C28**	MTBE	15	5Å MS (25 mg) + $Na_2S_2O_4$ (0.2 equiv)	81
59	DPZ [1.0]	**C28**	MTBE	0	5Å MS (25 mg) + $Na_2S_2O_4$ (0.2 equiv)	68
60	DPZ [1.0]	**C28**	CPME	10	5Å MS (25 mg) + $Na_2S_2O_4$ (0.2 equiv)	87
61	DPZ [1.5]	**C28**	CPME	10	5Å MS (25 mg) + $Na_2S_2O_4$ (0.2 equiv)	88
62	DPZ [2.0]	**C28**	CPME	10	5Å MS (25 mg) + $Na_2S_2O_4$ (0.2 equiv)	88
63	DPZ [1.5]	**C28**	CPME	10	5Å MS (25 mg) + TBAB (0.3 equiv)	87
64	DPZ [1.5]	**C28**	CPME	10	5Å MS (25 mg) + TBAC (0.3 equiv)	85
65	DPZ [1.5]	**C28**	CPME	10	5Å MS (25 mg) + TBAI (0.3 equiv)	85
66	DPZ [1.5]	**C28**	CPME	10	5Å MS (25 mg) + TOAB (0.3 equiv)	87
67	DPZ [1.5]	**C28**	CPME	10	5Å MS (25 mg) + TBAB (0.5 equiv)	88
68	DPZ [1.5]	**C28**	CPME	10	5Å MS (25 mg) + TBAB (1.0 equiv)	87

69	DPZ [1.5]	**C1**	CPME	10	5Å MS (25 mg) + TBAB (0.3 equiv)	89
70	DPZ [1.5]	**C1**	CPME	10	5Å MS (25 mg) + TBPB (0.3 equiv)	90
71	DPZ [1.5]	**C1**	CPME	10	5Å MS (25 mg) + $Na_2S_2O_4$ (0.2 equiv)	88
72	DPZ [1.5]	**C1**	CPME	10	5Å MS (25 mg) + $Na_2S_2O_4$ (0.5 equiv)	89
73	DPZ [1.5]	**C1**	CPME	10	5Å MS (25 mg) + $Na_2S_2O_4$ (1.0 equiv)	88
74	DPZ [1.5]	**C1**	CPME	10	5Å MS (25 mg) + $Na_2S_2O_4$ (0.5 equiv) + TBPB (0.3 equiv)	93[d]
75	DPZ [1.5]	**C2**	CPME	10	5Å MS (25 mg) + $Na_2S_2O_4$ (0.5 equiv) + TBPB (0.3 equiv)	76
76	DPZ [1.5]	**C3**	CPME	10	5Å MS (25 mg) + $Na_2S_2O_4$ (0.5 equiv) + TBPB (0.3 equiv)	67
77	DPZ [1.5]	**C29**	CPME	10	5Å MS (25 mg) + $Na_2S_2O_4$ (0.5 equiv) + TBPB (0.3 equiv)	72[e]
78	Ru^{2+} [1.5][f]	**C3**	CPME	10	5Å MS (25 mg) + $Na_2S_2O_4$ (0.5 equiv) + TBPB (0.3 equiv)	89
79	RB [1.5][g]	**C3**	CPME	10	5Å MS (25 mg) + $Na_2S_2O_4$ (0.5 equiv) + TBPB (0.3 equiv)	91

[a] Reaction conditions: **1a** (0.075 mmol), **2a** (0.05 mmol), chiral catalyst (10 mol%), degassed solvent (1.0 mL), irradiation with blue LED (3 W, 450 nm), 12−36 h. [b] Determined by HPLC analysis on a chiral stationary phase. [c] Yield = 62% (Isolated by flash column chromatography on *silica gel*). [d] Yield = 78% (Isolated by flash column chromatography on *silica gel*). [e] Yield = 76% (Isolated by flash column chromatography on *silica gel*). [f] $Ru(bpy)_2Cl_2{\bullet}6H_2O$. [g] RB = Rose Bengal.

3. General experimental procedures

(1) General procedure for asymmetric radical−radical cross−coupling of *N*-aryl glycines 1 with 1,2-diketones 2

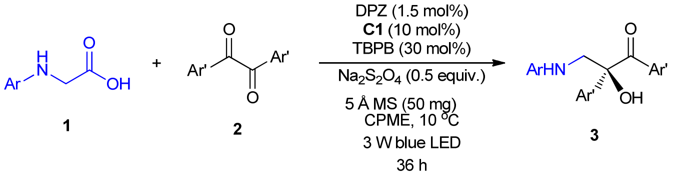

106.3 μL (0.0015 mmol, 0.015 equiv) of DPZ solution (1.0 mg of DPZ in 200 μL of toluene) was added into a 10 mL Schlenk tube, and then solvent was removed in *vacuo*. Subsequently, **1** (0.15 mmol, 1.5 equiv), **2** (0.10 mmol, 1.0 equiv), **C1** (0.01 mmol, 0.10 equiv), TBPB (0.03 mmol, 0.3 equiv), $Na_2S_2O_4$ (0.05 mmol, 0.5 equiv), 5Å MS (50 mg) and CPME (2.0 mL) were sequentially added, degassed three times by freeze-pump-thaw method. The reaction mixture was stirred under an argon atmosphere at 10 °C (the temperature was maintained in an incubator) and in dark for 30 min, then irradiated by a 3 W blue LED (λ = 450−455 nm) from a 2.0 cm distance for another 36 h. The reaction mixture was directly loaded onto a short *silica gel* column, followed by gradient elution with petroleum ether/ethyl acetate (100/1–50/1 ratio). Removing the solvent in *vacuo*, afforded products **3a**−**3q**.

For product **3r**, the procedure is similar but with the following reaction conditions: **1** (0.15 mmol), **2** (0.1 mmol), DPZ (1.5 mol%), **C2** (10 mol%), 4 Å MS (50 mg), CPME (2.0 mL), −5 °C, degassed and under an argon atmosphere, 36 h.

(2) General procedure for asymmetric radical−radical cross−coupling of *N*-aryl glycine 1h with isatins 4

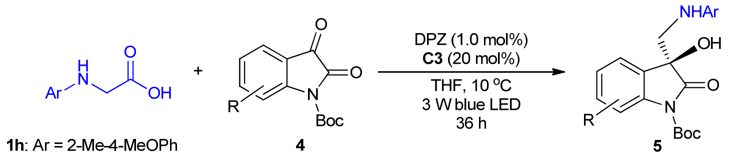

70.9 μL (0.001 mmol, 0.01 equiv) of DPZ solution (1.0 mg of DPZ in 200 μL of toluene) was added into a 10 mL Schlenk tube, and then solvent was removed in *vacuo*. Subsequently, **1h** (0.15 mmol, 1.5 equiv), **4** (0.10 mmol, 1.0 equiv), **C3** (0.02 mmol, 0.2 equiv) and THF (2.0 mL) were sequentially added, degassed three times by freeze-pump-thaw method. The reaction mixture was stirred under an argon atmosphere at 10 °C (the temperature was maintained in an incubator) and in dark for 30 min, then irradiated by a 3 W blue LED (λ =

450−455 nm) from a 5.0 cm distance for another 36 h. The reaction mixture was directly loaded onto a short *silica gel* column, followed by gradient elution with petroleum ether/ethyl acetate (80/1–5/1 ratio). Removing the solvent in *vacuo*, afforded products **5a−5l**.

4. Mechanism studies

Emission quenching experiments

Emission intensities were recorded on a spectrofluorometer. DPZ solution was excited at 448 nm and the emission intensity at 544 nm was observed. The appropriate amount of quencher was added to a solution of DPZ (5.0×10^{-5} M) in CPME in 5.0 mL volumetric flask under N_2. The solution was transferred to a 1.5 mL quartz cell and the emission spectrum of the sample was collected.

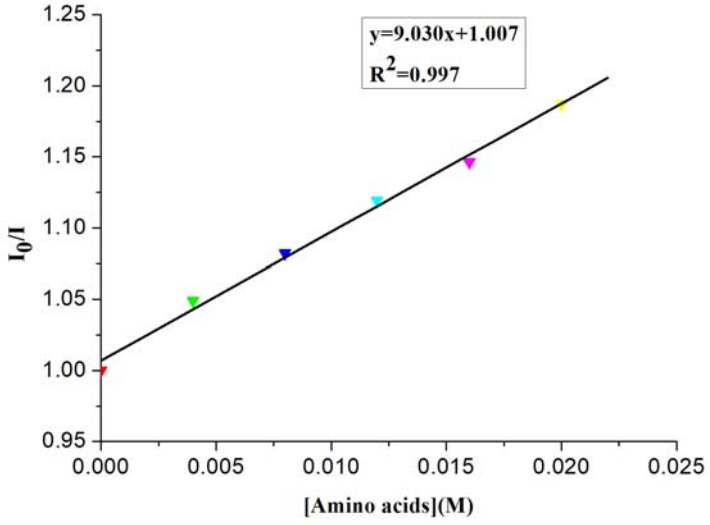

Fig. S1. Stern–Volmer quenching experiment of DPZ and **1a**.

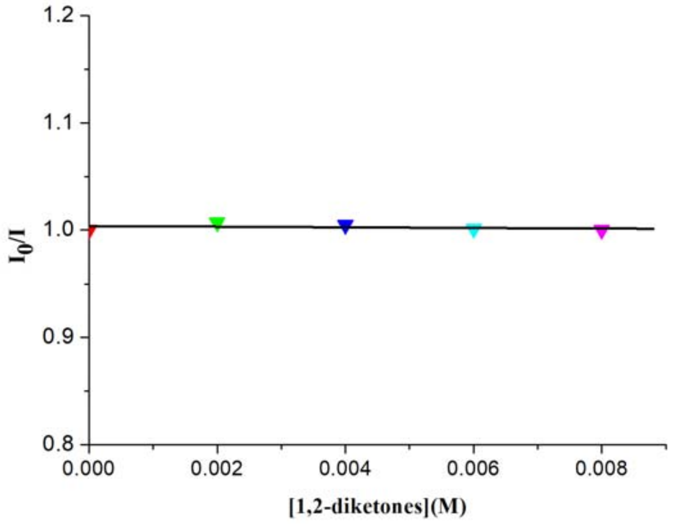

Fig. S2. Stern–Volmer quenching experiment of DPZ and **2a**. No quenching observed.

5. Procedures of synthetic applications

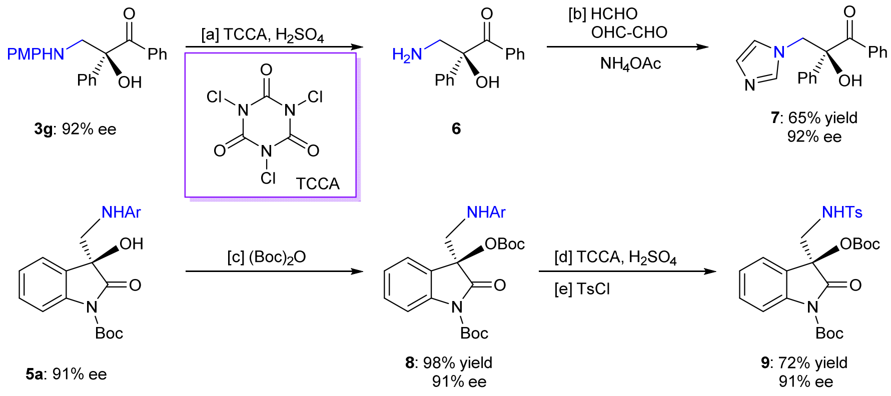

Step A: To a solution of **3g** (0.4 mmol, 1.0 equiv) in 4.0 mL acetonitrile and 4.0 mL water, TCCA (0.2 mmol, 0.5 equiv) and 0.4 mL H_2SO_4 (1.0 M) were added at 0 °C. The mixture was stirred for 16 h at room temperature. After removal of acetonitrile by evaporation, the resulting aqueous phase washed with 4.0 mL CH_2Cl_2 for three times. The resulting aqueous phase was subsequently regulated to pH 11.0 through addition of saturated Na_2CO_3 solution. The aqueous solution was extracted with 5.0 mL EtOAc for three times. The combined organic layers were dried over Na_2SO_4 and concentrated in *vacuo* afforded product **6**, which was used for the next step without further purification.

Step B: Product **6**, glyoxal (0.8 mmol, 2.0 equiv), formaldehyde (0.8 mmol, 2.0 equiv), ammonium chloride (0.8 mmol, 2.0 equiv) was dissolved in methanol (0.5 mL). The reaction was heated at 80 °C for 5 h. After cooling to room temperature, the solvent was evaporated, NaOH (2.0 *N*, aq.) was added and the product was extracted with DCM (5 x 5 mL). The combined organic layers were dried over Na_2SO_4, concentrated in *vacuo*. The reaction mixture was directly loaded onto a short *silica gel* column, followed by gradient elution with dichloromethane/methanol (100/1–30/1 ratio). Removing the solvent in *vacuo*, afforded product **7** in 65% of yield over two steps and with 92% ee.

Step C: To a solution of **5a** (0.4 mmol, 1.0 equiv) in 4.0 mL DCM, DMAP (0.08 mmol, 0.2 equiv) and di-*tert*-butyl dicarbonate (0.44 mmol, 1.1 equiv) were added at 0 °C. The mixture was stirred for 0.5 h at room temperature. The reaction mixture was directly loaded onto a short *silica gel* column, followed by gradient elution with petroleum ether/ethyl acetate (30/1–20/1 ratio). Removing the solvent in *vacuo*, afforded product **8** in 98% yield.

Steps D and E: To a solution of **8** (0.4 mmol, 1.0 equiv) in 4.0 mL acetonitrile and 4.0 mL

water, TCCA (0.2 mmol, 0.5 equiv) and 0.4 mL H_2SO_4 (1.0 M) were added at 0 ^{o}C. The reaction worked for 16 h at room temperature. The reaction mixture was subsequently regulated to pH 11.0 through adding saturated Na_2CO_3 solution. The aqueous solution was extracted with 5.0 mL EtOAc for three times. The combined organic layers were dried over Na_2SO_4 and concentrated in *vacuo*. The ethyl acetate solution of amine was cooled to 0 °C, and then triethylamine (0.8 mmol, 2.0 equiv) with 4-tosyl chloride (0.8 mmol, 2.0 equiv) were added. The reaction was stirred for 5 h at room temperature. The reaction mixture was directly loaded onto a short *silica gel* column, followed by gradient elution with petroleum ether/ethyl acetate (30/1–5/1 ratio). Removing the solvent in *vacuo*, afforded product **9** in 72% of yield over two steps and with 91% ee.

6. Determination of the absolute configurations

Absolute configurations of **3a-p**, **3r** and **7** are determined by *X*-ray structure analysis of the product **3q**.

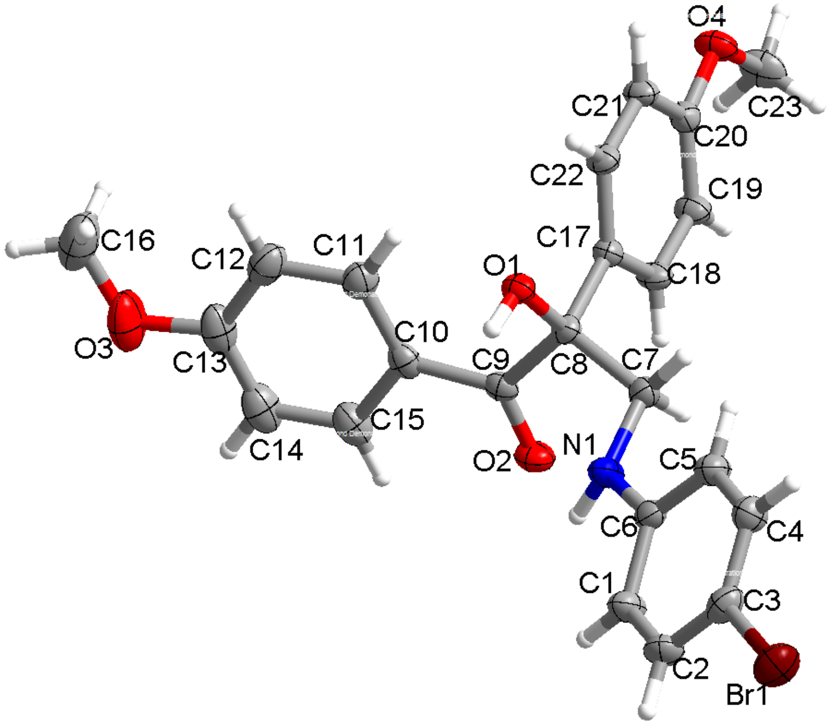

Fig. S3 Absolute configuration of **3q** (CCDC 1836991).

Displacement ellipsoids are drawn at the 30% probability level. (Solvent: EA:PE = 1:10)

Absolute configurations of **5a-l** are determined by *X*-ray structure analysis of the product **9**.

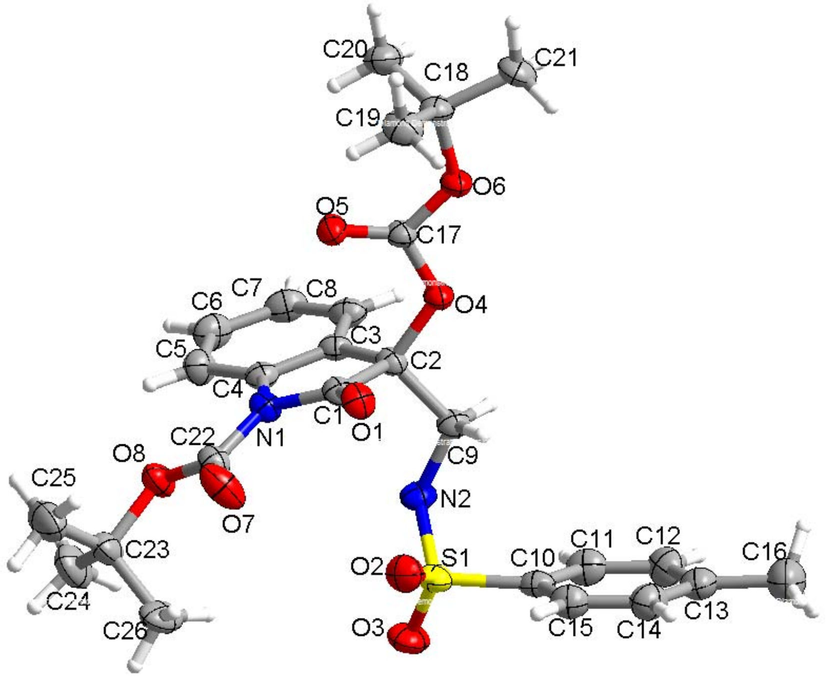

Fig. S4 Absolute configuration of **11** (CCDC 1843336).

Displacement ellipsoids are drawn at the 30% probability level. (Solvent: dichloromethane)

7. Characterization of adducts

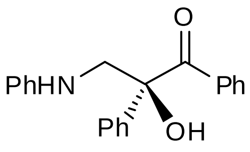

3a

Yellow oil; 27.0 mg, 85% yield; 93% ee; $[\alpha]_D^{22}$ +195.8 (*c* 2.0, $CHCl_3$); ^{1}H NMR (300 MHz, $CDCl_3$) δ 7.79 (d, *J* = 7.4 Hz, 2H), 7.47 (d, *J* = 7.2 Hz, 2H), 7.37 – 7.13 (m, 6H), 7.06 (t, *J* = 7.9 Hz, 2H), 6.67 (t, *J* = 7.3 Hz, 1H), 6.59 (d, *J* = 7.8 Hz, 2H), 4.54 (s, 1H), 4.05 (d, *J* = 12.9 Hz, 1H), 3.42 (d, *J* = 12.9 Hz, 1H); ^{13}C NMR (75 MHz, $CDCl_3$) δ 200.7, 148.3, 140.2, 134.5, 133.0, 130.6, 129.4, 129.2, 128.4, 128.3, 125.2, 119.2, 114.6, 82.4, 53.7; HRMS (ESI) m/z 318.1489 (M+H$^+$), calc. for $C_{21}H_{20}N_1O_2$ 318.1494.

The ee was determined by HPLC analysis: CHIRALPAK IC (4.6 mm i.d. x 250 mm); Hexane/2-propanol = 95/5; flow rate 1.0 mL/min; 25 °C; 254 nm; retention time: 7.1 min (major) and 8.5 min (minor).

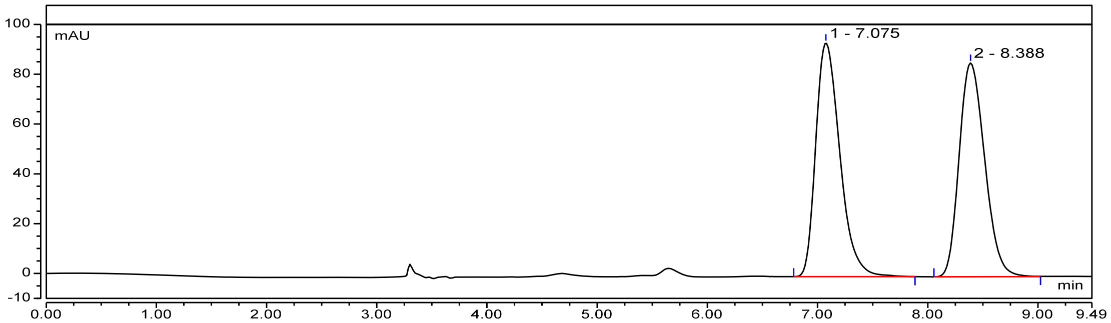

Entry	Retention Time	Area	Height	%Area
1	7.075	23.8053	93.94	50.81
2	8.388	23.0462	85.84	49.19

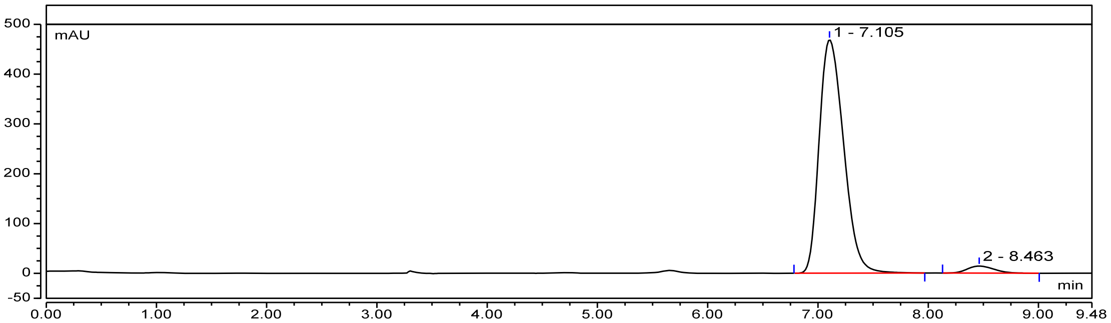

Entry	Retention Time	Area	Height	%Area
1	7.105	120.5333	468.63	96.78
2	8.463	4.0084	14.23	3.22

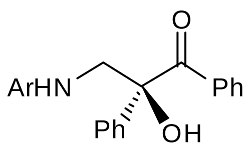

3b: Ar = 4-ClPh

Yellow solid; Mp 74.7 – 75.2 °C; 29.2 mg, 83% yield; 90% ee; $[\alpha]_D^{22}$ +140.4 (*c* 2.0, $CHCl_3$); ^1H NMR (300 MHz, CDCl3) δ 7.76 (d, *J* = 7.6 Hz, 2H), 7.45 (d, *J* = 7.2 Hz, 2H), 7.41 – 7.16 (m, 6H), 7.00 (d, *J* = 8.7 Hz, 2H), 6.50 (d, *J* = 8.8 Hz, 2H), 4.49 (s, 1H), 4.02 (d, *J* = 12.8 Hz, 1H), 3.41 (d, J = 12.8 Hz, 1H); ^{13}C NMR (75 MHz, $CDCl_3$) δ 200.6, 146.8, 139.9, 134.2, 133.1, 130.5, 129.2, 128.5, 128.3, 125.2, 123.7, 115.6, 82.2, 53.6; HRMS (ESI) m/z 352.1113 ($M+H^+$), calc. for $C_{21}H_{19}N_1O_2Cl_1$ 352.1104.

The ee was determined by HPLC analysis: CHIRALPAK IC (4.6 mm i.d. x 250 mm); Hexane/2-propanol = 95/5; flow rate 1.0 mL/min; 25 °C; 254 nm; retention time: 7.5 min (major) and 9.2 min (minor).

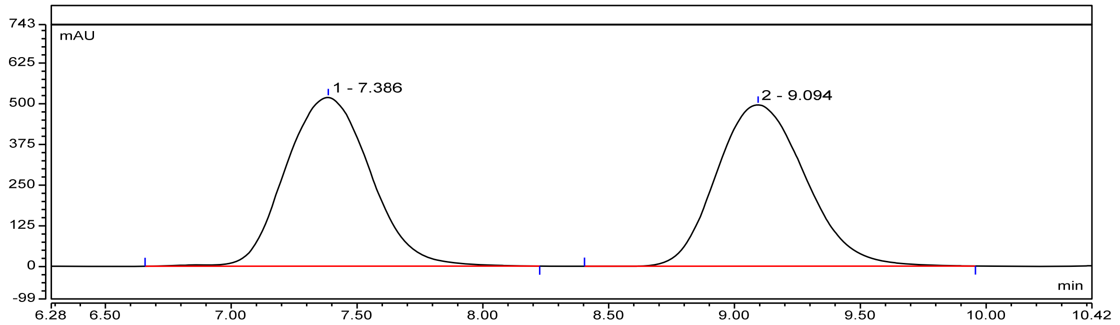

Entry	Retention Time	Area	Height	%Area
1	7.386	202.8690	518.39	49.98
2	9.094	203.0450	495.29	50.02

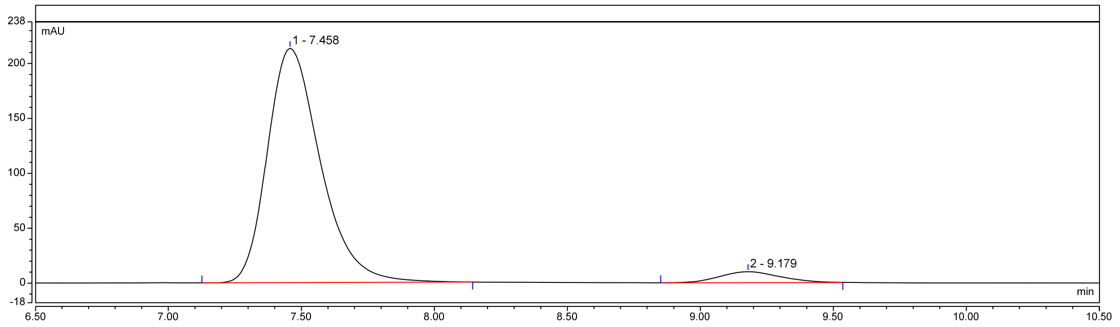

Entry	Retention Time	Area	Height	%Area
1	7.458	49.3688	213.11	94.99
2	9.179	2.6047	9.92	5.01

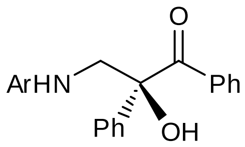

3c: Ar = 4-BrPh

Yellow solid; Mp 74.5 − 75.7 °C; 33.7 mg, 85% yield; 91% ee; $[\alpha]_D^{22}$ +137.7 (*c* 2.0, $CHCl_3$); ^1H NMR (300 MHz, $CDCl_3$) δ 7.85 (d, *J* = 7.5 Hz, 2H), 7.55 (d, *J* = 7.2 Hz, 2H), 7.50 – 7.20 (m, 8H), 6.55 (d, *J* = 8.8 Hz, 2H), 4.57 (s, 1H), 4.11 (d, *J* = 12.8 Hz, 1H), 3.51 (d, *J* = 12.8 Hz, 1H); ^{13}C NMR (75 MHz, $CDCl_3$) δ 200.6, 147.2, 139.8, 134.2, 133.1, 132.1, 130.5, 129.2,

128.5, 128.3, 125.2, 116.0, 110.8, 82.2, 53.4; HRMS (ESI) m/z 396.0598 (M+H$^+$), calc. for $C_{21}H_{19}N_1O_2Br_1$ 396.0599.

The ee was determined by HPLC analysis: CHIRALPAK IC (4.6 mm i.d. x 250 mm); Hexane/2-propanol = 95/5; flow rate 1.0 mL/min; 25 ^{o}C; 254 nm; retention time: 7.8 min (major) and 9.7 min (minor).

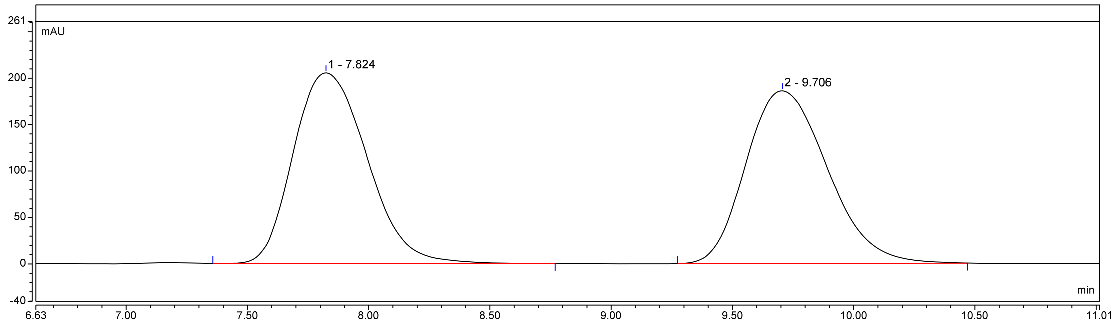

Entry	Retention Time	Area	Height	%Area
1	7.824	72.2412	205.21	49.92
2	9.706	72.4748	185.87	50.08

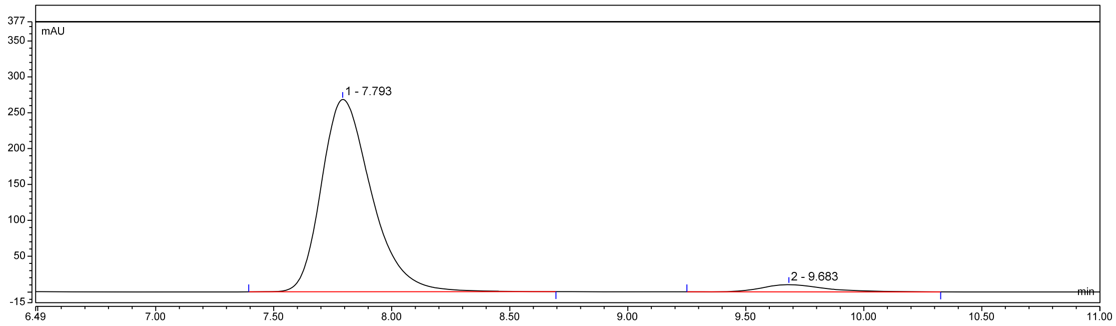

Entry	Retention Time	Area	Height	%Area
1	7.793	65.9559	267.97	95.47
2	9.683	3.1275	10.06	4.53

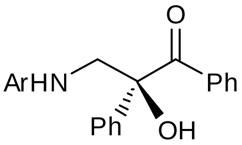

3d: Ar = 3-ClPh

Yellow oil; 31.3 mg, 89% yield; 90% ee; $[\alpha]_D^{22}$ +120.3 (*c* 2.0, $CHCl_3$); ^{1}H NMR (300 MHz, $CDCl_3$) δ 7.74 (d, *J* = 7.4 Hz, 2H), 7.45 (d, *J* = 7.1 Hz, 2H), 7.41 – 7.15 (m, 6H), 6.95 (t, *J* = 8.0 Hz, 1H), 6.62 (d, *J* = 7.9 Hz, 1H), 6.54 (d, *J* = 1.9 Hz, 1H), 6.44 (dd, *J* = 8.2, 1.6 Hz, 1H), 4.45 (s, 1H), 4.03 (m, 2H), 3.45 (d, *J* = 12.8 Hz, 1H); ^{13}C NMR (75 MHz, $CDCl_3$) δ 200.5, 149.4, 139.8, 135.1, 134.2, 133.2, 130.4, 130.3, 129.2, 128.6, 128.3, 125.3, 118.9, 114.0, 112.8, 82.2, 53.0; HRMS (ESI) m/z 352.1098 (M+H$^+$), calc. for $C_{21}H_{19}N_1O_2Cl_1$ 352.1104.

The ee was determined by HPLC analysis: CHIRALPAK IC (4.6 mm i.d. x 250 mm); Hexane/2-propanol = 95/5; flow rate 1.0 mL/min; 25 ^{o}C; 254 nm; retention time: 7.4 min (major) and 10.3 min (minor).

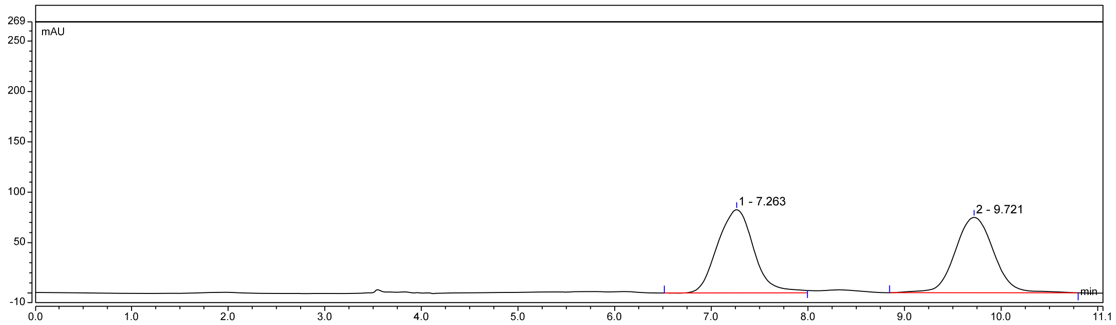

Entry	Retention Time	Area	Height	%Area
1	7.263	36.0975	82.57	50.97
2	9.721	34.7255	74.73	49.03

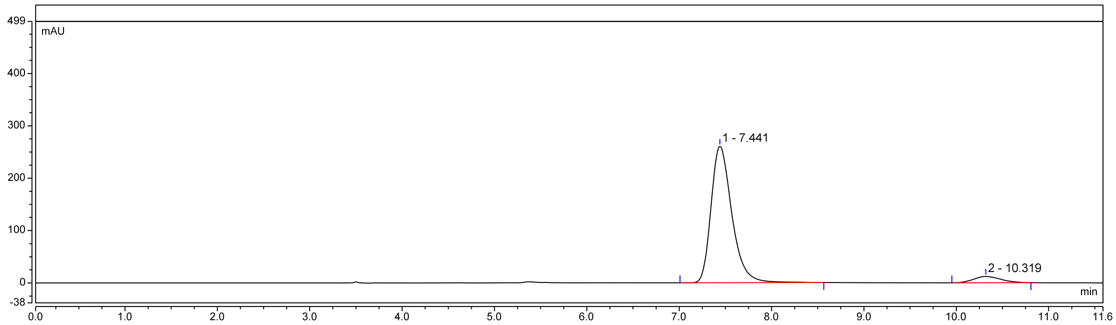

Entry	Retention Time	Area	Height	%Area
1	7.441	68.0407	260.49	94.88
2	10.319	3.6742	11.66	5.12

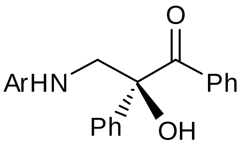

3e: Ar = 4-MePh

Yellow oil; 28.2 mg, 85% yield; 91% ee; $[\alpha]_D^{22}$ +94.2 (*c* 2.0, $CHCl_3$); ^1H NMR (300 MHz, $CDCl_3$) δ 7.79 (d, *J* = 7.5 Hz, 2H), 7.46 (d, *J* = 7.3 Hz, 2H), 7.33 (m, 3H), 7.19 (m, 3H), 6.88 (d, *J* = 8.1 Hz, 2H), 6.53 (d, *J* = 8.3 Hz, 2H), 4.62 (s, 1H), 4.03 (d, *J* = 12.7 Hz, 1H), 3.35 (d, *J* = 12.7 Hz, 1H), 2.14 (s, 3H); ^{13}C NMR (75 MHz, $CDCl_3$) δ 200.6, 145.7, 140.1, 134.3, 132.9, 130.5, 129.8, 129.0, 128.6, 128.3, 128.1, 125.0, 114.9, 82.1, 54.3, 20.5; HRMS (ESI) m/z 332.1660 (M+H$^+$), calc. for $C_{22}H_{22}N_1O_2$ 332.1651.

The ee was determined by HPLC analysis: CHIRALPAK IC (4.6 mm i.d. x 250 mm); Hexane/2-propanol = 90/10; flow rate 1.0 mL/min; 25 °C; 254 nm; retention time: 6.5 min (major) and 7.9 min (minor).

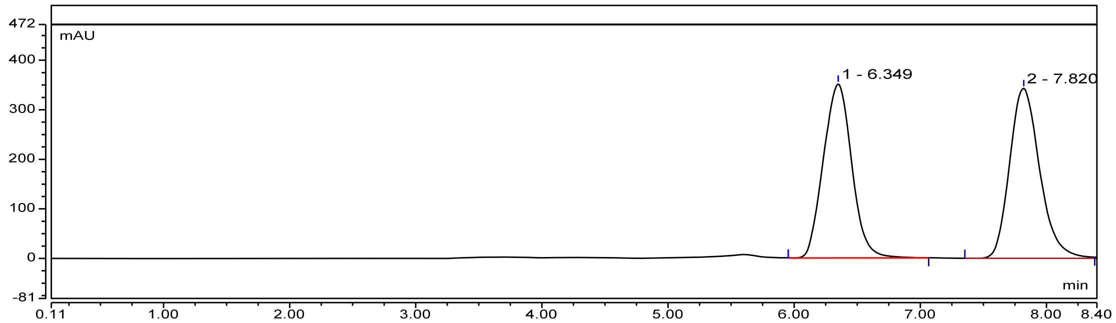

Entry	Retention Time	Area	Height	%Area
1	6.349	91.4757	350.96	49.09
2	7.820	94.8612	342.96	50.91

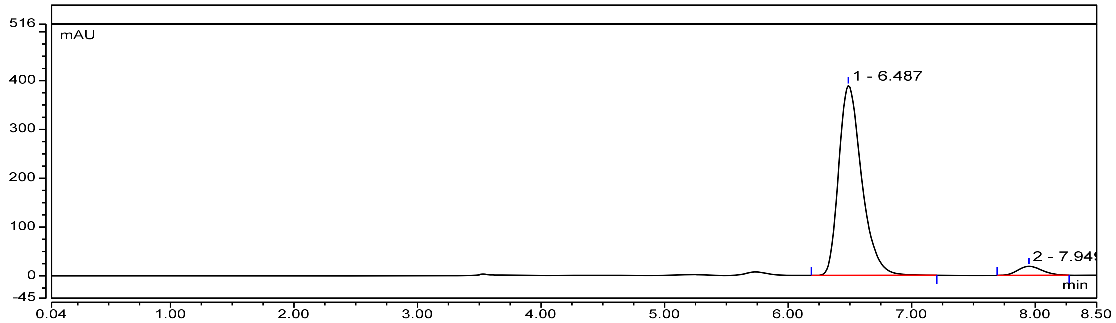

Entry	Retention Time	Area	Height	%Area
1	6.487	83.8961	388.80	95.45
2	7.949	4.0032	18.08	4.55

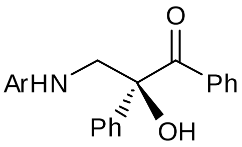

3f: Ar = 3-MePh

Yellow solid; Mp 73.0 − 74.6 °C; 25.8 mg, 78% yield; 90% ee; $[\alpha]_D^{22}$ +83.2 (*c* 2.0, $CHCl_3$); ^{1}H NMR (300 MHz, $CDCl_3$) δ 7.89 (d, *J* = 7.4 Hz, 2H), 7.57 (d, *J* = 7.2 Hz, 2H), 7.48 – 7.27 (m, 6H), 7.06 (m, 1H), 6.61 (d, *J* = 7.4 Hz, 1H), 6.52 (s, 2H), 4.63 (s, 1H), 4.14 (d, *J* = 12.8 Hz, 1H), 3.51 (d, *J* = 12.8 Hz, 1H), 2.26 (s, 3H); ^{13}C NMR (75 MHz, $CDCl_3$) δ 200.6, 148.2, 140.2, 139.3, 134.4, 33.0, 130.6, 129.3, 129.1, 128.4, 128.2, 125.2, 120.1, 115.4, 111.8, 82.2, 53.8, 21.7; HRMS (ESI) m/z 332.1652 (M+H$^+$), calc. for $C_{22}H_{22}N_1O_2$ 332.1651.

The ee was determined by HPLC analysis: CHIRALPAK IC (4.6 mm i.d. x 250 mm); Hexane/2-propanol = 95/5; flow rate 1.0 mL/min; 25 °C; 254 nm; retention time: 7.3 min (major) and 8.9 min (minor).

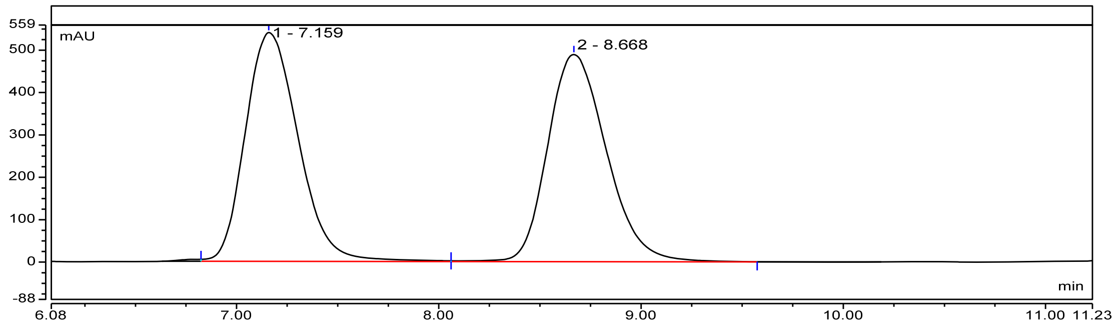

Entry	Retention Time	Area	Height	%Area
1	7.159	163.8425	539.85	49.93
2	8.668	164.2897	488.66	50.07

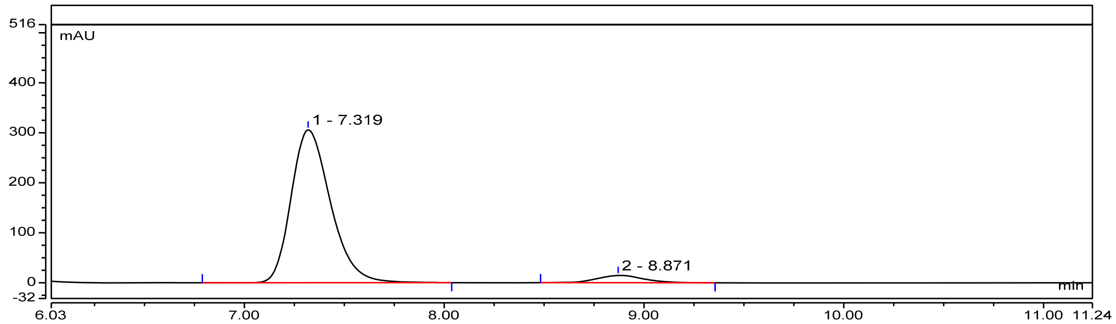

Entry	Retention Time	Area	Height	%Area
1	7.319	69.7140	305.77	94.97
2	8.871	3.6911	14.32	5.03

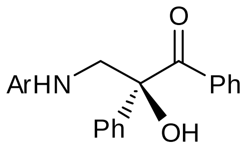

3g: Ar = 4-OMePh

Yellow oil; 29.5 mg, 85% yield; 92% ee; $[\alpha]_D^{22}$ +36.2 (*c* 2.0, $CHCl_3$); ^{1}H NMR (300 MHz, $CDCl_3$) δ 7.89 (d, *J* = 7.6 Hz, 2H), 7.56 (d, *J* = 7.3 Hz, 2H), 7.34 (m, 6H), 6.71 (dd, *J* = 27.5, 8.9 Hz, 4H), 4.79 (s, 1H), 4.12 (d, *J* = 12.6 Hz, 1H), 3.74 (s, 3H), 3.38 (d, *J* = 12.6 Hz, 1H); ^{13}C NMR (75 MHz, $CDCl_3$) δ 200.6, 153.4, 142.1, 140.3, 134.4, 133.0, 130.6, 129.1, 128.3, 128.2, 125.1, 116.4, 114.9, 82.1, 55.8, 55.3; HRMS (ESI) m/z 348.1614 (M+H$^+$), calc. for $C_{22}H_{22}N_1O_3$ 348.1600.

The ee was determined by HPLC analysis: CHIRALPAK IC (4.6 mm i.d. x 250 mm); Hexane/2-propanol = 95/5; flow rate 1.0 mL/min; 25 ^{o}C; 254 nm; retention time: 13.0 min (major) and 18.6 min (minor).

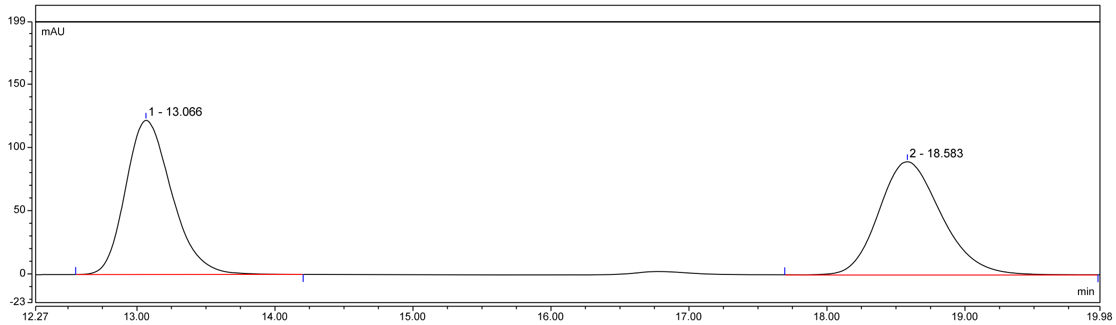

Entry	Retention Time	Area	Height	%Area
1	13.066	47.3003	121.84	49.94
2	18.583	47.4123	89.47	50.06

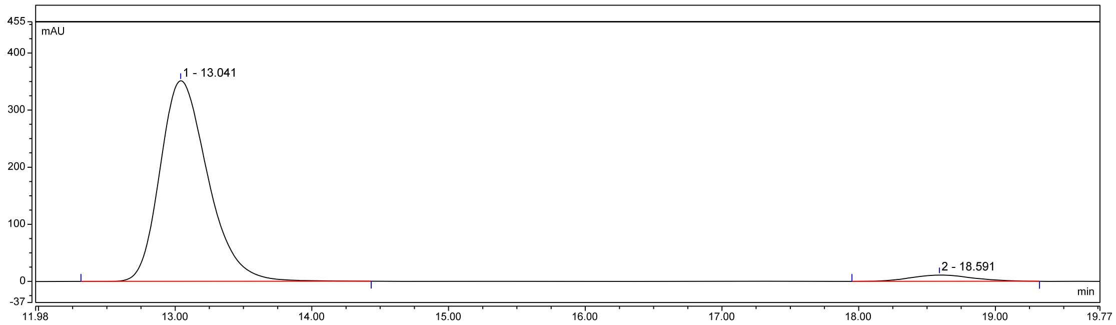

Entry	Retention Time	Area	Height	%Area
1	13.041	142.1831	351.23	96.10
2	18.591	5.7710	11.04	3.90

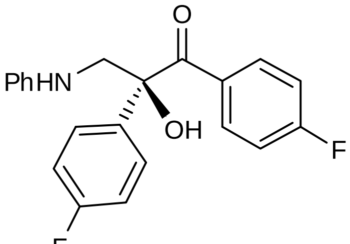

3h

Yellow oil; 24.4 mg, 69% yield; 88% ee; $[\alpha]_D^{22}$ +62.8 (*c* 2.0, $CHCl_3$); ^1H NMR (300 MHz, $CDCl_3$) δ 7.98 (m, 2H), 7.53 (m, 2H), 7.19 (t, *J* = 7.8 Hz, 2H), 7.10 (t, *J* = 8.6 Hz, 2H), 6.99 (t, *J* = 8.6 Hz, 2H), 6.80 (t, *J* = 7.3 Hz, 1H), 6.72 (d, *J* = 8.0 Hz, 2H), 4.58 (s, 1H), 4.12 (d, *J* = 13.0 Hz, 1H), 3.40 (d, *J* = 13.0 Hz, 1H); ^{13}C NMR (75 MHz, $CDCl_3$) δ 198.6, 167.3, 164.3, 163.9, 161.1, 148.1, 135.8, 135.7, 133.6, 135.5, 130.5, 130.4, 129.5, 126.8, 126.7, 119.5, 116.3, 116.0, 115.6, 115.3, 114.7, 82.0, 54.3; HRMS (ESI) m/z 354.1308 ($M+H^+$), calc. for $C_{21}H_{18}N_1O_2F_2$ 354.1306.

The ee was determined by HPLC analysis: CHIRALPAK cellulose-3 (4.6 mm i.d. x 250 mm); Hexane/2-propanol = 90/10; flow rate 1.0 mL/min; 25 ^oC; 254 nm; retention time: 18.6 min (minor) and 22.0 min (major).

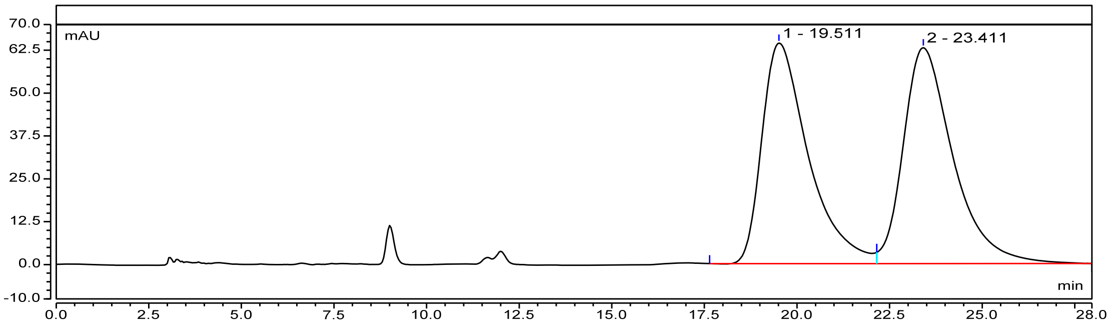

Entry	Retention Time	Area	Height	%Area
1	19.511	93.4565	64.37	48.98
2	23.411	97.3659	62.88	51.02

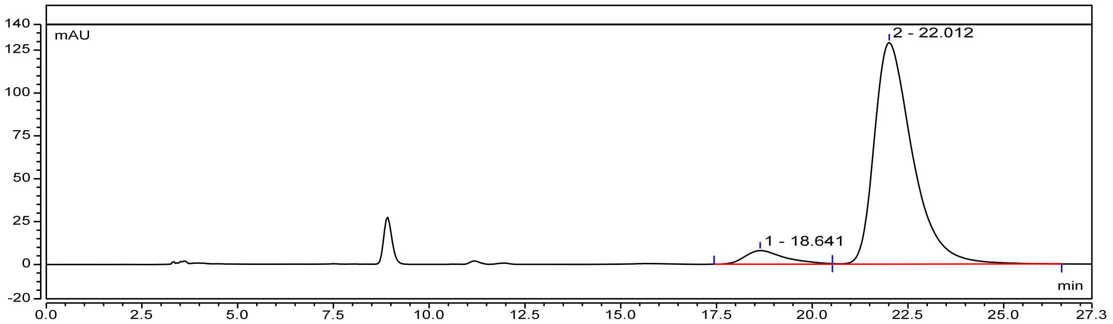

Entry	Retention Time	Area	Height	%Area
1	18.641	9.6307	7.93	6.08
2	22.012	148.6794	129.21	93.92

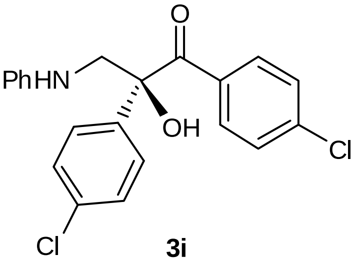

3i

Yellow oil; 27.8 mg, 72% yield; 84% ee; $[\alpha]_D^{22}$ +157.9 (*c* 2.0, $CHCl_3$); ^{1}H NMR (300 MHz, $CDCl_3$) δ 7.80 (d, *J* = 8.6 Hz, 2H), 7.40 (d, *J* = 8.6 Hz, 2H), 7.30 (d, *J* = 8.6 Hz, 2H), 7.20 (m, 2H), 7.11 (t, *J* = 7.9 Hz, 2H), 6.73 (t, *J* = 7.3 Hz, 1H), 6.64 (d, *J* = 7.9 Hz, 2H), 4.47 (s, 1H), 4.03 (d, *J* = 13.1 Hz, 1H), 3.29 (d, *J* = 13.1 Hz, 1H); ^{13}C NMR (75 MHz, $CDCl_3$) δ 198.8, 148.0, 139.7, 138.4, 134.5, 132.4, 132.2, 129.5, 129.4, 128.6, 126.3, 119.6, 114.8, 82.1, 54.2; HRMS (ESI) m/z 386.0719 (M+H$^+$), calc. for $C_{21}H_{18}N_1O_2Cl_2$ 386.0715.

The ee was determined by HPLC analysis: CHIRALPAK IB (4.6 mm i.d. x 250 mm); Hexane/2-propanol = 90/10; flow rate 1.0 mL/min; 25 ^{o}C; 254 nm; retention time: 8.4 min (minor) and 9.9 min (major).

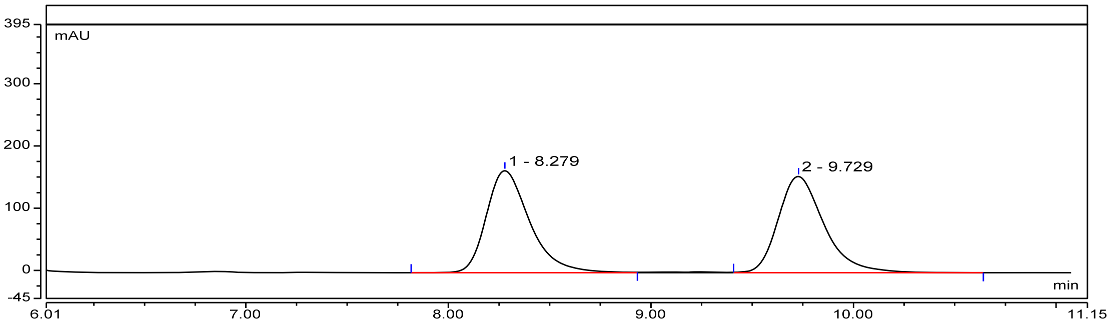

Entry	Retention Time	Area	Height	%Area
1	8.279	40.7934	163.78	50.13
2	9.729	40.5842	154.52	49.87

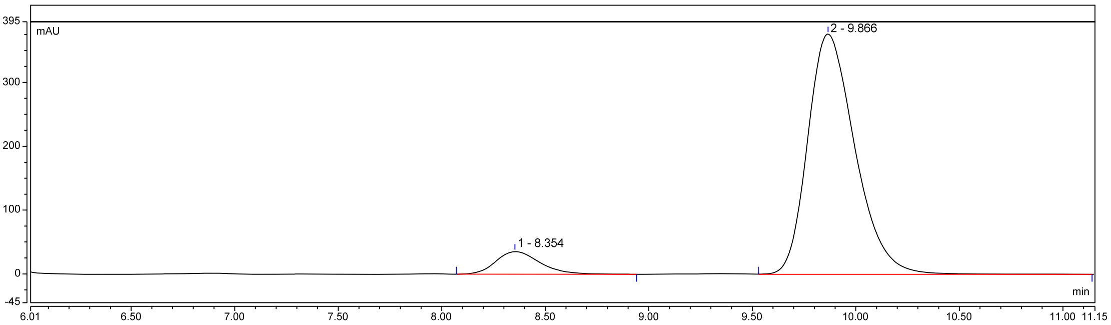

Entry	Retention Time	Area	Height	%Area
1	8.354	8.6818	35.45	8.02
2	9.866	99.5794	376.29	91.98

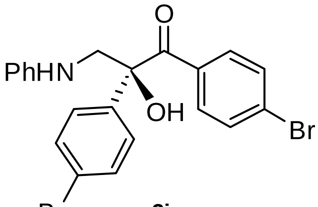

3j

Yellow oil; 36.1 mg, 76% yield; 86% ee; $[\alpha]_D^{22}$ +86.4 (*c* 2.0, $CHCl_3$); ^{1}H NMR (300 MHz, $CDCl_3$) δ 7.80 (d, *J* = 8.6 Hz, 2H), 7.54 (d, *J* = 8.6 Hz, 2H), 7.44 (m, 4H), 7.19 (t, *J* = 7.9 Hz, 2H), 6.81 (t, *J* = 7.3 Hz, 1H), 6.72 (d, *J* = 7.8 Hz, 2H), 4.53 (s, 1H), 4.11 (d, *J* = 13.1 Hz, 1H), 3.36 (d, *J* = 13.1 Hz, 1H); ^{13}C NMR (75 MHz, $CDCl_3$) δ 199.1, 148.1, 139.0, 132.9, 132.5, 132.3, 131.7, 129.6, 128.7, 126.7, 122.8, 119.8, 114.9, 82.3, 54.3. The ee was determined by HPLC analysis: CHIRALPAK cellulose-4 (4.6 mm i.d. x 250 mm); Hexane/2-propanol = 95/5; flow rate 1.0 mL/min; 25 ^{o}C; 254 nm; retention time: 9.3 min (minor) and 10.7 min (major) ; HRMS (ESI) m/z 473.9696 (M+H$^+$), calc. for $C_{21}H_{18}N_1O_2Br_2$ 473.9704.

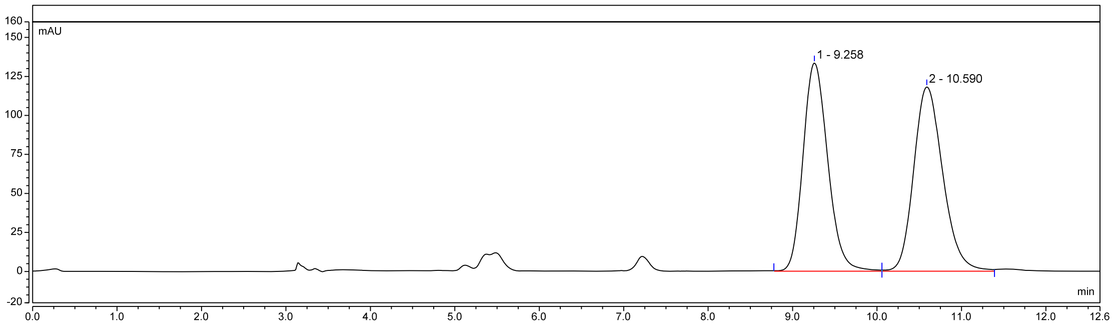

Entry	Retention Time	Area	Height	%Area
1	9.258	45.3990	133.40	49.04
2	10.590	47.1722	118.03	50.96

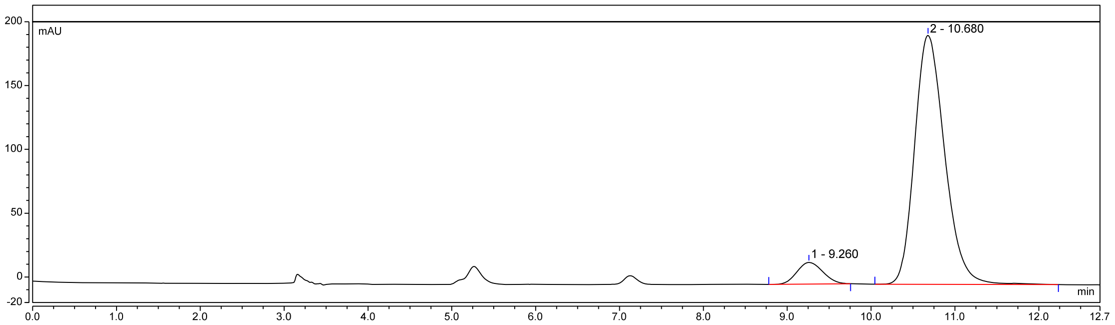

Entry	Retention Time	Area	Height	%Area
1	9.260	6.1037	17.07	7.00
2	10.680	81.1539	195.05	93.00

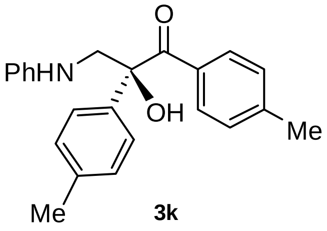

3k

Yellow solid; Mp 71.4 − 72.5 °C; 28.0 mg, 81% yield; 93% ee; $[\alpha]_D^{22}$ +156.7 (*c* 2.0, $CHCl_3$); ^{1}H NMR (300 MHz, $CDCl_3$) δ 7.83 (d, *J* = 8.2 Hz, 2H), 7.45 (d, *J* = 8.1 Hz, 2H), 7.27 – 7.10 (m, 6H), 6.78 (t, *J* = 7.3 Hz, 1H), 6.71 (d, *J* = 7.9 Hz, 2H), 4.68 (s, 1H), 4.13 (d, *J* = 12.7 Hz, 1H), 3.55 (d, *J* = 12.7 Hz, 1H), 2.37 (s, 3H), 2.34 (s, 3H); ^{13}C NMR (75 MHz, $CDCl_3$) δ 200.2, 148.3, 143.9, 138.1, 137.4, 131.7, 130.7, 129.8, 129.4, 128.9, 125.1, 119.0, 114.5, 82.0, 53.6, 21.7, 21.3; HRMS (ESI) m/z 346.1808 ($M+H^+$), calc. for $C_{23}H_{24}N_1O_2$ 346.1807.

The ee was determined by HPLC analysis: CHIRALPAK IC (4.6 mm i.d. x 250 mm); Hexane/2-propanol = 95/5; flow rate 1.0 mL/min; 25 °C; 254 nm; retention time: 9.7 min (major) and 12.3 min (minor).

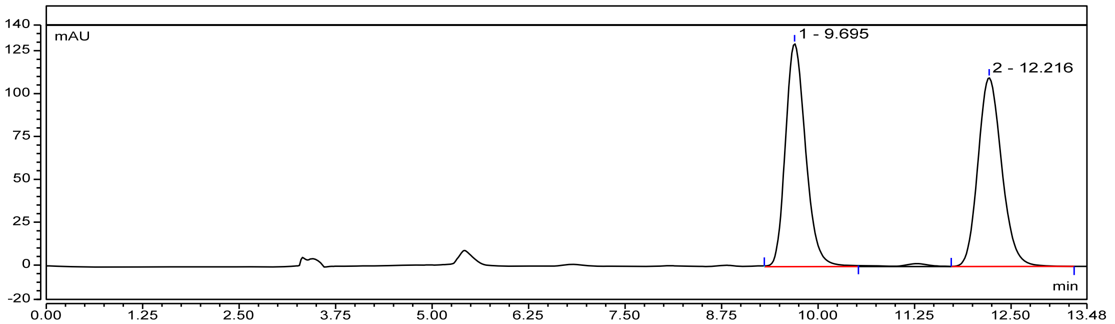

Entry	Retention Time	Area	Height	%Area
1	9.695	39.5473	130.10	50.21
2	12.216	39.2218	110.10	49.79

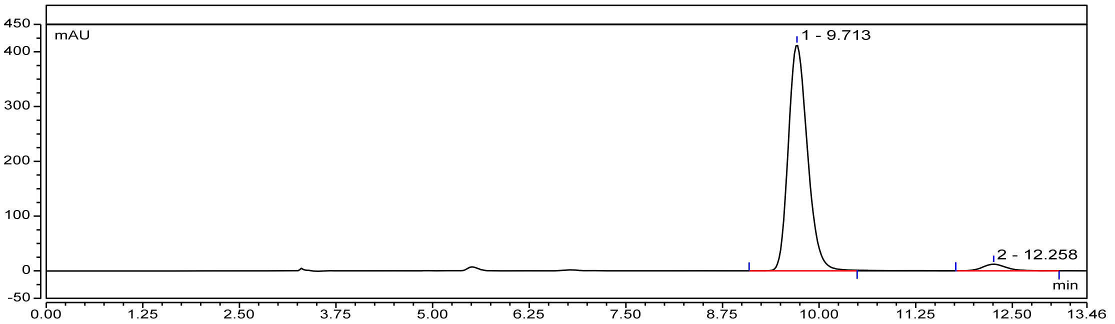

Entry	Retention Time	Area	Height	%Area
1	9.713	119.2228	412.78	96.52
2	12.258	4.3016	4.3016	3.48

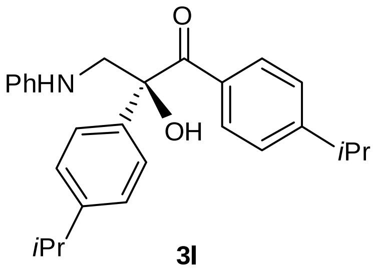

3l

Yellow oil; 33.3 mg, 83% yield; 91% ee; $[\alpha]_D^{22}$ +201.6 (*c* 2.0, $CHCl_3$); ^1H NMR (300 MHz, $CDCl_3$) δ 7.83 (d, *J* = 8.3 Hz, 2H), 7.46 (d, *J* = 8.2 Hz, 2H), 7.23 (d, *J* = 4.6 Hz, 2H), 7.13 (t, *J* = 8.8 Hz, 4H), 6.73 (t, *J* = 7.3 Hz, 1H), 6.65 (d, *J* = 7.8 Hz, 2H), 4.64 (s, 1H), 4.10 (d, *J* = 12.7 Hz, 1H), 3.54 (d, *J* = 12.7 Hz, 1H), 2.98 – 2.77 (m, 2H), 1.24 (d, 6H), 1.19(d, 6H); ^{13}C NMR (75 MHz, $CDCl_3$) δ 200.4, 154.6, 149.1, 148.4, 137.7, 132.1, 131.0, 129.4, 127.3, 126.5, 125.3, 119.0, 114.5, 53.5, 34.4, 34.0, 24.1, 23.8, 23.8; HRMS (ESI) m/z 402.2427 ($M+H^+$), calc. for $C_{27}H_{32}N_1O_2$ 402.2433.

The ee was determined by HPLC analysis: CHIRALPAK IC (4.6 mm i.d. x 250 mm); Hexane/2-propanol = 95/5; flow rate 1.0 mL/min; 25 ^oC; 254 nm; retention time: 8.5 min (major) and 10.5 min (minor).

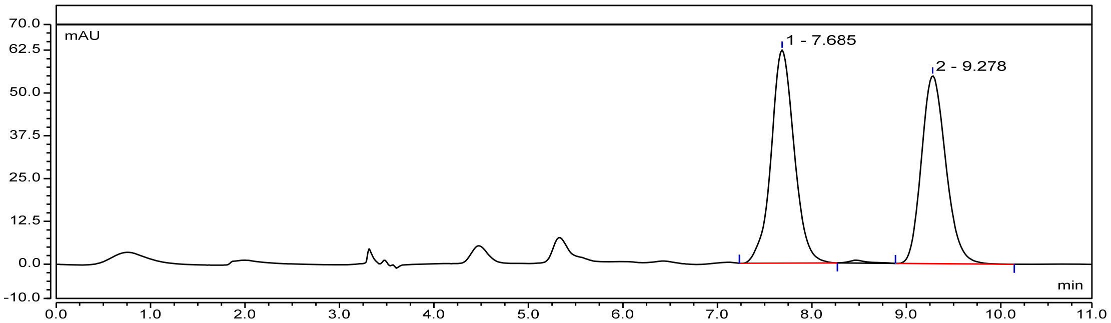

Entry	Retention Time	Area	Height	%Area
1	7.685	17.6257	62.24	51.75
2	9.278	16.4313	54.85	48.25

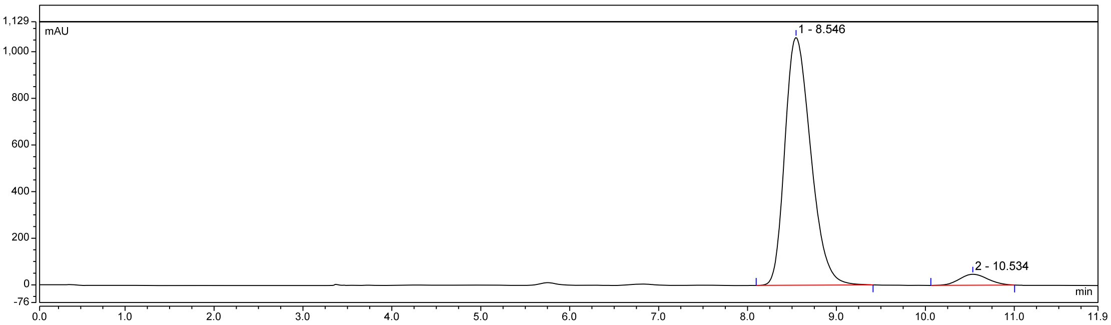

Entry	Retention Time	Area	Height	%Area
1	8.546	358.6272	1062.45	95.36
2	10.534	17.4492	47.06	4.64

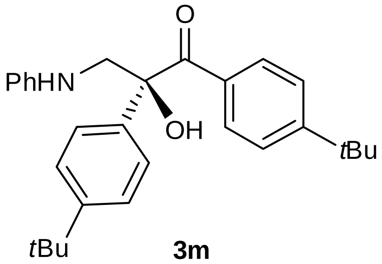

3m

Yellow solid; Mp 57.7 − 58.5 ^{o}C; 36.5 mg, 85% yield; 90% ee; $[\alpha]_D^{22}$ +89.6 (*c* 2.0, $CHCl_3$); ^{1}H NMR (300 MHz, $CDCl_3$) δ 7.85 (d, *J* = 8.5 Hz, 1H), 7.49 (d, *J* = 8.5 Hz, 1H), 7.42 (d, *J* = 8.5 Hz, 1H), 7.34 (d, *J* = 8.5 Hz, 1H), 7.15 (t, *J* = 7.9 Hz, 1H), 6.75 (t, *J* = 7.3 Hz, 0H), 6.67 (d, *J* = 7.9 Hz, 1H), 4.66 (s, 0H), 4.12 (d, *J* = 12.7 Hz, 1H), 3.58 (d, *J* = 12.7 Hz, 1H), 1.33 (s, 3H), 1.28 (s, 3H); ^{13}C NMR (75 MHz, $CDCl_3$) δ 200.5, 156.9, 151.4, 148.4, 137.3, 131.7), 130.7, 129.4, 126.2, 125.4, 125., 119.0, 114.6, 82.1, 53.5, 35.3, 34.8, 31.5, 31.2; HRMS (ESI) m/z 430.2754 (M+H$^+$), calc. for $C_{29}H_{36}N_1O_2$ 430.2746.

The ee was determined by HPLC analysis: CHIRALPAK IC (4.6 mm i.d. x 250 mm); Hexane/2-propanol = 95/5; flow rate 1.0 mL/min; 25 ^{o}C; 254 nm; retention time: 7.1 min (major) and 9.0 min (minor).

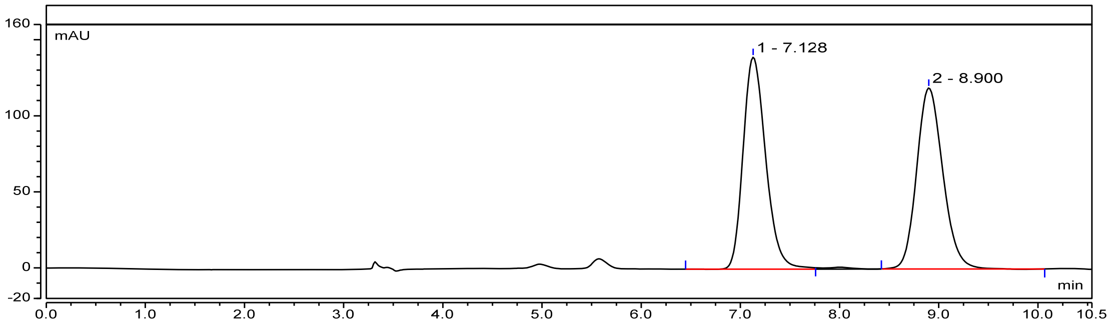

Entry	Retention Time	Area	Height	%Area
1	7.128	36.7559	139.24	50.26
2	8.900	36.3705	118.97	49.74

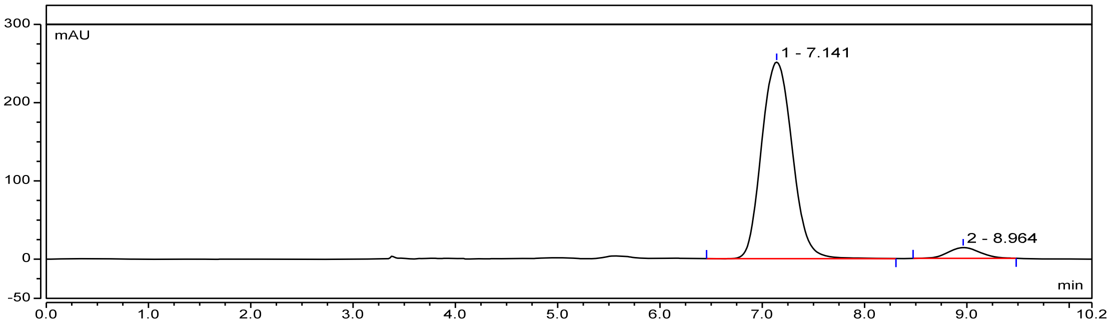

Entry	Retention Time	Area	Height	%Area
1	7.141	86.6588	250.77	94.91
2	8.964	4.6521	13.34	5.09

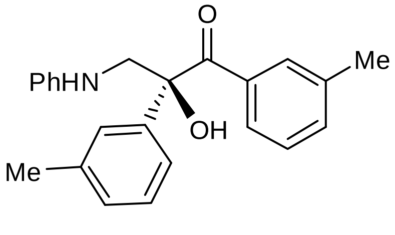

3n

Yellow oil; 28.0 mg, 81% yield; 93% ee; $[\alpha]_D^{22}$ +124.8 (*c* 2.0, $CHCl_3$); ^1H NMR (300 MHz, $CDCl_3$) δ 7.59 (s, 1H), 7.51 (d, *J* = 7.7 Hz, 1H), 7.19 (m, 3H), 7.12 (s, 1H), 7.04 (t, *J* = 7.8 Hz, 4H), 6.64 (t, *J* = 7.3 Hz, 1H), 6.57 (d, *J* = 7.8 Hz, 2H), 4.50 (s, 1H), 4.01 (d, *J* = 12.7 Hz, 1H), 3.40 (d, *J* = 12.8 Hz, 1H), 2.24 (s, 3H), 2.18 (s, 3H); ^{13}C NMR (75 MHz, $CDCl_3$) δ 201.0, 148.2, 140.1, 138.9, 138.0, 134.4, 133.8, 130.8, 129.4, 129.2, 129.0, 127.8, 127.8, 125.8, 122.2, 119.1, 114.6, 82.3, 53.6, 21.7, 21.5; HRMS (ESI) m/z 346.1810 (M+H$^+$), calc. for $C_{23}H_{24}N_1O_2$ 318.1494.

The ee was determined by HPLC analysis: CHIRALPAK IC (4.6 mm i.d. x 250 mm); Hexane/2-propanol = 95/5; flow rate 1.0 mL/min; 25 ^oC; 254 nm; retention time: 7.4 min (major) and 8.9 min (minor).

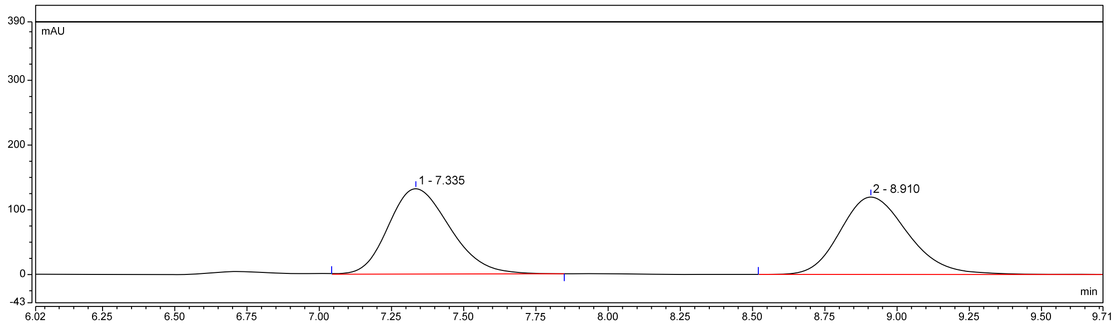

Entry	Retention Time	Area	Height	%Area
1	7.335	32.5326	131.69	50.03
2	8.910	32.4971	119.48	49.97

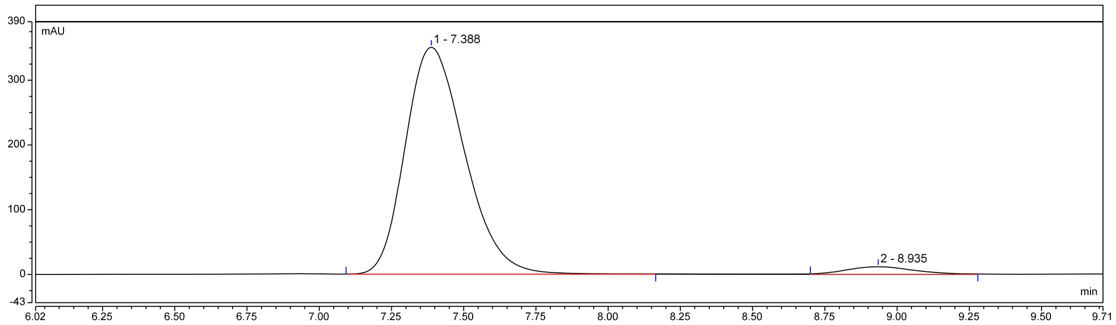

Entry	Retention Time	Area	Height	%Area
1	7.388	83.5608	350.26	96.40
2	8.935	3.1171	11.64	3.60

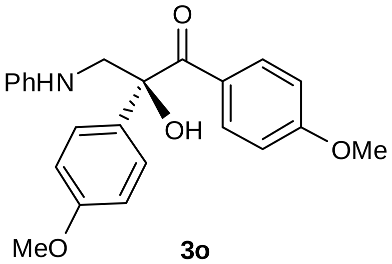

3o

Yellow solid; Mp 77.6 − 78.9 °C; 31.3 mg, 83% yield; 88% ee; $[\alpha]_D^{22}$ +21.9 (*c* 1.0, $CHCl_3$); ^{1}H NMR (300 MHz, $CDCl_3$) δ 7.92 (d, *J* = 8.9 Hz, 2H), 7.46 (d, *J* = 8.8 Hz, 2H), 7.16 (t, *J* = 7.8 Hz, 2H), 6.93 (d, *J* = 8.8 Hz, 2H), 6.77 (m, 3H), 6.69 (d, *J* = 7.9 Hz, 2H), 4.69 (s, 1H), 4.07 (d, *J* = 12.6 Hz, 1H), 3.80 (s, 3H), 3.79(s, 3H), 3.53 (d, *J* = 12.6 Hz, 1H); ^{13}C NMR (75 MHz, $CDCl_3$) δ 199.0, 163.4, 159.5, 148.3, 133.1, 132.7, 129.4, 126.9, 126.5, 118.9, 114.5, 114.4, 113.5, 81.5, 55.5, 55.4, 53.6; HRMS (ESI) m/z 378.1700 (M+H$^+$), calc. for $C_{23}H_{24}N_1O_4$ 378.1705.

The ee was determined by HPLC analysis: CHIRALPAK IC (4.6 mm i.d. x 250 mm); Hexane/2-propanol = 80/20; flow rate 1.0 mL/min; 25 °C; 254 nm; retention time: 10.5 min (major) and 12.4 min (minor).

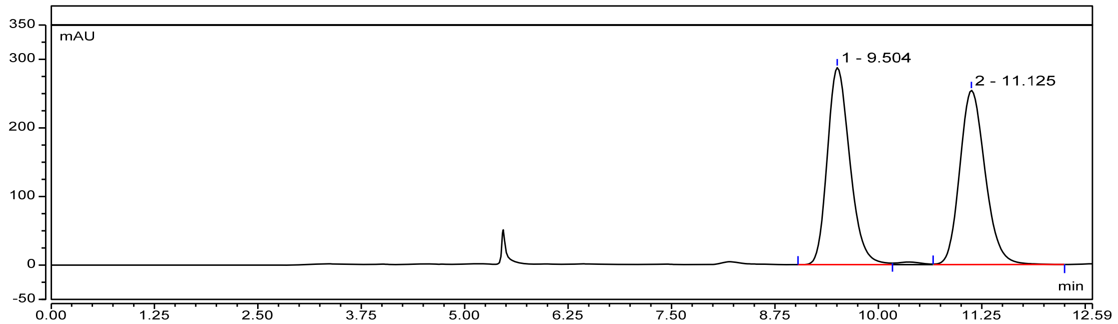

Entry	Retention Time	Area	Height	%Area
1	9.504	90.7926	287.30	50.12
2	11.125	90.3408	254.01	49.88

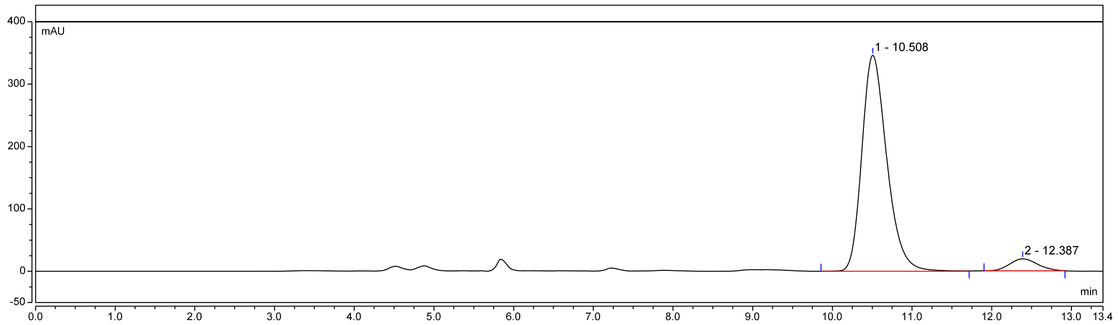

Entry	Retention Time	Area	Height	%Area
1	10.508	125.8121	346.24	94.20
2	12.387	7.7515	19.15	5.80

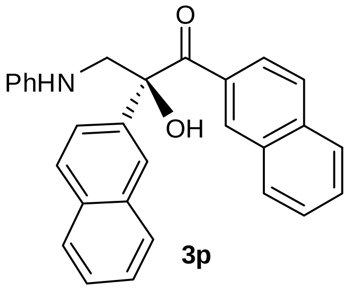

3p

Yellow solid; Mp 52.8 − 53.6 °C; 25.5 mg, 61% yield; 91% ee; $[\alpha]_D^{22}$ +47.3 (*c* 2.0, $CHCl_3$); ^{1}H NMR (300 MHz, $CDCl_3$) δ 8.55 (s, 1H), 8.14 (s, 1H), 7.97 (d, *J* = 8.7 Hz, 1H), 7.93 – 7.81 (m, 3H), 7.80 – 7.66 (m, 4H), 7.57 – 7.40 (m, 4H), 7.18 (t, *J* = 7.8 Hz, 2H), 6.78 (m, 3H), 4.80 (s, 1H), 4.31 (d, *J* = 12.9 Hz, 1H), 3.63 (d, *J* = 12.8 Hz, 1H); ^{13}C NMR (75 MHz, $CDCl_3$) δ 200.4, 148.3, 137.8, 135.4, 133.5, 133.2, 133.0, 132.2, 131.6, 123.0, 129.5, 129.1, 128.7, 128.4, 127.9, 127.8, 127.7, 126.6, 126.5, 125.9, 124.3, 122.8, 119.3, 114.68 (s, 6H), 82.7, 54.1; HRMS (ESI) m/z 418.1810 (M+H$^+$), calc. for $C_{29}H_{24}N_1O_2$ 418.1807.

The ee was determined by HPLC analysis: CHIRALPAK IC (4.6 mm i.d. x 250 mm); Hexane/2-propanol = 95/5; flow rate 1.0 mL/min; 25 °C; 254 nm; retention time: 10.7 min (major) and 13.9 min (minor).

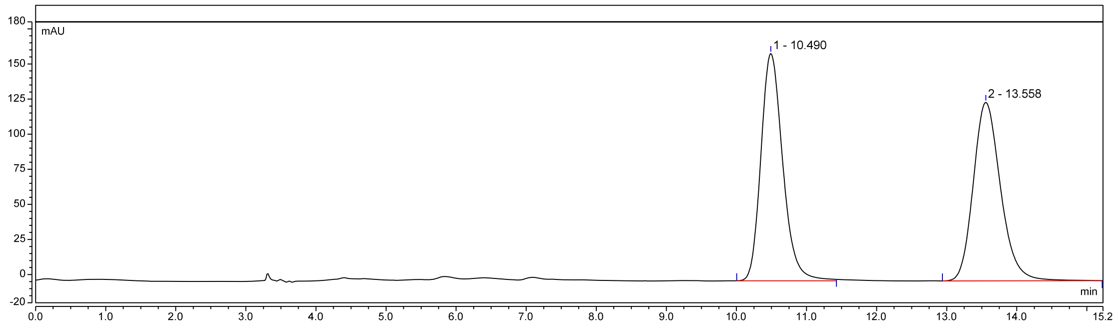

Entry	Retention Time	Area	Height	%Area
1	10.490	58.5250	161.89	50.54
2	13.558	57.2725	127.10	49.46

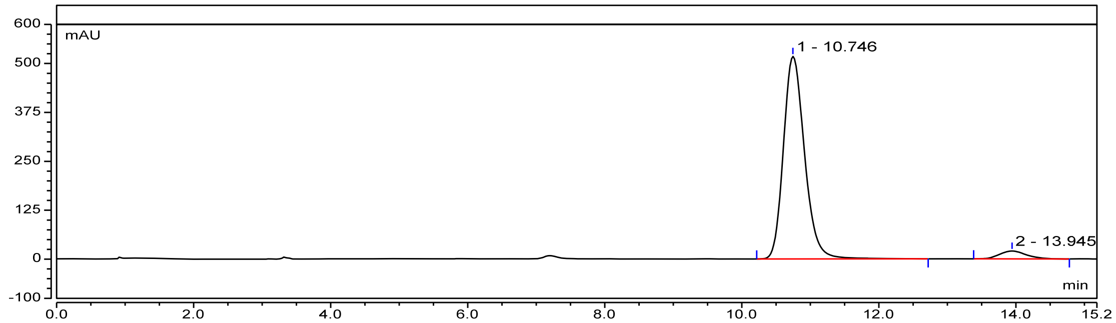

Entry	Retention Time	Area	Height	%Area
1	10.746	185.0326	517.80	95.33
2	13.945	9.0613	20.07	4.67

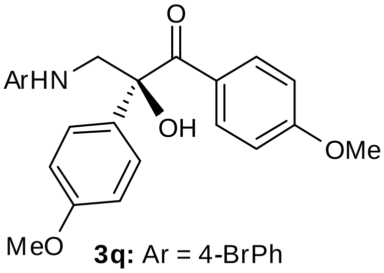

3q: Ar = 4-BrPh

Yellow solid; Mp 108.2 – 109.5 °C; 40.6 mg, 89% yield; 97% ee; $[\alpha]_D^{22}$ +53.4 (*c* 2.0, $CHCl_3$); ^{1}H NMR (300 MHz, $CDCl_3$) δ 7.95 – 7.78 (m, 2H), 7.49 – 7.36 (m, 2H), 7.21 (d, *J* = 8.7 Hz, 2H), 6.92 (d, *J* = 8.7 Hz, 2H), 6.78 (d, *J* = 8.9 Hz, 2H), 6.53 (d, *J* = 8.7 Hz, 2H), 4.64 (s, 1H), 4.15 (s, 1H), 4.01 (d, *J* = 12.7 Hz, 1H), 3.80 (s, 6H), 3.52 (d, *J* = 12.6 Hz, 1H); ^{13}C NMR (75 MHz, $CDCl_3$) δ 198.8, 163.4, 159.5, 147.3, 133.1, 132.4, 132.0, 126.7, 126.6, 115.9, 114.5, 113.6, 110.4, 81.4, 55.5, 55.4, 53.2; HRMS (ESI) m/z 458.0805 (M+H$^+$), calc. for $C_{23}H_{23}N_1O_4Br_1$ 458.0790.

The ee was determined by HPLC analysis: CHIRALPAK IC (4.6 mm i.d. x 250 mm); Hexane/2-propanol = 80/20; flow rate 1.0 mL/min; 25 °C; 254 nm; retention time: 8.3 min (major) and 9.9 min (minor).

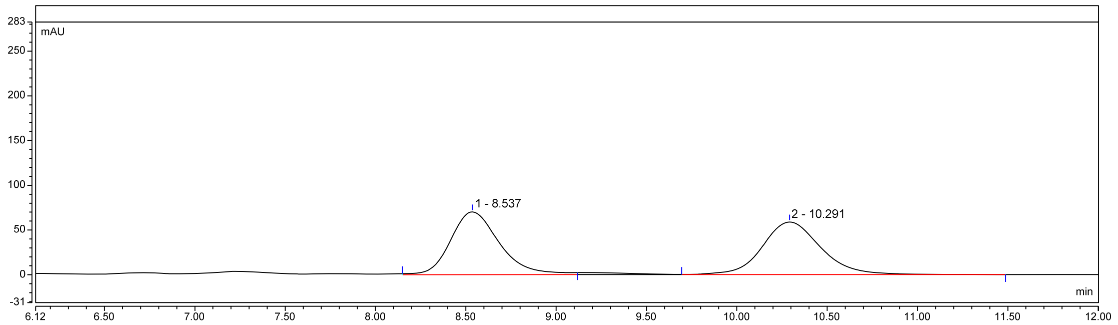

Entry	Retention Time	Area	Height	%Area
1	8.537	21.9589	70.10	49.83
2	10.291	22.1082	58.82	50.17

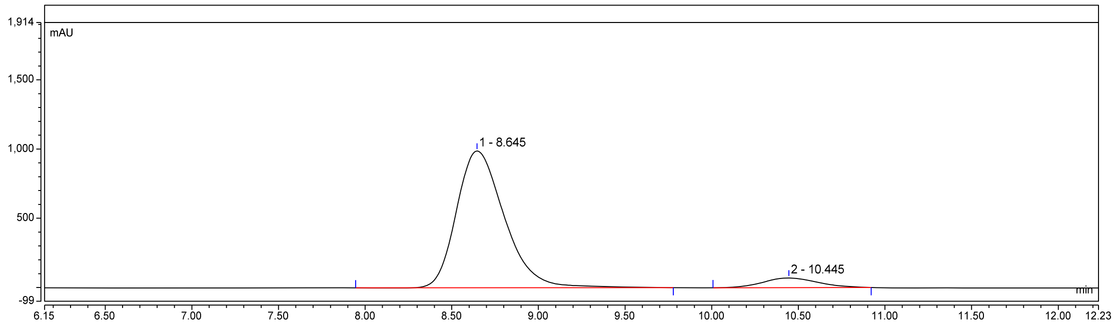

Entry	Retention Time	Area	Height	%Area
1	8.645	316.8719	987.56	92.56
2	10.445	25.4775	69.85	7.44

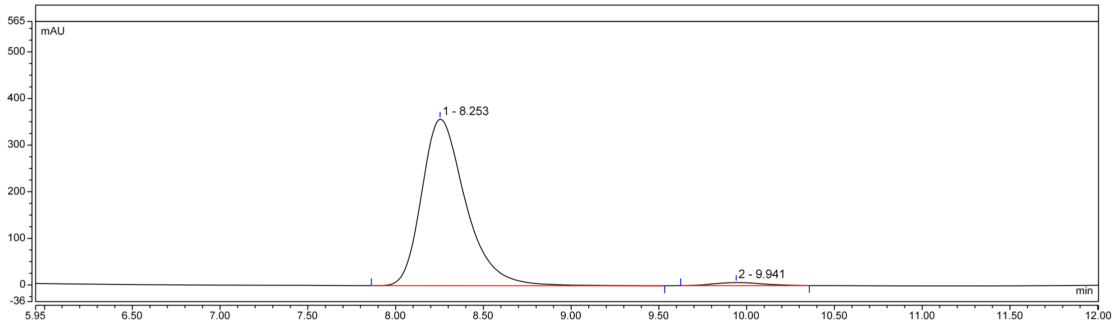

Entry	Retention Time	Area	Height	%Area
1	8.253	103.8814	357.14	98.48
2	9.941	1.6009	5.82	1.52

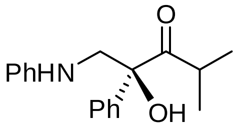

3r

Yellow oil; 18.42 mg, 65% yield; 78% ee; $[\alpha]_D^{22}$ –54.3 (*c* 0.1, $CHCl_3$); ^{1}H NMR (300 MHz, $CDCl_3$) δ 7.49 (m, 2H), 7.44 – 7.32 (m, 3H), 7.22 (t, *J* = 7.9 Hz, 2H), 6.80 (m, 3H), 4.86 (s, 1H), 4.16 (d, *J* = 12.2 Hz, 1H), 3.56 (d, *J* = 12.2 Hz, 1H), 3.08 (m, 6.7 Hz, 1H), 0.92 (d, *J* = 6.8 Hz, 3H), 0.81 (d, *J* = 6.7 Hz, 3H); ^{13}C NMR (75 MHz, $CDCl_3$) δ 215.6, 147.3, 129.5, 128.9, 128.6, 125.9, 119.6, 114.8, 82.3, 50.5, 34.4, 20.5, 19.7; HRMS (ESI) m/z 284.1638 ($M+H^+$), calc. for $C_{18}H_{22}NO_2$ 284.1645.

The ee was determined by HPLC analysis: CHIRALPAK IE (4.6 mm i.d. x 250 mm); Hexane/2-propanol = 98/2; flow rate 0.5 mL/min; 25 °C; 254 nm; retention time: 18.2 min (minor) and 26.4 min (major).

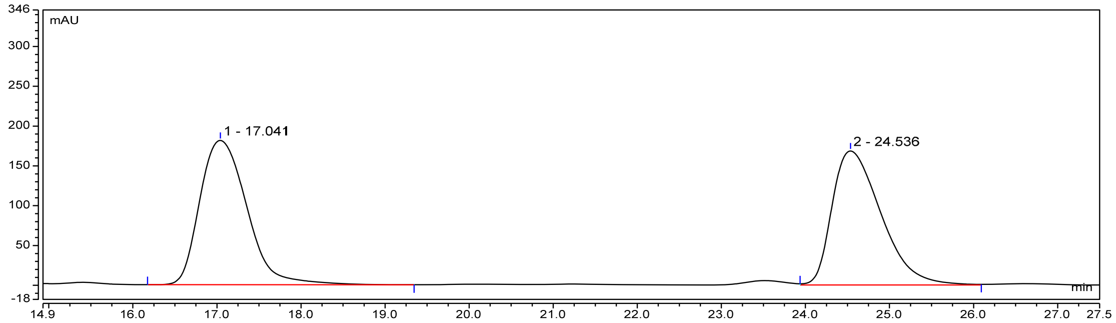

Entry	Retention Time	Area	Height	%Area
1	17.041	117.7428	181.29	50.92
2	24.536	113.5090	168.34	49.08

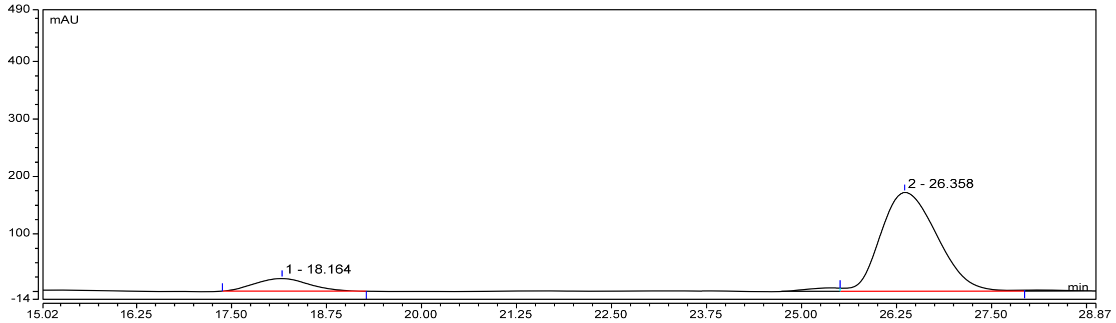

Entry	Retention Time	Area	Height	%Area
1	18.164	18.2070	22.13	10.98
2	26.354	147.5912	171.73	89.02

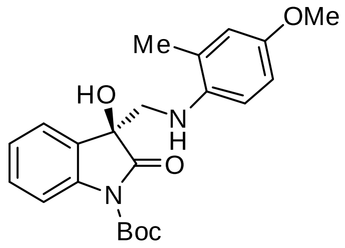

5a

Yellow oil; 36.3 mg, 91% yield; 91% ee; $[\alpha]_D^{22}$ +110.2 (*c* 2.0, $CHCl_3$); ^{1}H NMR (300 MHz, $CDCl_3$) δ 7.86 (d, *J* = 8.2 Hz, 1H), 7.49 (d, *J* = 7.3 Hz, 1H), 7.40 (t, *J* = 7.9 Hz, 1H), 7.22 (d, *J* = 7.4 Hz, 1H), 6.74 – 6.53 (m, 3H), 4.26 (s, 1H), 3.73 (s, 3H), 3.62 (d, *J* = 12.7 Hz, 1H), 3.34 (d, *J* = 12.6 Hz, 1H), 2.15 (s, 3H), 1.64 (s, 9H); ^{13}C NMR (75 MHz, $CDCl_3$) δ 176.7, 152.8, 148.8, 139.7, 139.2, 130.5, 127.5, 126.1, 125.6, 124.2, 116.92, 115.42, 112.9, 111.5, 84.9, 74.3, 55.7, 52.0, 28.1, 17.8; HRMS (ESI) m/z 399.1926 (M+H$^+$), calc. for $C_{22}H_{27}N_2O_5$ 399.1920.

The ee was determined by HPLC analysis: CHIRALPAK IC (4.6 mm i.d. x 250 mm); Hexane/2-propanol = 90/10; flow rate 1.0 mL/min; 25 °C; 254 nm; retention time: 21.0 min (major) and 30.9 min (minor).

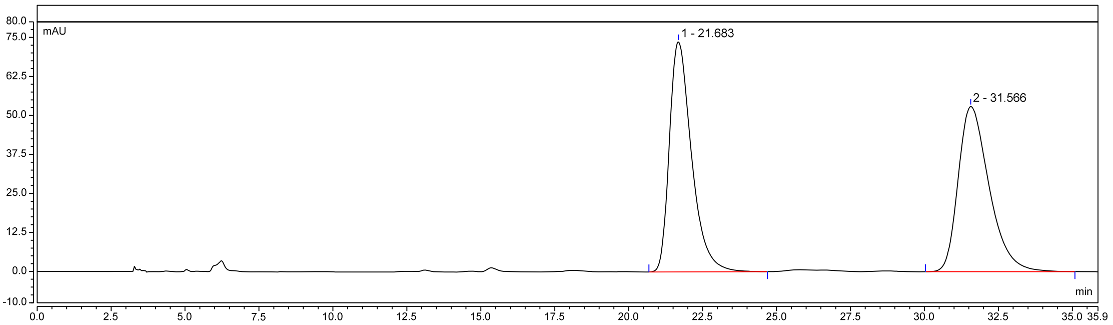

Entry	Retention Time	Area	Height	%Area
1	21.683	64.0677	73.70	49.94
2	31.566	64.2272	52.96	50.06

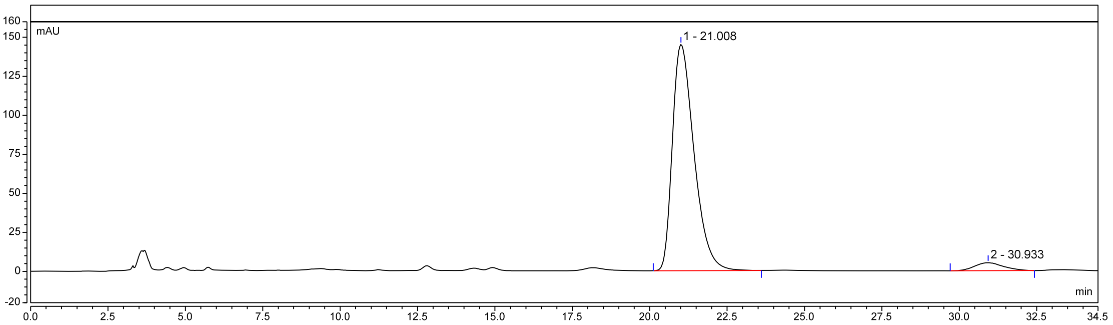

Entry	Retention Time	Area	Height	%Area
1	21.008	117.7823	144.94	95.53
2	30.933	5.5157	5.10	4.47

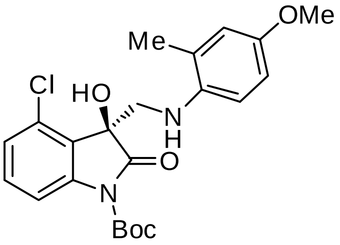

5b

Yellow oil; 34.6 mg, 80% yield; 85% ee; $[\alpha]_D^{22}$ –19.5 (*c* 1.0, $CHCl_3$); ^1H NMR (300 MHz, $CDCl_3$) δ 7.81 (d, *J* = 8.1 Hz, 1H), 7.32 (t, *J* = 8.2 Hz, 1H), 7.18 (d, *J* = 8.1 Hz, 1H), 6.74 – 6.63 (m, 3H), 4.13 (m, 2H), 3.83 (s, 1H), 3.73 (s, 3H), 3.52 (d, *J* = 12.9 Hz, 1H), 2.11 (s, 3H), 1.62 (s, 9H); ^{13}C NMR (75 MHz, $CDCl_3$) δ 175.7, 153.0, 148.6, 141.9, 139.1, 131.7, 131.5, 126.3, 126.1, 124.0, 116.9, 114.0, 113.5, 111.7, 85.4, 75.5, 55.7, 49.3, 28.1, 17.8; HRMS (ESI) m/z 433.1546 ($M+H^+$), calc. for $C_{22}H_{26}N_2O_5Cl_1$ 433.1530.

The ee was determined by HPLC analysis: CHIRALPAK IC (4.6 mm i.d. x 250 mm); Hexane/2-propanol = 90/10; flow rate 1.0 mL/min; 25 °C; 254 nm; retention time: 18.9 min (minor) and 21.0 min (major).

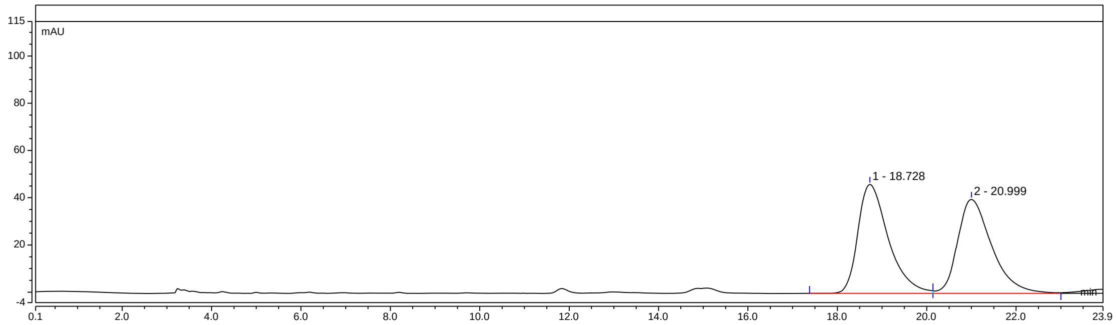

Entry	Retention Time	Area	Height	%Area
1	18.728	36.3839	46.09	50.90
2	20.999	35.0914	39.78	49.10

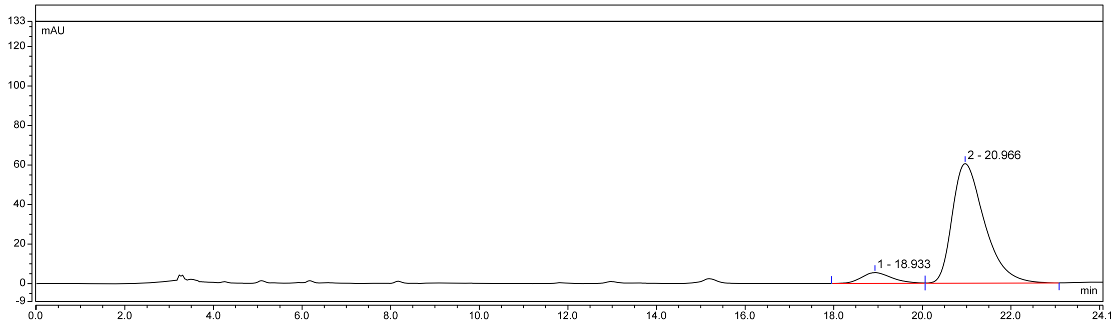

Entry	Retention Time	Area	Height	%Area
1	18.933	4.1145	5.36	7.46
2	20.966	51.0292	60.49	92.54

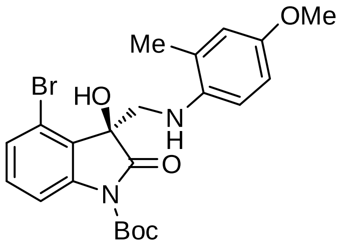

5c

Yellow oil; 36.8 mg, 77% yield; 85% ee; $[\alpha]_D^{22}$ –31.8 (*c* 2.0, $CHCl_3$); ^1H NMR (300 MHz, $CDCl_3$) δ 7.85 (d, *J* = 8.1 Hz, 1H), 7.35 (d, *J* = 8.1 Hz, 1H), 7.25 – 7.19 (m, 1H), 6.74 – 6.61 (m, 3H), 4.14 (d, *J* = 12.7 Hz, 1H), 4.08 (s, 1H), 3.77 (s, 1H), 3.72 (s, 3H), 3.52 (d, *J* = 12.9 Hz, 1H), 2.10 (s, 3H), 1.60 (s, 9H); ^{13}C NMR (75 MHz, $CDCl_3$) δ 175.7, 153.0, 148.5, 142.0, 139.0, 131.7, 129.5, 126.1, 125.5, 119.4, 116.9, 114.5, 113.6, 111.7, 85.4, 75.9, 55.7, 49.2, 28.1, 17.8; HRMS (ESI) m/z 477.1033 ($M+H^+$), calc. for $C_{22}H_{26}N_2O_5Br_1$ 477.1025.

The ee was determined by HPLC analysis: CHIRALPAK IC (4.6 mm i.d. x 250 mm); Hexane/2-propanol = 90/10; flow rate 1.0 mL/min; 25 ^oC; 254 nm; retention time: 20.0 min (minor) and 22.5 min (major).

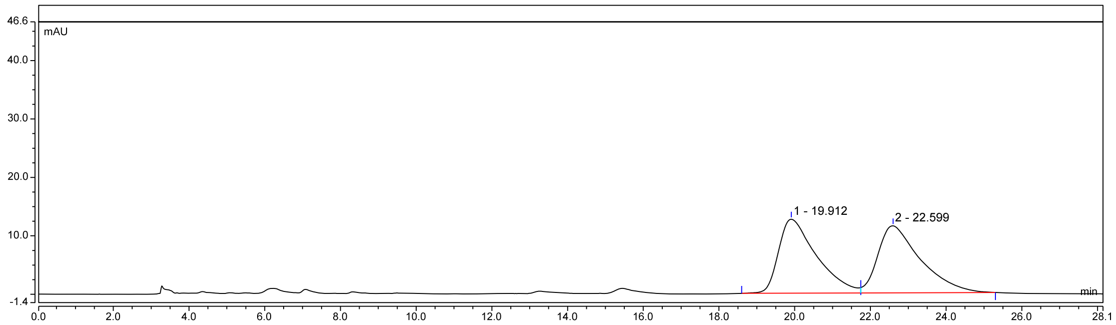

Entry	Retention Time	Area	Height	%Area
1	19.912	14.6965	12.67	49.56
2	22.599	14.9578	11.47	50.44

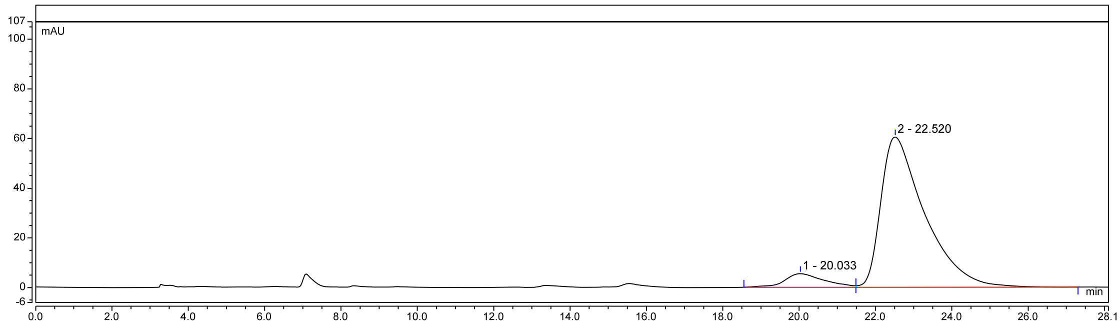

Entry	Retention Time	Area	Height	%Area
1	20.033	6.5428	5.48	7.53
2	22.520	80.3687	60.46	92.47

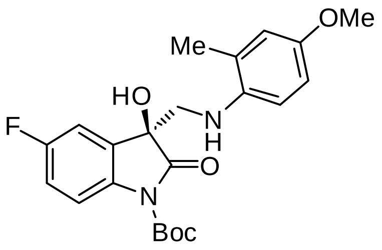

5d

Yellow oil; 36.6 mg, 88% yield; 92% ee; $[\alpha]_D^{22}$ +122.6 (*c* 1.0, $CHCl_3$); ^{1}H NMR (300 MHz, $CDCl_3$) δ 7.86 (m, 1H), 7.22 (m, 1H), 7.09 (m, 1H), 6.74 – 6.56 (m, 3H), 3.74 (s, 3H), 3.57 (d, *J* = 12.8 Hz, 1H), 3.35 (d, *J* = 12.7 Hz, 1H), 2.18 (s, 3H), 1.63 (s, 9H); ^{13}C NMR (75 MHz, $CDCl_3$) δ 176.7, 152.8, 148.8, 139.7, 139.2, 130.5, 127.5, 126.1, 125.2, 124.7, 116.9, 115.4, 112.9, 111.5, 84.9, 74.3, 55.7, 52.0, 28.1, 17.8; HRMS (ESI) m/z 417.1840 (M+H$^+$), calc. for $C_{22}H_{26}N_2O_5F_1$ 417.1826.

The ee was determined by HPLC analysis: CHIRALPAK IC (4.6 mm i.d. x 250 mm); Hexane/2-propanol = 90/10; flow rate 1.0 mL/min; 25 ^{o}C; 254 nm; retention time: 15.6 min (major) and 25.3 min (minor).

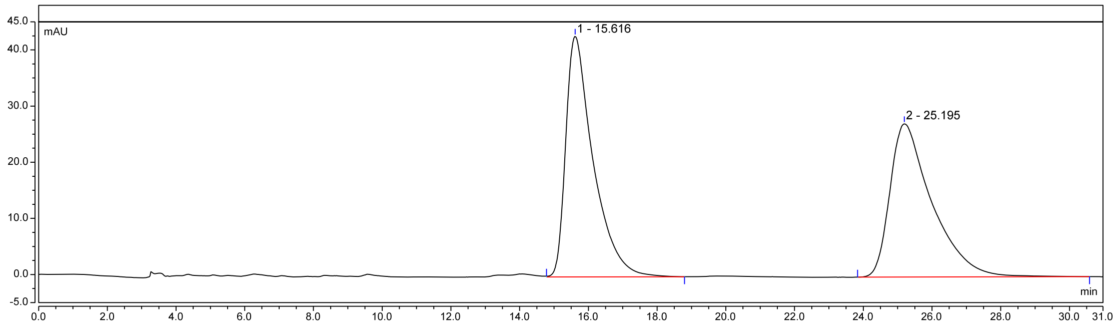

Entry	Retention Time	Area	Height	%Area
1	15.616	39.5710	42.84	50.55
2	25.195	38.7168	27.29	49.45

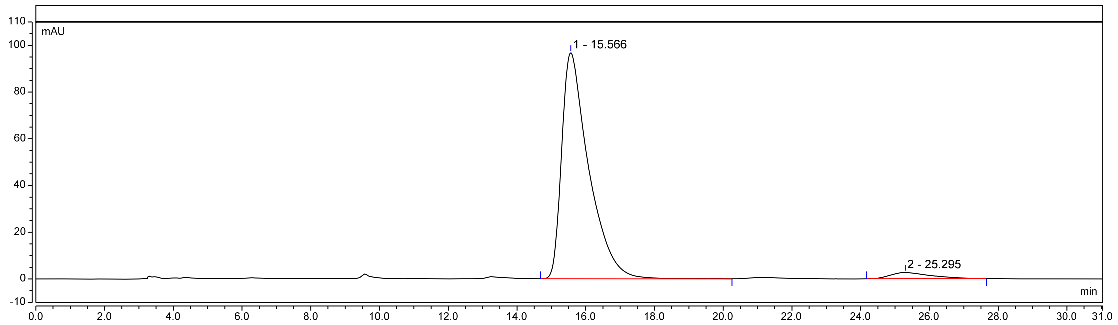

Entry	Retention Time	Area	Height	%Area
1	15.566	88.2816	96.75	95.98
2	25.295	3.6957	2.69	4.02

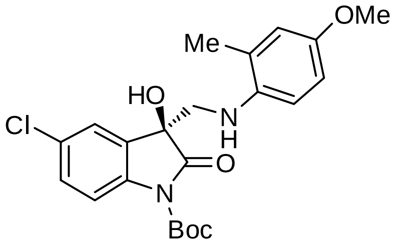

5e

Yellow oil; 38.1 mg, 88% yield; 90% ee; $[\alpha]_D^{22}$ +20.3 (*c* 1.0, $CHCl_3$); ^{1}H NMR (300 MHz, $CDCl_3$) δ 7.82 (d, *J* = 8.7 Hz, 1H), 7.46 (d, *J* = 2.0 Hz, 1H), 7.37 (m, 1H), 6.77 – 6.58 (m, 3H), 4.21 (s, 1H), 3.96 (s, 1H), 3.74 (s, 3H), 3.58 (d, *J* = 13.0 Hz, 1H), 3.34 (d, *J* = 12.8 Hz, 1H), 2.19 (s, 3H), 1.63 (s, 9H); ^{13}C NMR (75 MHz, $CDCl_3$) δ 176.0, 153.2, 148.7, 139.1, 138.3, 130.8, 130.6, 129.3, 126.4, 124.6, 117.1, 116.9, 113.7, 111.7, 85.4, 74.2, 55.8, 52.2, 28.2, 17.9.

The ee was determined by HPLC analysis: CHIRALPAK IC (4.6 mm i.d. x 250 mm); Hexane/2-propanol = 90/10; flow rate 1.0 mL/min; 25 ^{o}C; 254 nm; retention time: 14.5 min (major) and 36.7 min (minor) ; HRMS (ESI) m/z 433.1543 (M+H$^+$), calc. for $C_{22}H_{26}N_2O_5Cl_1$ 433.1530.

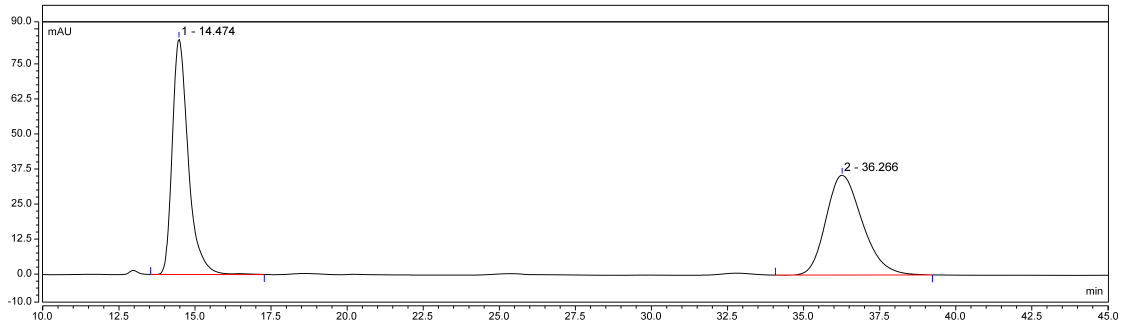

Entry	Retention Time	Area	Height	%Area
1	14.474	51.2996	83.88	51.00
2	36.266	49.2937	35.56	49.00

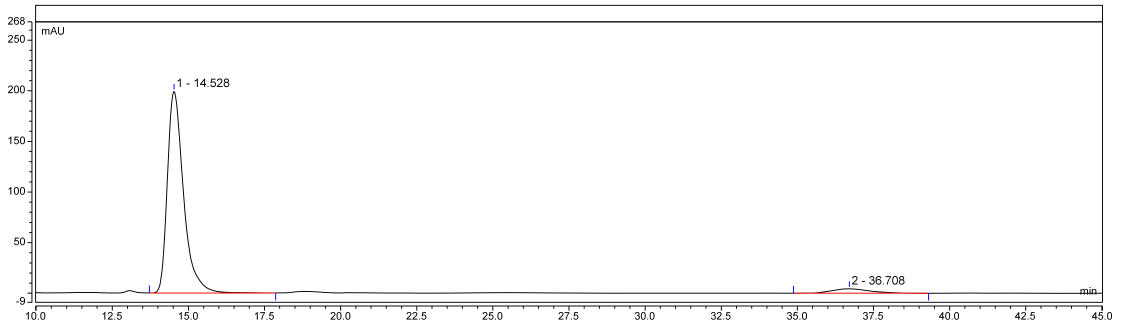

Entry	Retention Time	Area	Height	%Area
1	14.528	122.5667	199.26	95.14
2	36.708	6.2564	4.39	4.86

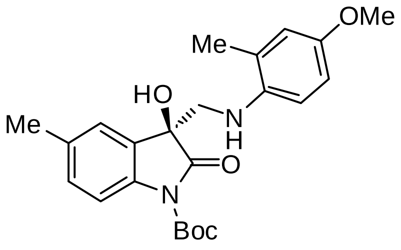

5f

Yellow solid; Mp 46.0 − 47.5 °C; 42.0 mg, 88% yield; 94% ee; $[\alpha]_D^{22}$ –9.7 (*c* 1.0, $CHCl_3$); ^1H NMR (300 MHz, $CDCl_3$) δ 7.76 (d, *J* = 8.7 Hz, 1H), 7.60 (d, *J* = 1.7 Hz, 1H), 7.52 (m, 1H), 6.73 – 6.59 (m, 3H), 3.74 (s, 3H), 3.58 (d, *J* = 12.8 Hz, 1H), 3.34 (d, *J* = 12.8 Hz, 1H), 2.19 (s, 3H), 1.62 (s, 9H); ^{13}C NMR (75 MHz, $CDCl_3$) δ 175.9, 153.2, 148.7, 139.0, 138.8, 133.5, 129.6, 127.5, 126.4, 118.2, 117.2, 117.1, 113.2, 111.6, 85.4, 74.1, 55.8, 52.1, 28.1, 17.9; HRMS (ESI) m/z 477.1031 ($M+H^+$), calc. for $C_{22}H_{26}N_2O_5Br_1$ 477.1025.

The ee was determined by HPLC analysis: CHIRALPAK IC (4.6 mm i.d. x 250 mm); Hexane/2-propanol = 90/10; flow rate 1.0 mL/min; 25 °C; 254 nm; retention time: 14.8 min (major) and 45.9 min (minor).

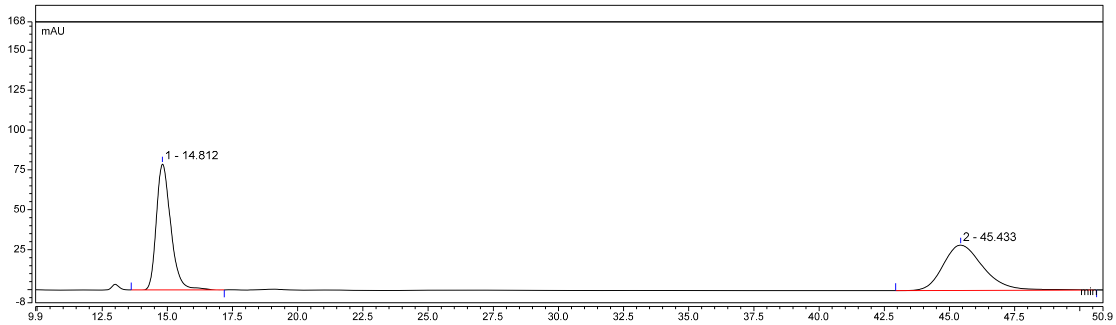

Entry	Retention Time	Area	Height	%Area
1	14.812	50.6193	78.92	49.83
2	45.433	50.9635	28.35	50.17

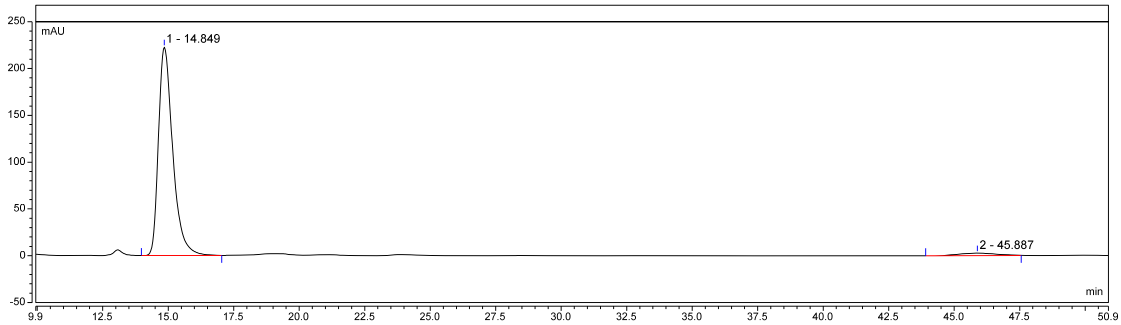

Entry	Retention Time	Area	Height	%Area
1	14.849	140.4197	222.58	97.07
2	45.887	4.2318	2.64	2.93

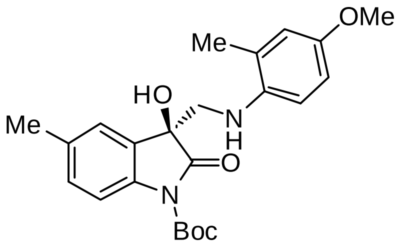

5g

Yellow solid; Mp 49.6 − 50.8 °C; 37.5 mg, 91% yield; 90% ee; $[\alpha]_D^{22}$ +51.3 (*c* 2.0, $CHCl_3$); ^{1}H NMR (300 MHz, $CDCl_3$) δ 7.73 (d, *J* = 8.3 Hz, 1H), 7.29 (s, 1H), 7.20 (d, *J* = 8.3 Hz, 1H), 6.73 – 6.59 (m, 3H), 4.19 (s, 1H), 4.00 (s, 1H), 3.74 (s, 3H), 3.62 (d, *J* = 10.9 Hz, 1H), 3.31 (d, *J* = 12.6 Hz, 1H), 2.37 (s, 3H), 2.17 (s, 3H), 1.63 (s, 9H); ^{13}C NMR (75 MHz, $CDCl_3$) δ 176.9, 153.0, 148.9, 139.4, 137.4, 135.0, 131.0, 127.4, 126.2, 124.7, 117.0, 115.3, 113.0, 111.6, 84.8, 74.2, 55.8, 52.1, 28.2, 21.1, 17.9; HRMS (ESI) m/z 413.2074 (M+H$^+$), calc. for $C_{23}H_{29}N_2O_5$ 413.2076.

The ee was determined by HPLC analysis: CHIRALPAK IC (4.6 mm i.d. x 250 mm); Hexane/2-propanol = 90/10; flow rate 1.0 mL/min; 25 °C; 254 nm; retention time: 26.1 min (major) and 39.9 min (minor).

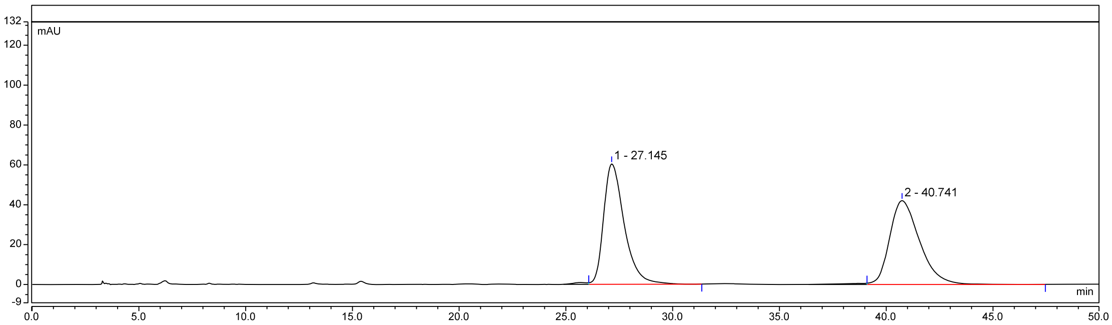

Entry	Retention Time	Area	Height	%Area
1	27.145	67.3818	60.31	49.72
2	40.741	68.1531	42.04	50.28

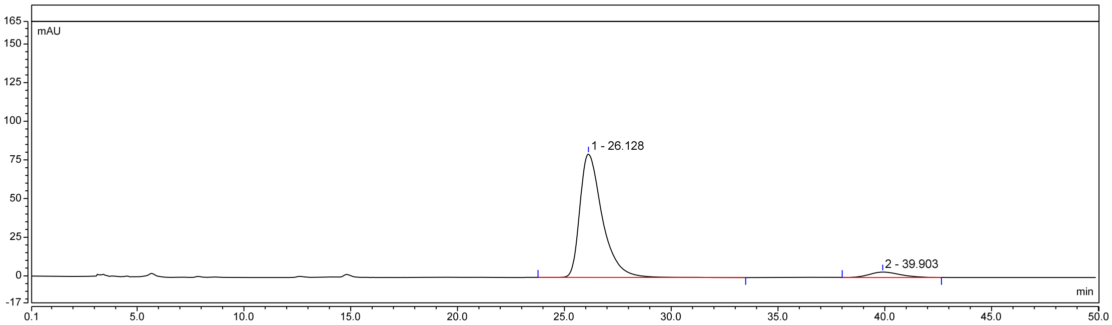

Entry	Retention Time	Area	Height	%Area
1	26.128	96.9323	79.82	94.90
2	39.903	5.2086	3.33	5.10

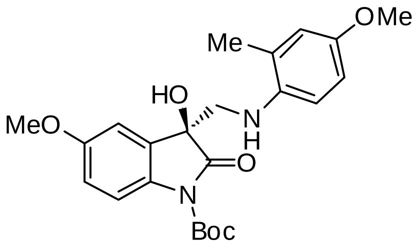

5h

Yellow oil; 38.6 mg, 90% yield; 85% ee; $[\alpha]_D^{22}$ +59.4 (*c* 2.0, $CHCl_3$); ^{1}H NMR (300 MHz, $CDCl_3$) δ 7.77 (d, *J* = 8.9 Hz, 1H), 7.04 (d, *J* = 2.5 Hz, 1H), 6.91 (dd, *J* = 8.9, 2.6 Hz, 1H), 6.71 – 6.57 (m, 3H), 4.21 (s, 1H), 3.96 (s, 1H), 3.81 (s, 3H), 3.73 (s, 3H), 3.59 (d, *J* = 12.7 Hz, 1H), 3.33 (d, *J* = 12.6 Hz, 1H), 2.16 (s, 3H), 1.62 (s, 9H); ^{13}C NMR (75 MHz, $CDCl_3$) δ 176.7, 157.4, 152.9, 148.9, 139.3, 132.9, 128.7, 126.1, 117.0, 116.6, 115.7, 112.9, 111.6, 109.9, 84.8, 74.6, 55.8, 55.7, 52.2, 28.2, 17.8; HRMS (ESI) m/z 429.2028 (M+H$^+$), calc. for $C_{23}H_{29}N_2O_6$ 429.2026.

The ee was determined by HPLC analysis: CHIRALPAK IC (4.6 mm i.d. x 250 mm); Hexane/2-propanol = 90/10; flow rate 1.0 mL/min; 25 ^{o}C; 254 nm; retention time: 15.3 min (major) and 29.1 min (minor).

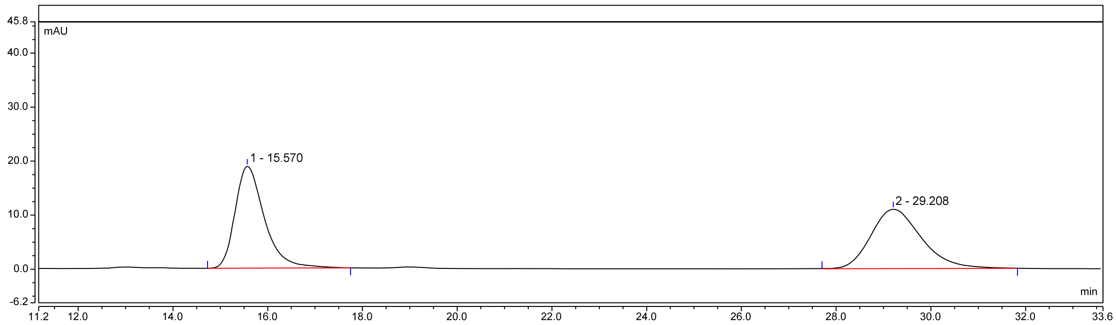

Entry	Retention Time	Area	Height	%Area
1	15.570	13.5298	18.84	49.96
2	29.208	13.5524	10.97	50.04

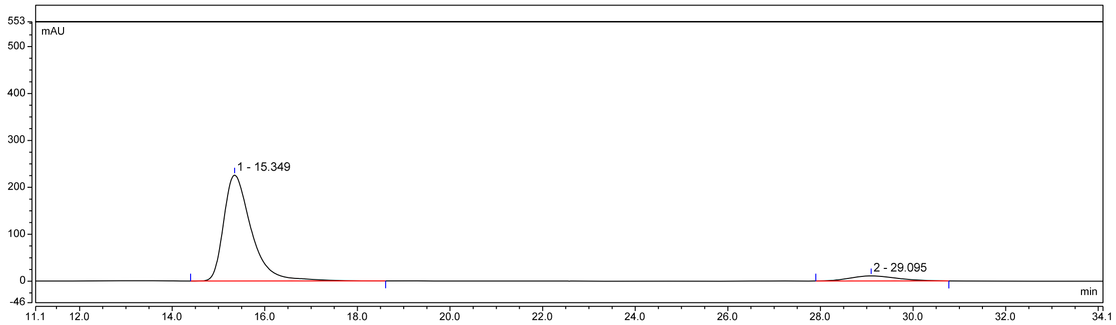

Entry	Retention Time	Area	Height	%Area
1	15.349	155.8772	225.67	92.44
2	29.095	12.7394	10.81	7.56

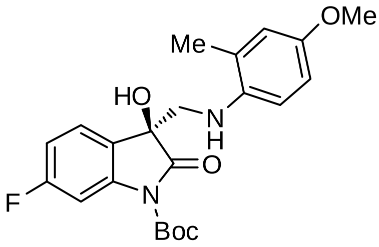

5i

Yellow oil; 30.8 mg, 74% yield; 93% ee; $[\alpha]_D^{22}$ +164.6 (*c* 1.0, $CHCl_3$); ^{1}H NMR (300 MHz, $CDCl_3$) δ 7.65 (dd, *J* = 10.2, 2.3 Hz, 1H), 7.44 (dd, *J* = 8.3, 5.6 Hz, 1H), 6.92 (m, 1H), 6.70 – 6.57 (m, 3H), 3.74 (s, 3H), 3.58 (d, *J* = 12.8 Hz, 1H), 3.34 (d, *J* = 12.8 Hz, 1H), 2.17 (s, 3H), 1.63 (s, 9H); ^{13}C NMR (75 MHz, $CDCl_3$) δ 176.5, 165.7, 162.5, 153.3, 148.8, 141.4, 141.2, 139.3, 126.4, 125.7, 125.6, 123.3,123.2, 117.2, 113.3, 112.2, 111.9, 111.8, 104.8, 104.4, 85.6, 74.2, 55.9, 52.4, 28.3, 18.0; HRMS (ESI) m/z 417.1832 (M+H$^+$), calc. for $C_{22}H_{26}N_2O_5F_1$ 417.1826.

The ee was determined by HPLC analysis: CHIRALPAK IC (4.6 mm i.d. x 250 mm); Hexane/2-propanol = 95/5; flow rate 1.0 mL/min; 25 ^{o}C; 254 nm; retention time: 29.1 min (major) and 54.1 min (minor).

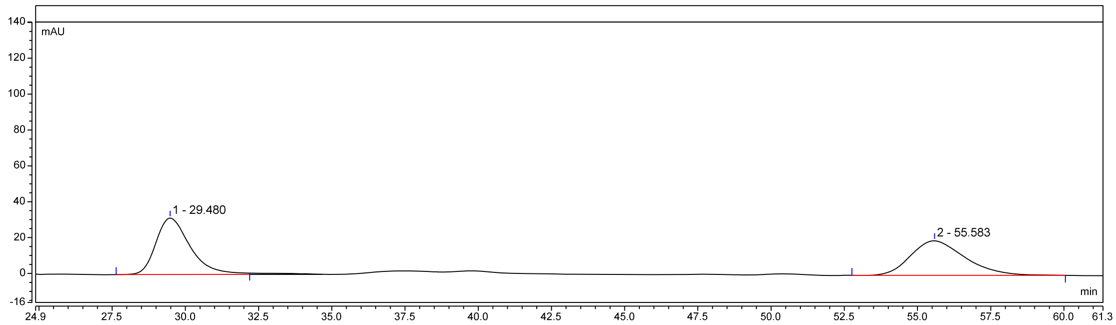

Entry	Retention Time	Area	Height	%Area
1	29.480	42.6606	31.50	50.76
2	55.583	41.3819	19.29	49.24

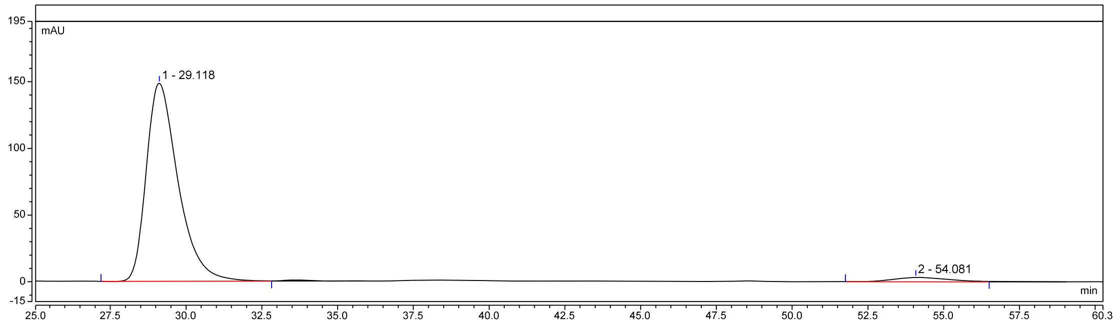

Entry	Retention Time	Area	Height	%Area
1	29.118	180.5570	148.50	96.45
2	54.081	6.6459	3.21	3.55

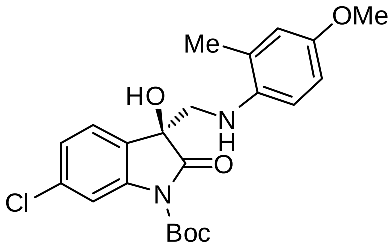

5j

Yellow oil; 32.9 mg, 76% yield; 93% ee; $[\alpha]_D^{22}$ +12.2 (*c* 1.0, $CHCl_3$); ^1H NMR (300 MHz, $CDCl_3$) δ 7.94 (d, *J* = 1.7 Hz, 1H), 7.41 (d, *J* = 8.0 Hz, 1H), 7.21 (dd, *J* = 8.0, 1.7 Hz, 1H), 6.70 – 6.57 (m, 3H), 3.73 (s, 3H), 3.57 (d, *J* = 12.8 Hz, 1H), 3.34 (d, *J* = 12.8 Hz, 1H), 2.17 (s, 3H), 1.63 (s, 9H); ^{13}C NMR (75 MHz, $CDCl_3$) δ 176.2, 153.1. 148.7, 140.7, 139.1, 136.4, 126.3, 126.0, 125.3, 125.2, 117.0, 116.3, 113.2, 111.7, 85.5, 74.1, 55.8, 52.2, 28.1, 17.9; HRMS (ESI) m/z 433.1517 ($M+H^+$), calc. for $C_{22}H_{26}N_2O_5Cl_1$ 433.1530.

The ee was determined by HPLC analysis: CHIRALPAK IC (4.6 mm i.d. x 250 mm); Hexane/2-propanol = 90/10; flow rate 1.0 mL/min; 25 °C; 254 nm; retention time: 13.7 min (major) and 23.4 min (minor).

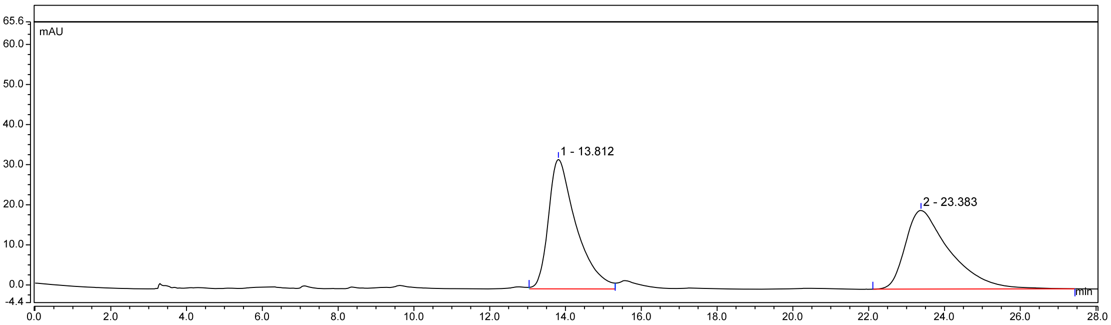

Entry	Retention Time	Area	Height	%Area
1	13.812	27.4522	32.26	50.67
2	23.383	26.7208	19.62	49.33

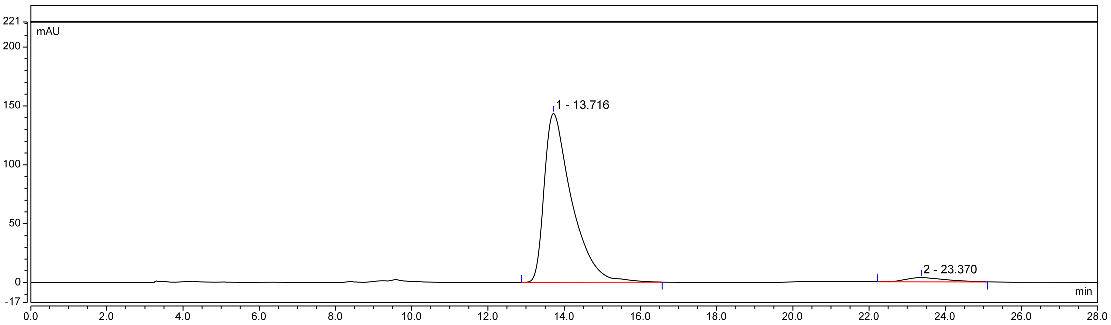

Entry	Retention Time	Area	Height	%Area
1	13.716	119.4245	143.33	96.48
2	23.370	4.3589	3.62	3.52

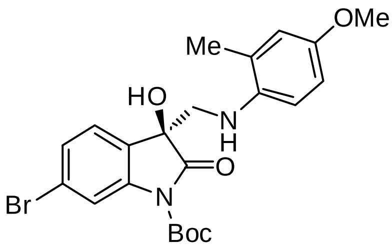

5k

Yellow oil; 37.2 mg, 78% yield; 90% ee; $[\alpha]_D^{22}$ +124.4 (*c* 1.0, $CHCl_3$); ^{1}H NMR (300 MHz, $CDCl_3$) δ 8.09 (d, *J* = 0.8 Hz, 1H), 7.40 – 7.32 (m, 2H), 6.70 – 6.56 (m, 3H), 3.73 (s, 3H), 3.56 (d, *J* = 12.8 Hz, 1H), 3.33 (d, *J* = 12.8 Hz, 1H), 2.17 (s, 3H), 1.63 (s, 9H); ^{13}C NMR (75 MHz, $CDCl_3$) δ 176.1, 153.1, 148.6, 140.8, 139.1, 128.2, 126.5,126.3, 125.5, 124.3, 119.1, 117.0, 113.2, 111.6, 85.6, 74.2, 55.8, 52.1, 28.1, 17.9; HRMS (ESI) m/z 477.1027 (M+H$^+$), calc. for $C_{22}H_{26}N_2O_5Br_1$ 477.1025.

The ee was determined by HPLC analysis: CHIRALPAK IC (4.6 mm i.d. x 250 mm); Hexane/2-propanol = 95/5; flow rate 1.0 mL/min; 25 ^{o}C; 254 nm; retention time: 31.4 min (major) and 55.4 min (minor).

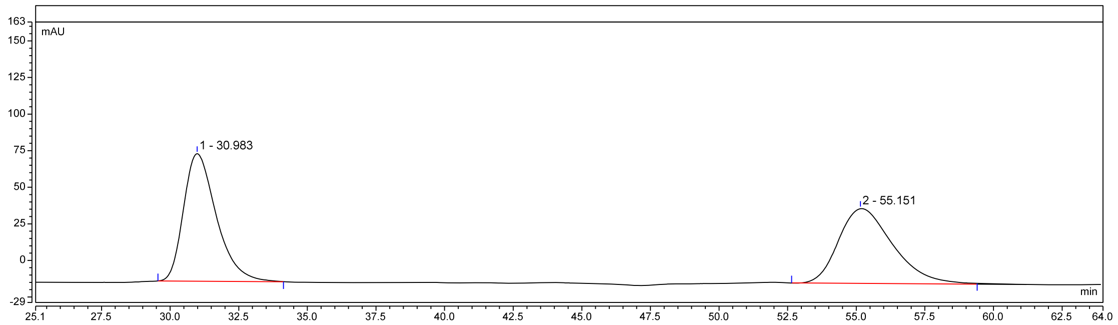

Entry	Retention Time	Area	Height	%Area
1	30.983	120.8333	87.18	50.80
2	55.151	117.0261	51.20	49.20

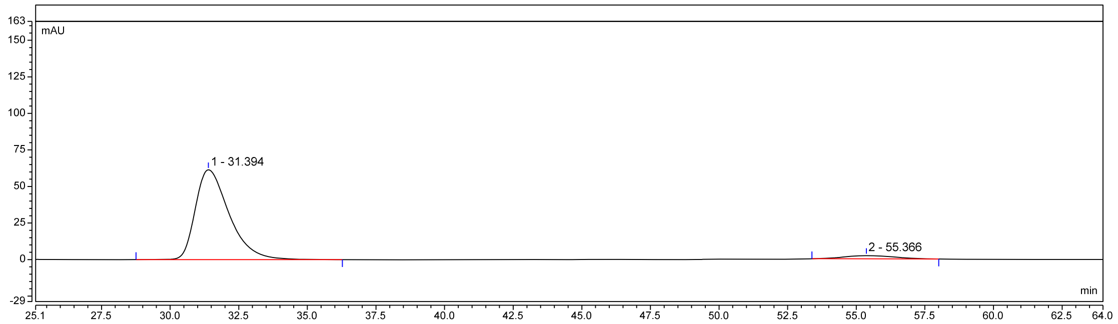

Entry	Retention Time	Area	Height	%Area
1	31.394	87.8359	61.45	94.95
2	55.366	4.6720	2.25	5.05

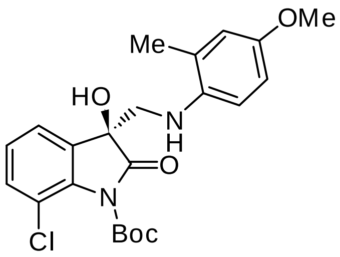

5l

Yellow solid; Mp 68.1 − 69.7 °C; 31.6 mg, 73% yield; 94% ee; $[\alpha]_D^{22}$ –173.6 (*c* 2.0, $CHCl_3$); ^1H NMR (300 MHz, $CDCl_3$) δ 7.46 – 7.31 (m, 2H), 7.16 (t, *J* = 7.8 Hz, 1H), 6.72 – 6.57 (m, 3H), 3.74 (s, 3H), 3.58 (d, *J* = 12.7 Hz, 1H), 3.36 (d, *J* = 12.8 Hz, 1H), 2.19 (s, 3H), 1.62 (s, 9H); ^{13}C NMR (75 MHz, $CDCl_3$) δ 176.6, 153.2, 147.6, 139.2, 137.0, 132.2, 131.0, 126.4, 125.9, 122.8, 119.3, 117.1, 113.1, 111.7, 86.1, 75.2, 55.8, 52.1, 27.8, 17.9; HRMS (ESI) m/z 433.1518 ($M+H^+$), calc. for $C_{22}H_{26}N_2O_5Cl_1$ 433.1530.

The ee was determined by HPLC analysis: CHIRALPAK IC (4.6 mm i.d. x 250 mm); Hexane/2-propanol = 90/10; flow rate 1.0 mL/min; 25 °C; 254 nm; retention time: 19.0 min (major) and 32.3 min (minor).

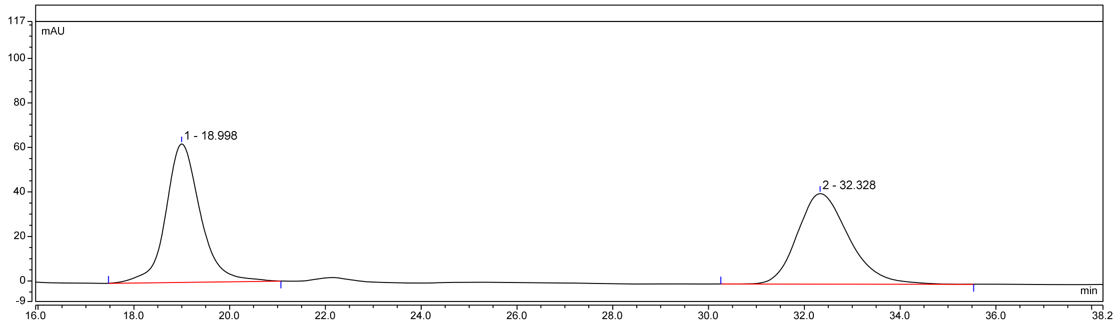

Entry	Retention Time	Area	Height	%Area
1	18.998	51.9690	62.20	50.68
2	32.328	50.5766	40.73	49.32

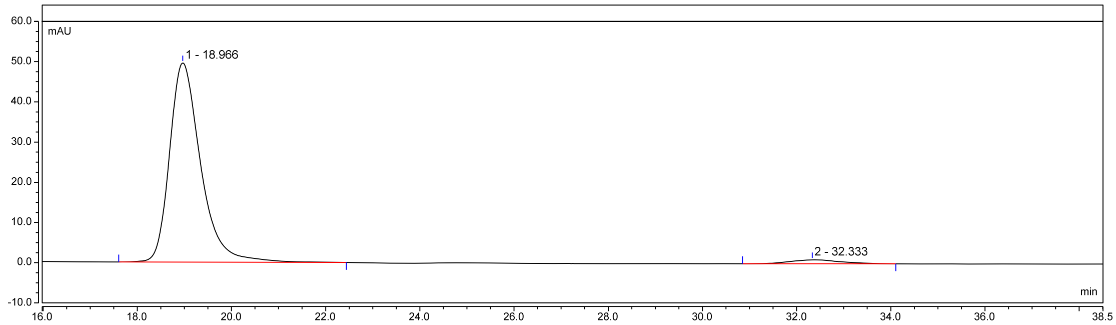

Entry	Retention Time	Area	Height	%Area
1	18.966	37.9154	49.55	97.03
2	32.333	1.1588	0.97	2.97

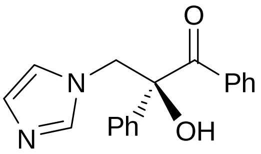

7

White solid; Mp 164.1 − 165.7 °C; 76.0 mg, 65% yield; 92% ee; $[\alpha]_D^{22}$ +54.2 (*c* 0.5, $CHCl_3$); ^{1}H NMR (300 MHz, DMSO) δ 7.89 (d, *J* = 7.5 Hz, 2H), 7.49 (t, *J* = 7.5 Hz, 1H), 7.37 – 7.24 (m, 6H), 7.14 (s, 1H), 7.05 (s, 1H), 6.68 (d, *J* = 4.1 Hz, 2H), 4.57 (q, *J* = 14.3 Hz, 2H); ^{13}C NMR (75 MHz, DMSO) δ 199.3, 139.2, 138.2, 134.3, 132.9, 130.2, 128.5, 128.1, 127.8, 127.0, 124.8, 120.8, 81.5, 54.9; HRMS (ESI) m/z 293.1283 (M+H$^+$), calc. for $C_{18}H_{17}N_2O_2$ 293.1285.

The ee was determined by HPLC analysis: CHIRALPAK OZ-H (4.6 mm i.d. x 250 mm); Hexane/2-propanol = 80/20; flow rate 1.0 mL/min; 25 °C; 254 nm; retention time: 8.2 min (major) and 11.7 min (minor).

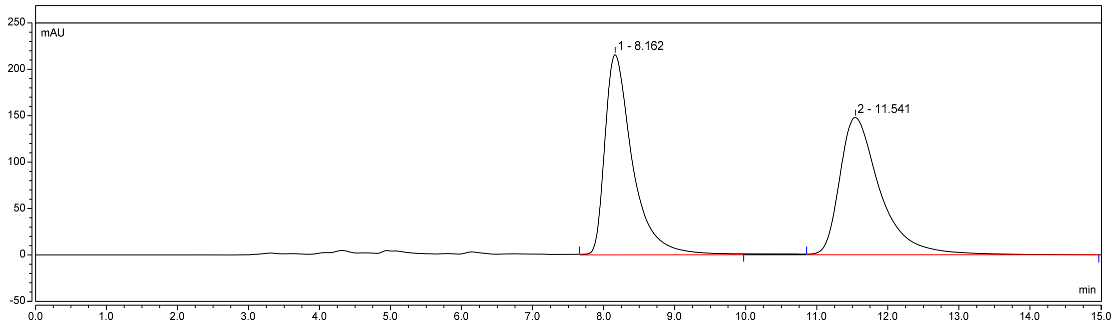

Entry	Retention Time	Area	Height	%Area
1	8.162	97.3371	215.84	49.91
2	11.541	97.6848	148.08	50.09

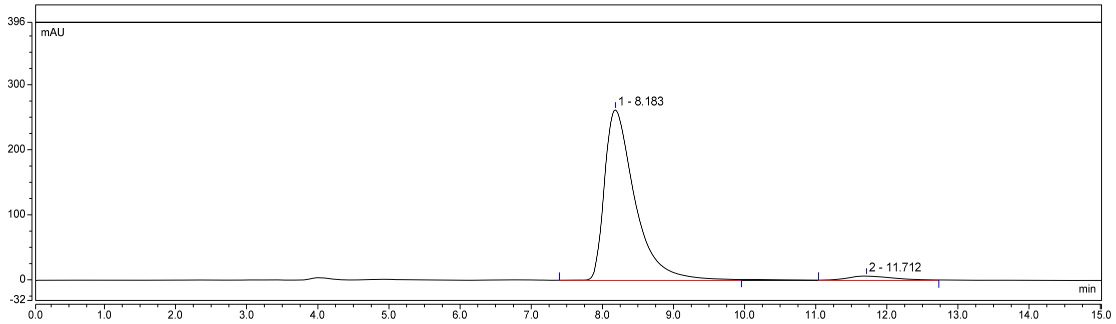

Entry	Retention Time	Area	Height	%Area
1	8.183	129.8393	262.04	96.14
2	11.712	5.2069	6.70	3.86

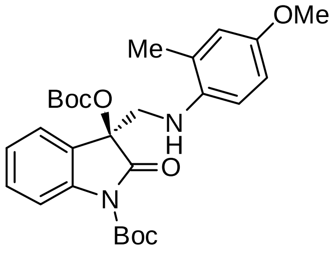

8

Yellow oil; 195.4 mg, 98% yield; 91% ee; $[\alpha]_D^{22}$ +125.0 (*c* 0.5, $CHCl_3$); ^1H NMR (300 MHz, $CDCl_3$) δ 7.90 (d, *J* = 8.2 Hz, 1H), 7.39 (t, *J* = 7.4 Hz, 2H), 7.18 (t, *J* = 7.5 Hz, 1H), 6.65 – 6.54 (m, 2H), 6.43 (d, *J* = 8.5 Hz, 1H), 3.77 (d, *J* = 13.0 Hz, 1H), 3.69 (s, 3H), 3.60 (d, *J* = 13.0 Hz, 1H), 3.37 (s, 1H), 1.99 (s, 3H), 1.61 (s, 9H), 1.36 (s, 9H); ^{13}C NMR (75 MHz, $CDCl_3$) δ 172.0, 152.3, 150.7, 148.8, 140.3, 139.2, 130.5, 125.6, 124.8, 123.0, 116.7, 115.5, 112.4, 111.5, 84.6, 84.3, 79.8, 55.7, 51.1, 28.1, 27.6, 17.6; HRMS (ESI) m/z 499.2449 ($M+H^+$), calc. for $C_{27}H_{35}N_2O_7$ 499.2444.

The ee was determined by HPLC analysis: CHIRALPAK OZ-H (4.6 mm i.d. x 250 mm); Hexane/2-propanol = 80/20; flow rate 1.0 mL/min; 25 ^oC; 254 nm; retention time: 19.0 min (major) and 23.4 min (minor).

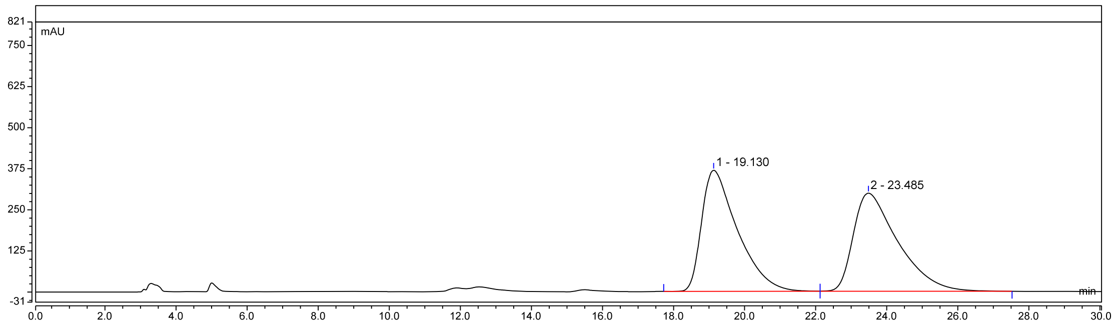

Entry	Retention Time	Area	Height	%Area
1	19.130	430.6204	368.03	49.86
2	23.485	433.0109	298.34	50.14

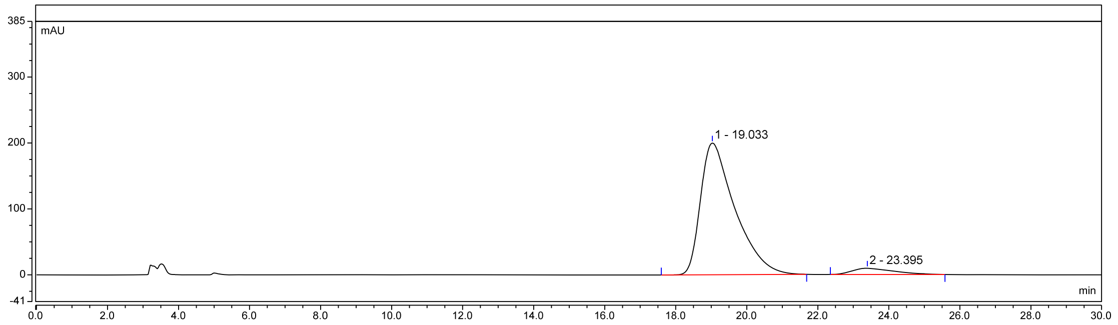

Entry	Retention Time	Area	Height	%Area
1	19.033	224.8855	199.81	95.35
2	23.395	10.9649	9.10	4.65

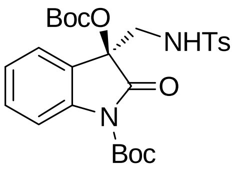

9

White solid; Mp 87.4 − 88.5 °C; 153.4 mg, 72% yield; 91% ee; $[\alpha]_D^{22}$ –24.6 (*c* 2.0, $CHCl_3$); ^{1}H NMR (300 MHz, $CDCl_3$) δ 7.83 (d, *J* = 8.1 Hz, 1H), 7.63 (d, *J* = 8.1 Hz, 2H), 7.38 (t, *J* = 8.3 Hz, 2H), 7.25 (d, *J* = 7.6 Hz, 2H), 7.15 (t, *J* = 7.5 Hz, 1H), 5.09 (s, 1H), 3.52 – 3.26 (m, 2H), 2.39 (s, 3H), 1.62 (s, 9H), 1.29 (s, 9H); ^{13}C NMR (75 MHz, $CDCl_3$) δ 170.1, 149.2, 147.6, 142.7, 138.9, 135.6, 129.8, 128.9, 126.1, 124.0, 123.3, 122.7, 114.5, 84.1, 83.6, 77.3, 47.5, 27.1, 26.5, 20.6; HRMS (ESI) m/z 533.1961 ($M+H^+$), calc. for $C_{26}H_{33}N_2O_8S_1$ 533.1958.

The ee was determined by HPLC analysis: CHIRALPAK IC (4.6 mm i.d. x 250 mm); Hexane/2-propanol = 80/20; flow rate 1.0 mL/min; 25 °C; 210 nm; retention time: 38.4 min (major) and 43.8 min (minor).

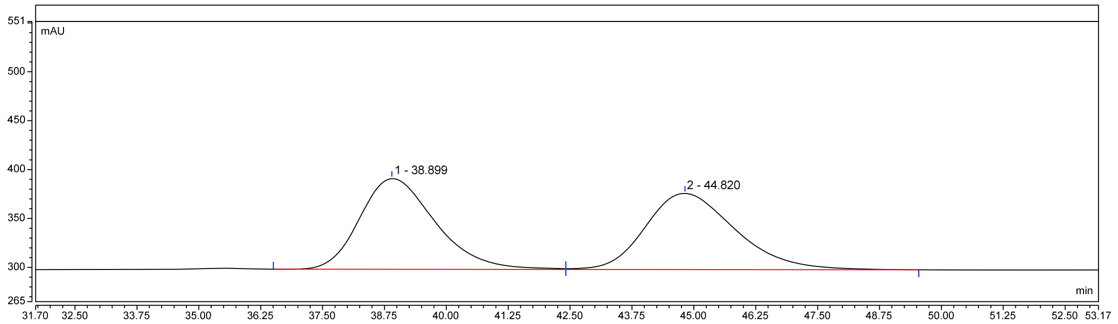

Entry	Retention Time	Area	Height	%Area
1	38.899	164.1968	92.82	50.04
2	44.820	163.9251	77.89	49.96

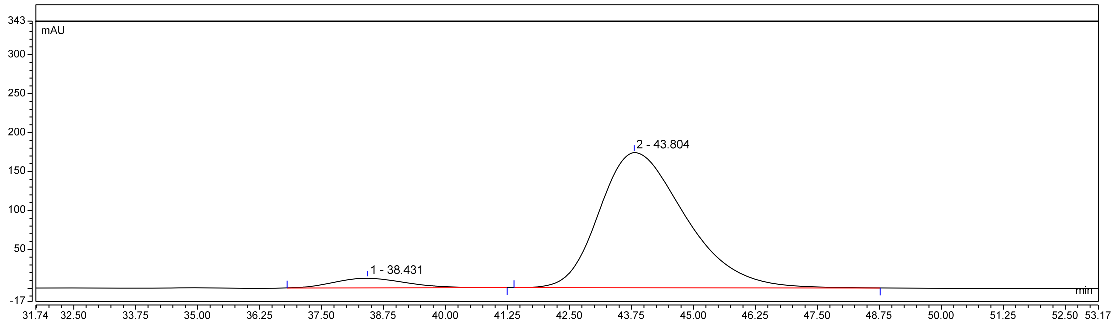

Entry	Retention Time	Area	Height	%Area
1	38.431	17.1494	11.40	4.67
2	43.804	349.8591	173.60	95.33

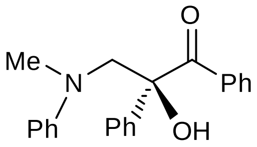

11

Yellow oil; 11.6 mg, 35% yield; 15% ee; $[\alpha]_D^{22}$ +8.1 (*c* 0.5, $CHCl_3$); ^{1}H NMR (300 MHz, $CDCl_3$) δ 7.90 (d, *J* = 7.7 Hz, 2H), 7.57 (d, *J* = 7.5 Hz, 2H), 7.47 – 7.29 (m, 4H), 7.25 – 7.11 (m, 4H), 6.80 (m, 3H), 4.44 (d, *J* = 14.6 Hz, 1H), 4.30 (s, 1H), 3.53 (d, *J* = 14.6 Hz, 1H), 2.67 (s, 3H); ^{13}C NMR (75 MHz, $CDCl_3$) δ 201.1, 141.7, 135.37, 135.07, 132.8, 130.7, 130.1, 129.3, 129.1, 128.1, 128.0, 124.8, 115.1, 82.1, 64.0. 34.0; HRMS (ESI) m/z 332.1642 ($M+H^+$), calc. for $C_{22}H_{22}N_2O_2$ 332.1645.

The ee was determined by HPLC analysis: CHIRALPAK IC (4.6 mm i.d. x 250 mm); Hexane/2-propanol = 97/3; flow rate 1.0 mL/min; 25 ^{o}C; 254 nm; retention time: 5.3 min (minor) and 6.5 min (major).

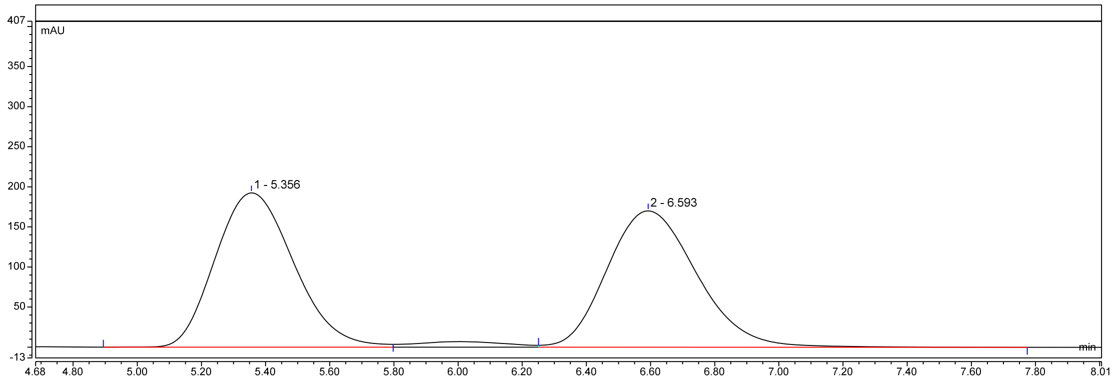

Entry	Retention Time	Area	Height	%Area
1	5.356	54.6084	192.47	49.69
2	6.593	55.2937	170.06	50.31

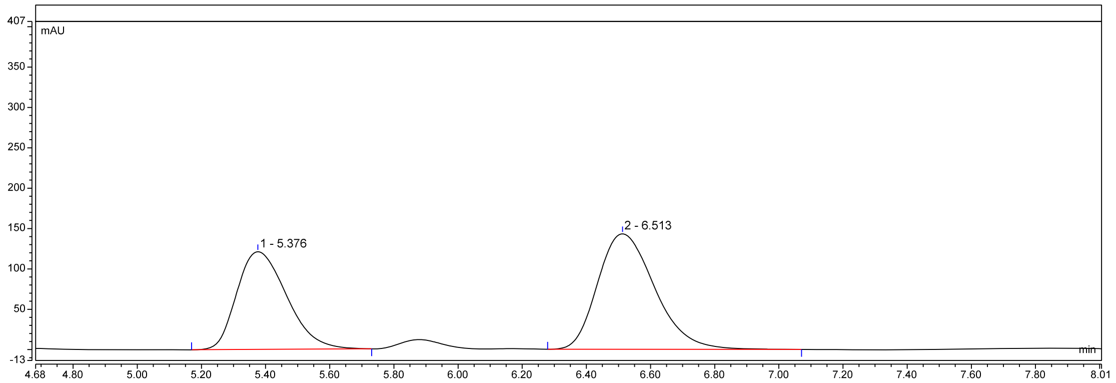

Entry	Retention Time	Area	Height	%Area
1	5.376	21.7741	120.98	42.51
2	6.513	29.4453	143.04	57.49

8. Copies of NMR spectra

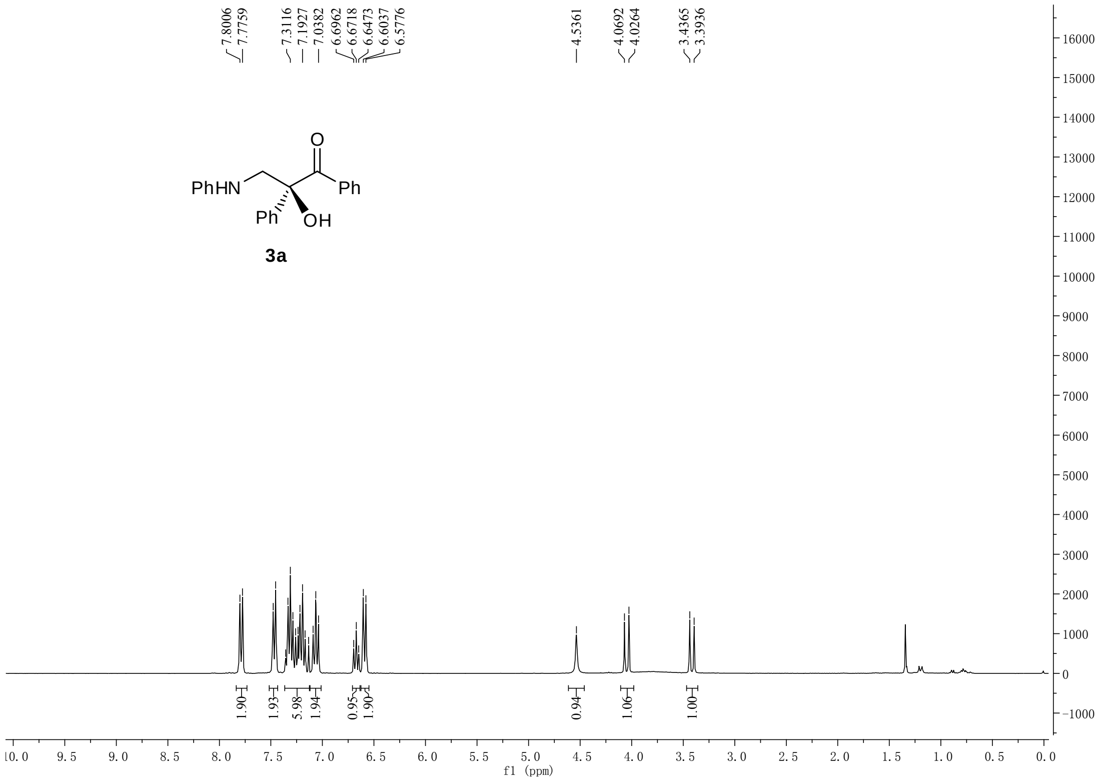

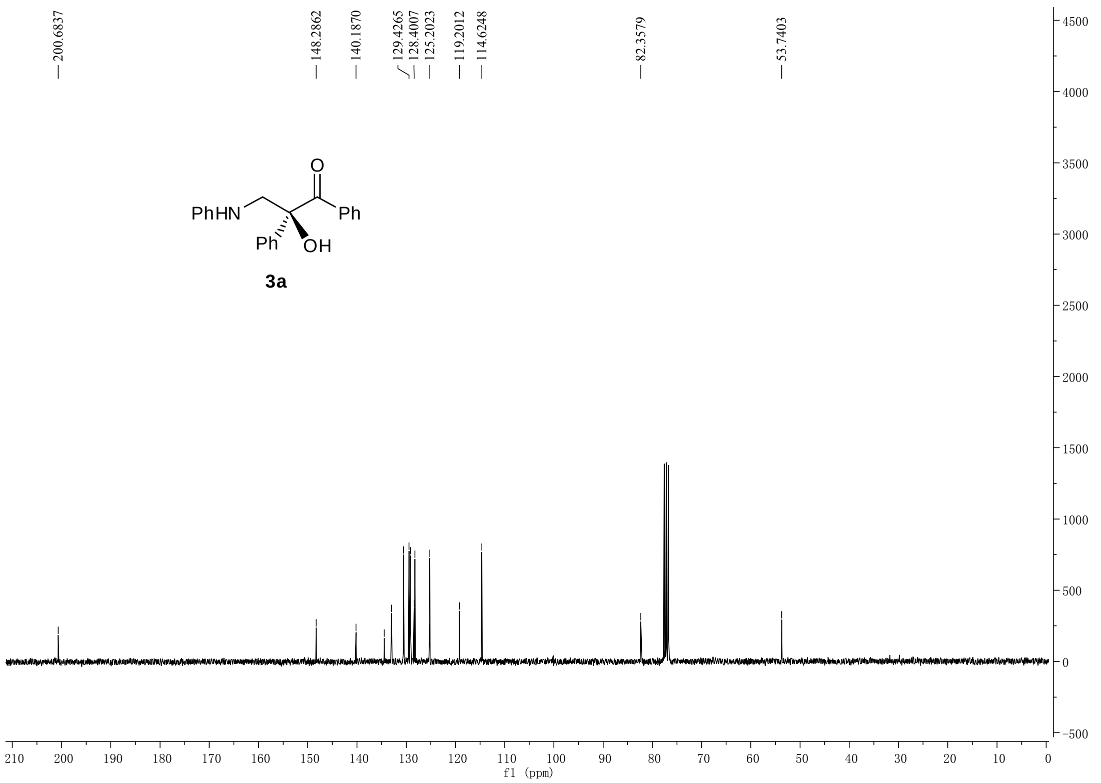

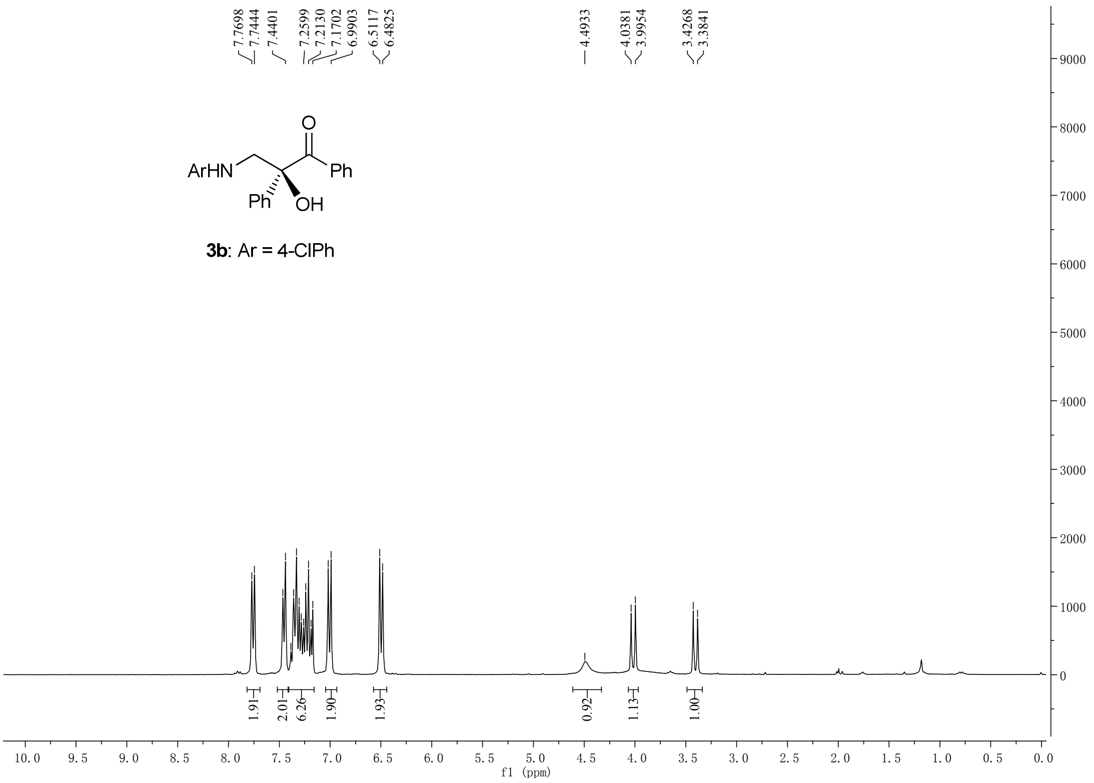

3b: Ar = 4-ClPh

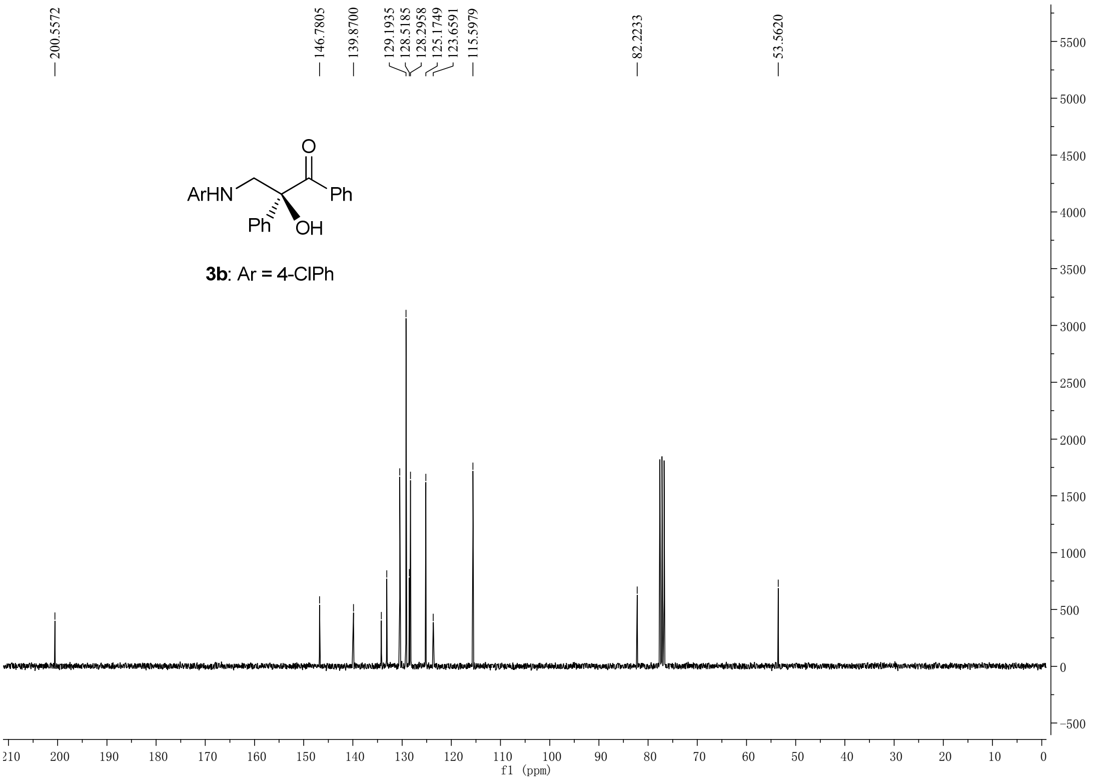

3b: Ar = 4-ClPh

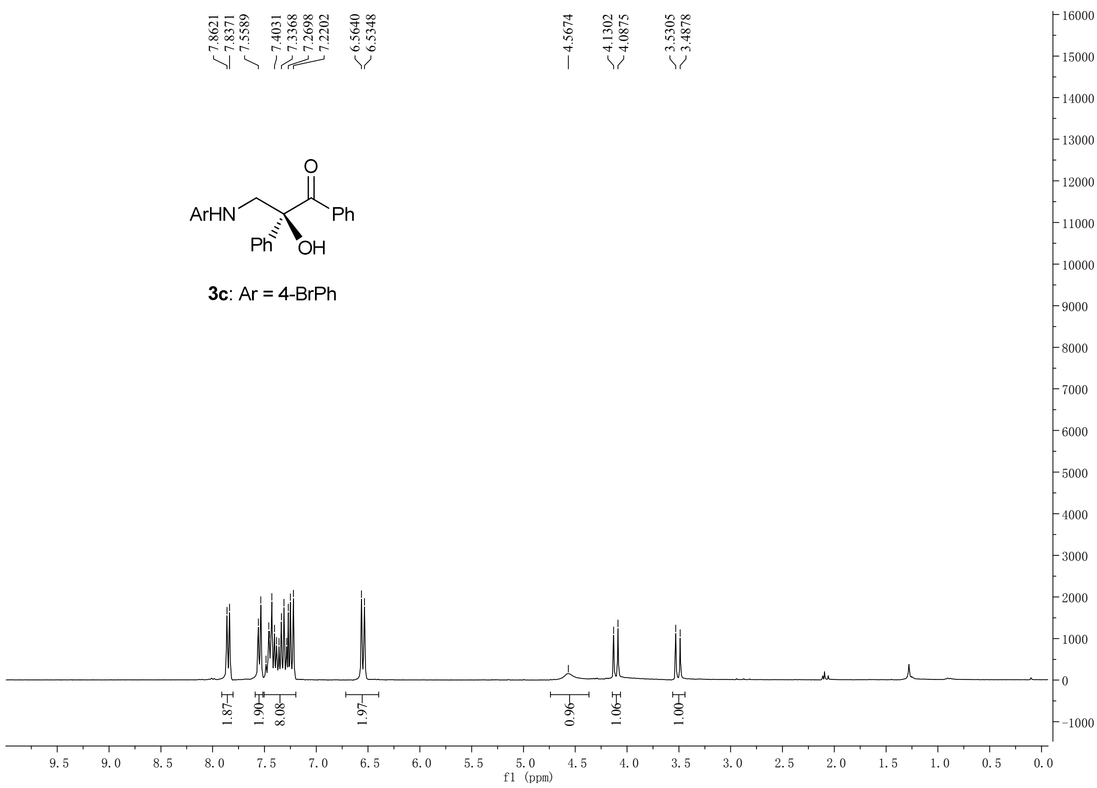
7.8621
7.8371
7.5589
7.4031
7.3368
7.2698
7.2202
6.5640
6.5348
4.5674
4.1302
4.0875
3.5305
3.4878
ArHN
O
Ph
Ph
OH
3c: Ar = 4-BrPh
1.87
1.90
8.08
1.97
0.96
1.06
1.00
f1 (ppm)

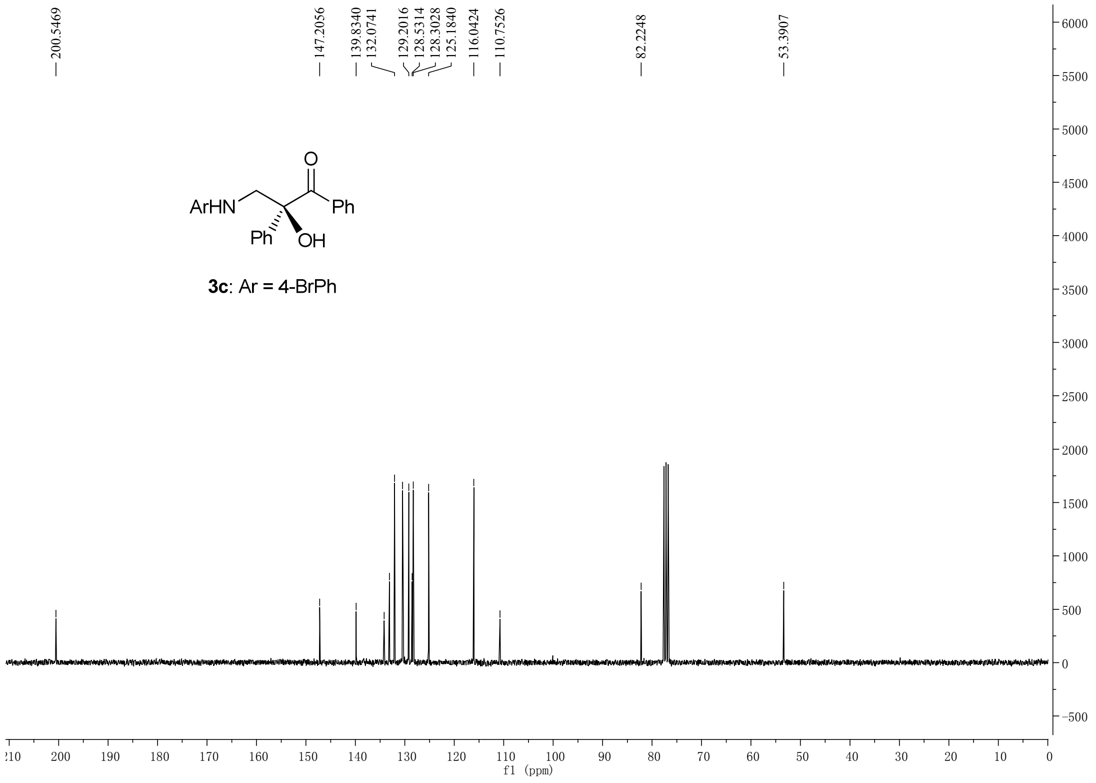
200.5469
147.2056
139.8340
132.0741
129.2016
128.5314
128.3028
125.1840
116.0424
110.7526
82.2248
53.3907
ArHN
O
Ph
Ph
OH
3c: Ar = 4-BrPh
f1 (ppm)

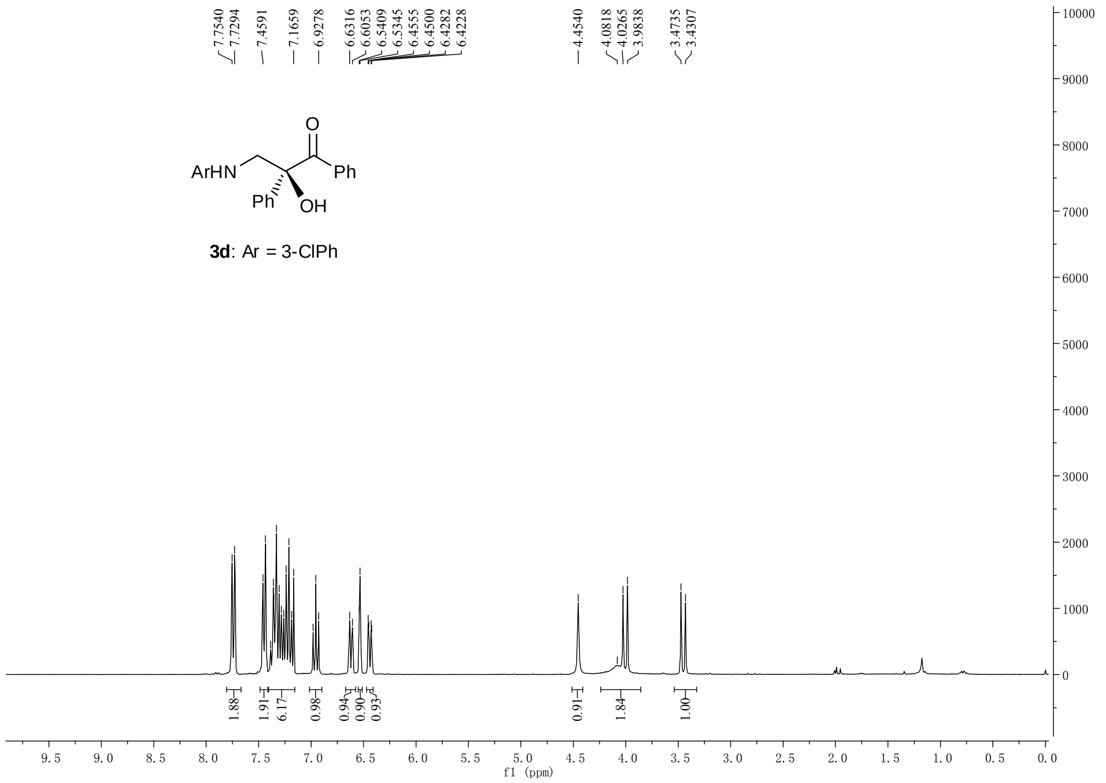
7.7540
7.7294
7.4591
7.1659
6.9278
6.6316
6.6053
6.5409
6.5345
6.4555
6.4500
6.4282
6.4228
4.4540
4.0818
4.0265
3.9838
3.4735
3.4307
ArHN
O
Ph
Ph
OH
3d: Ar = 3-ClPh
1.88
1.91
6.17
0.98
0.94
0.90
0.93
0.91
1.84
1.00
f1 (ppm)

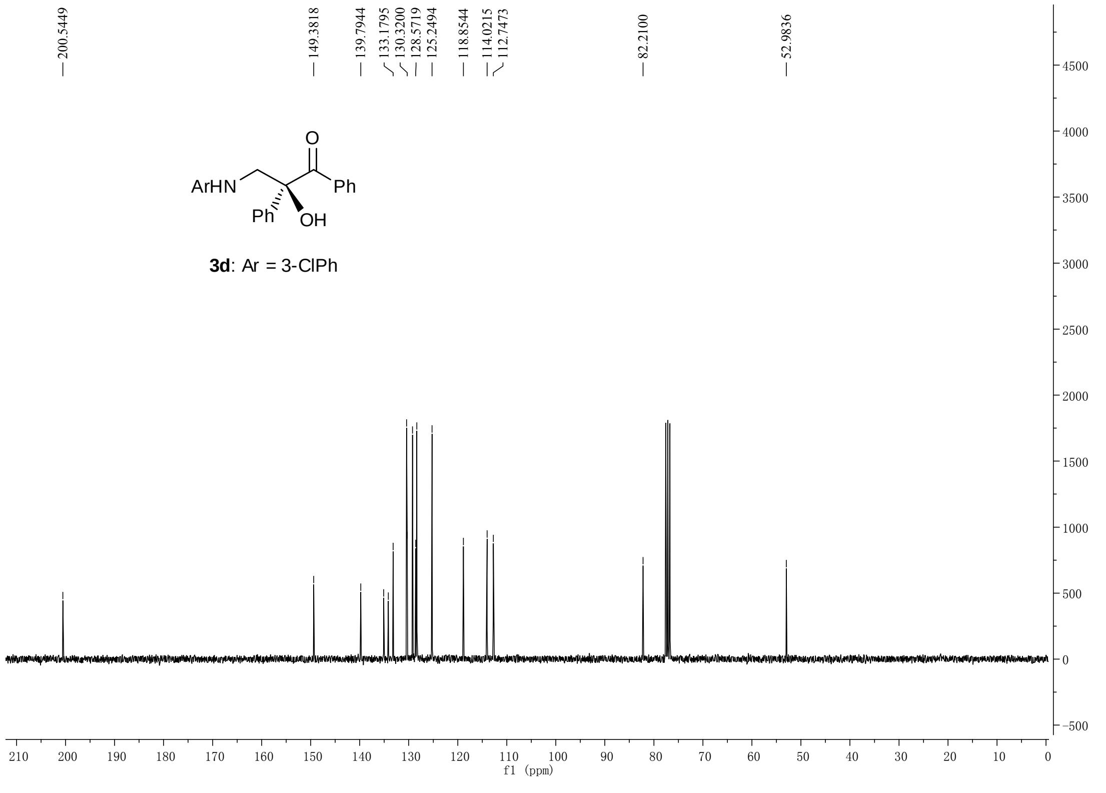
200.5449
149.3818
139.7944
133.1795
130.3200
128.5719
125.2494
118.8544
114.0215
112.7473
82.2100
52.9836
ArHN
O
Ph
Ph
OH
3d: Ar = 3-ClPh
f1 (ppm)

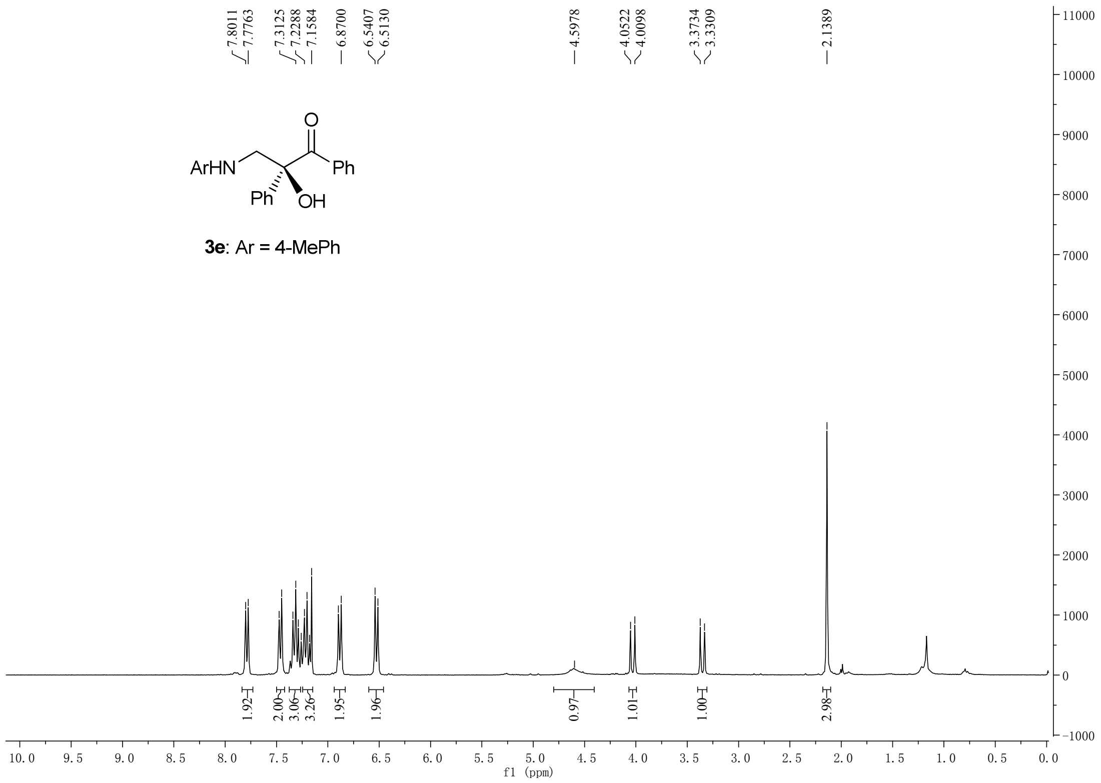
7.8011
7.7763
7.3125
7.2288
7.1584
6.8700
6.5407
6.5130
4.5978
4.0522
4.0098
3.3734
3.3309
2.1389
ArHN
O
Ph
Ph
OH
3e: Ar = 4-MePh
1.92
2.00
3.06
3.26
1.95
1.96
0.97
1.01
1.00
2.98
f1 (ppm)

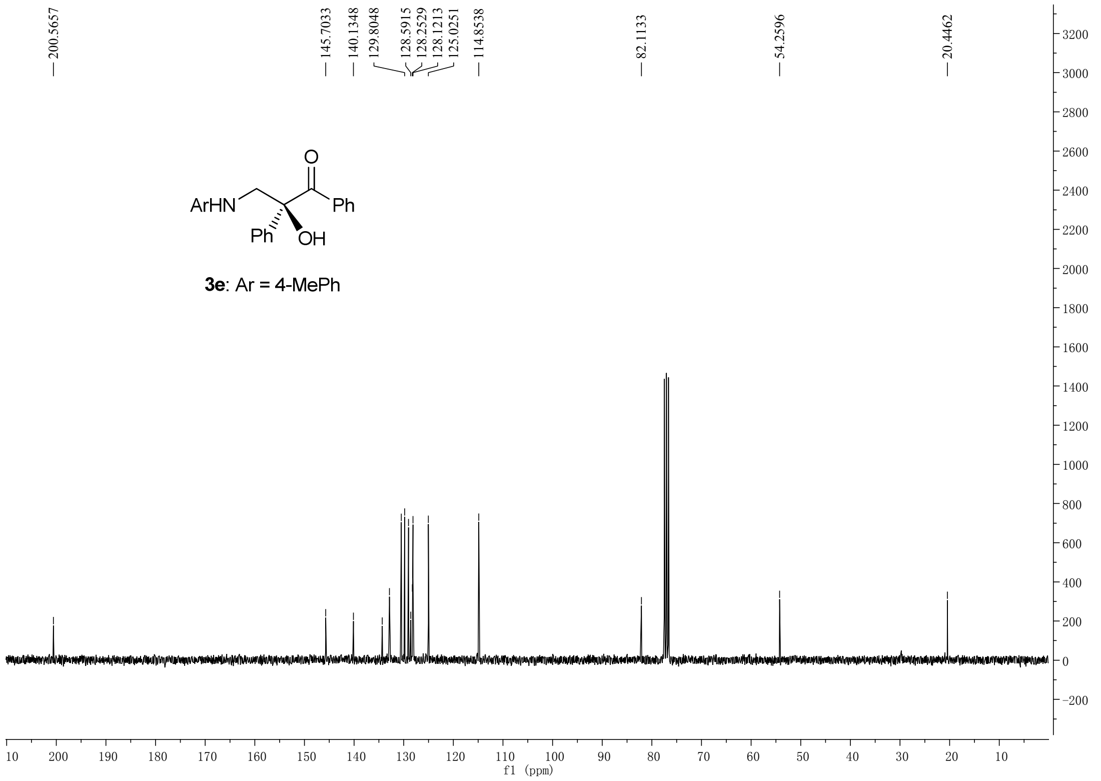
200.5657
145.7033
140.1348
129.8048
128.5915
128.2529
128.1213
125.0251
114.8538
82.1133
54.2596
20.4462
ArHN
O
Ph
Ph
OH
3e: Ar = 4-MePh
f1 (ppm)

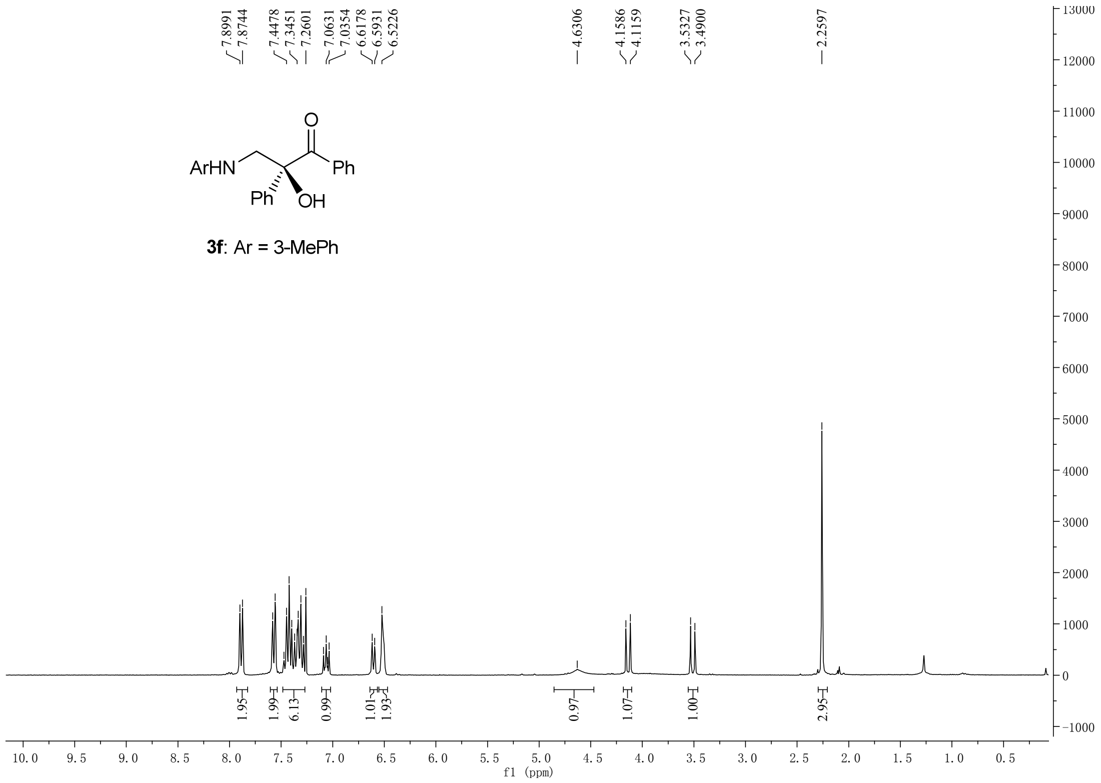
7.8991
7.8744
7.4478
7.3451
7.2601
7.0631
7.0354
6.6178
6.5931
6.5226
4.6306
4.1586
4.1159
3.5327
3.4900
2.2597
ArHN
O
Ph
Ph
OH
3f: Ar = 3-MePh
1.95
1.99
6.13
0.99
1.01
1.93
0.97
1.07
1.00
2.95
f1 (ppm)

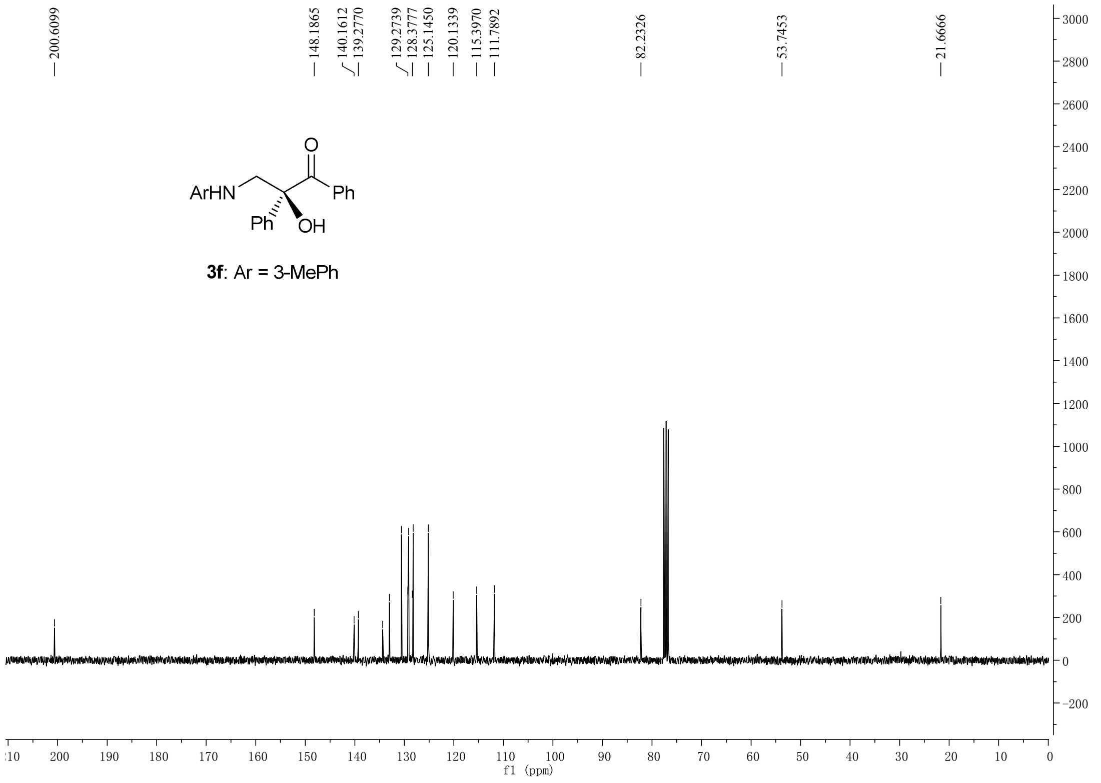
200.6099
148.1865
140.1612
139.2770
129.2739
128.3777
125.1450
120.1339
115.3970
111.7892
82.2326
53.7453
21.6666
ArHN
O
Ph
Ph
OH
3f: Ar = 3-MePh
f1 (ppm)

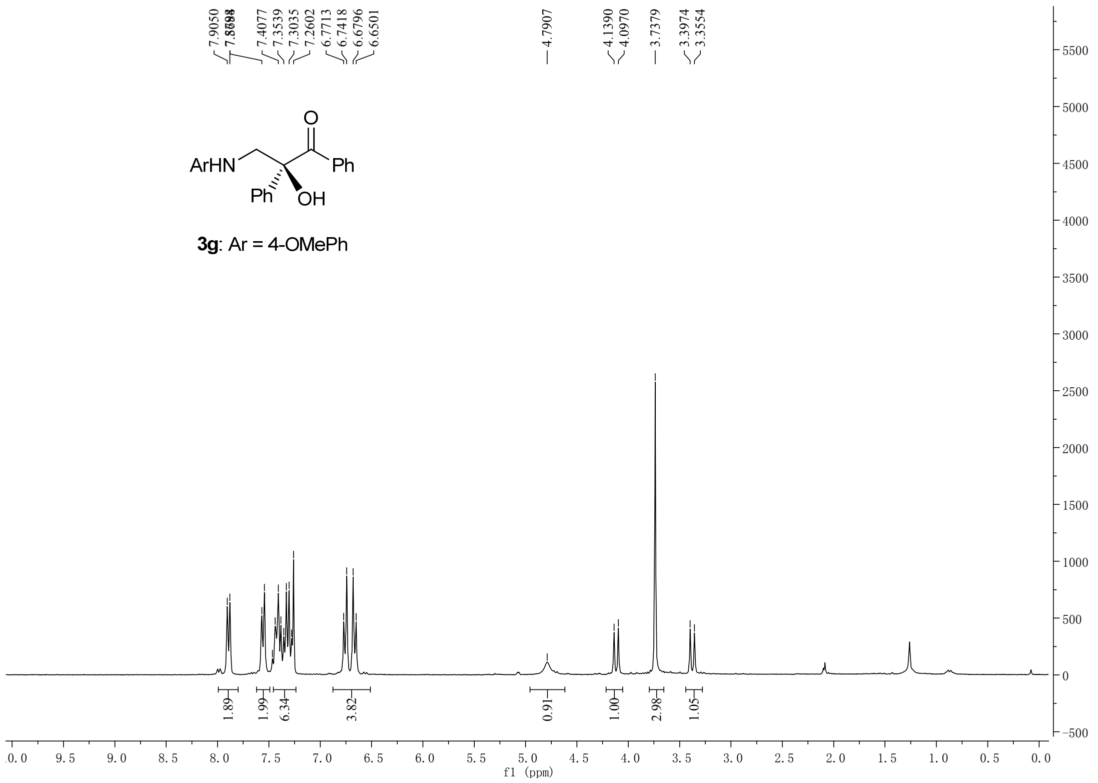
7.9050
7.4077
7.3539
7.3035
7.2602
6.7713
6.7418
6.6796
6.6501
4.7907
4.1390
4.0970
3.7379
3.3974
3.3554
ArHN
O
Ph
Ph
OH
3g: Ar = 4-OMePh
1.89
1.99
6.34
3.82
0.91
1.00
2.98
1.05
f1 (ppm)

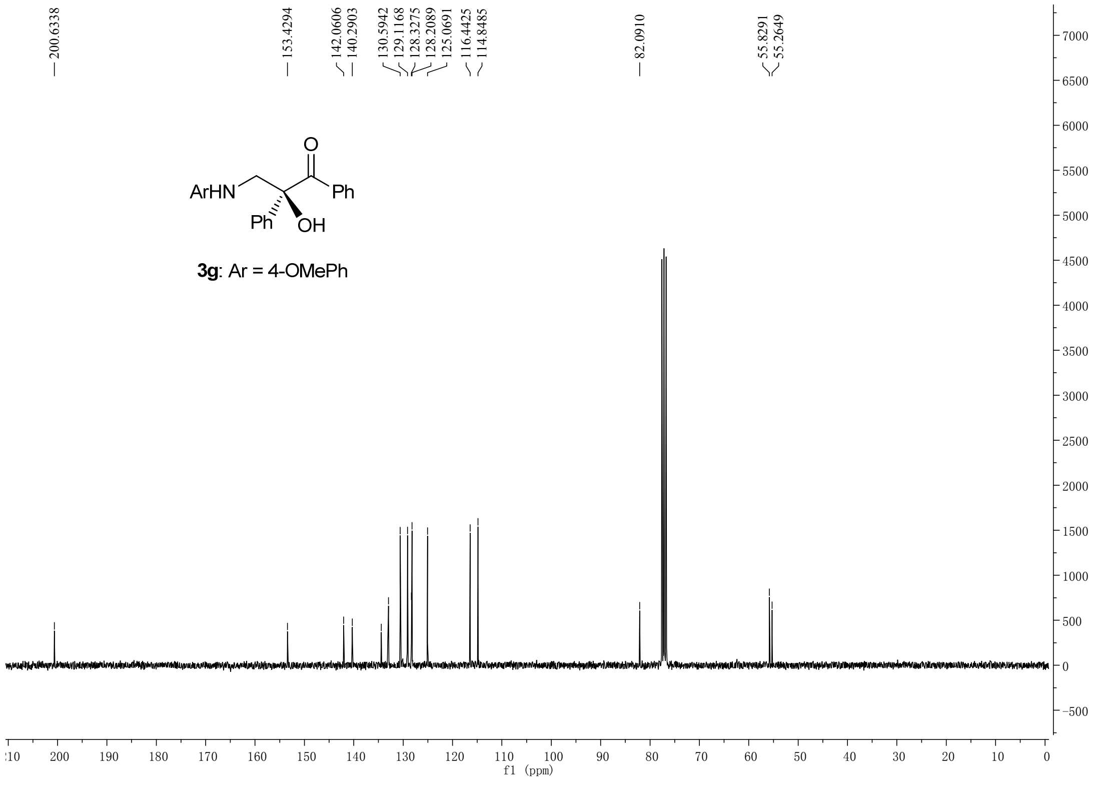
200.6338
153.4294
142.0606
140.2903
130.5942
129.1168
128.3275
128.2089
125.0691
116.4425
114.8485
82.0910
55.8291
55.2649
ArHN
O
Ph
Ph
OH
3g: Ar = 4-OMePh
f1 (ppm)

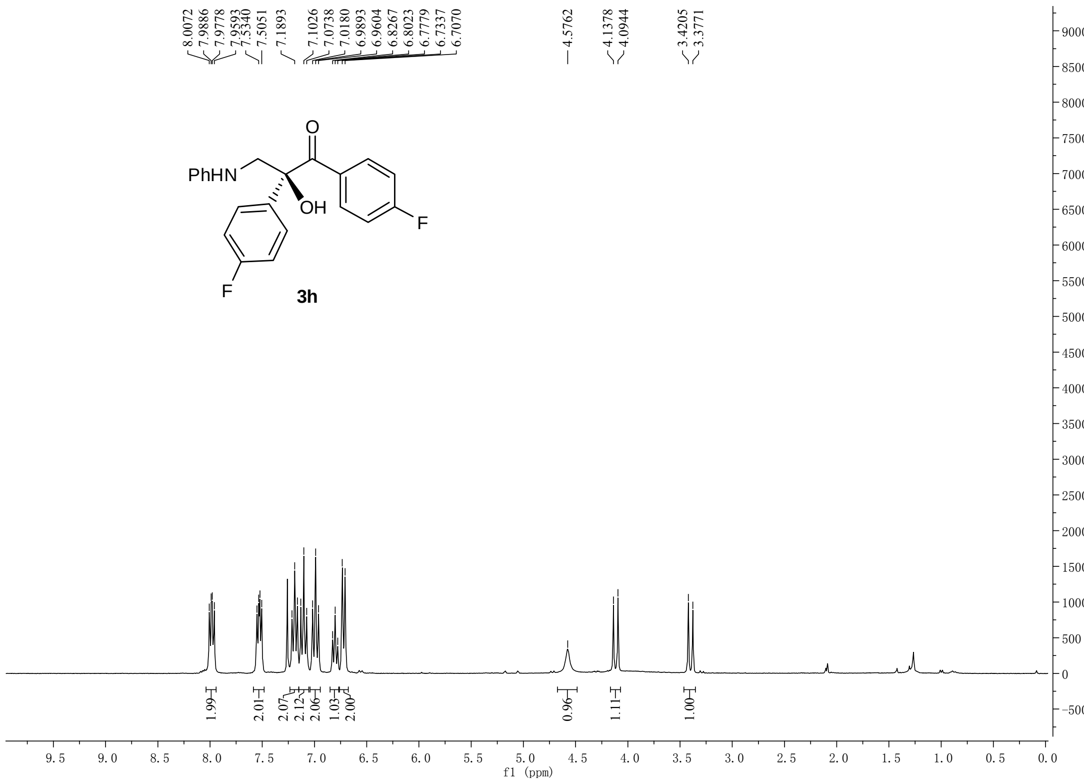
8.0072
7.9886
7.9778
7.9593
7.5340
7.5051
7.1893
7.1026
7.0738
7.0180
6.9893
6.9604
6.8267
6.8023
6.7779
6.7337
6.7070
4.5762
4.1378
4.0944
3.4205
3.3771
PhHN
O
OH
F
F
3h
1.99
2.01
2.07
2.12
2.06
1.03
2.00
0.96
1.11
1.00
f1 (ppm)

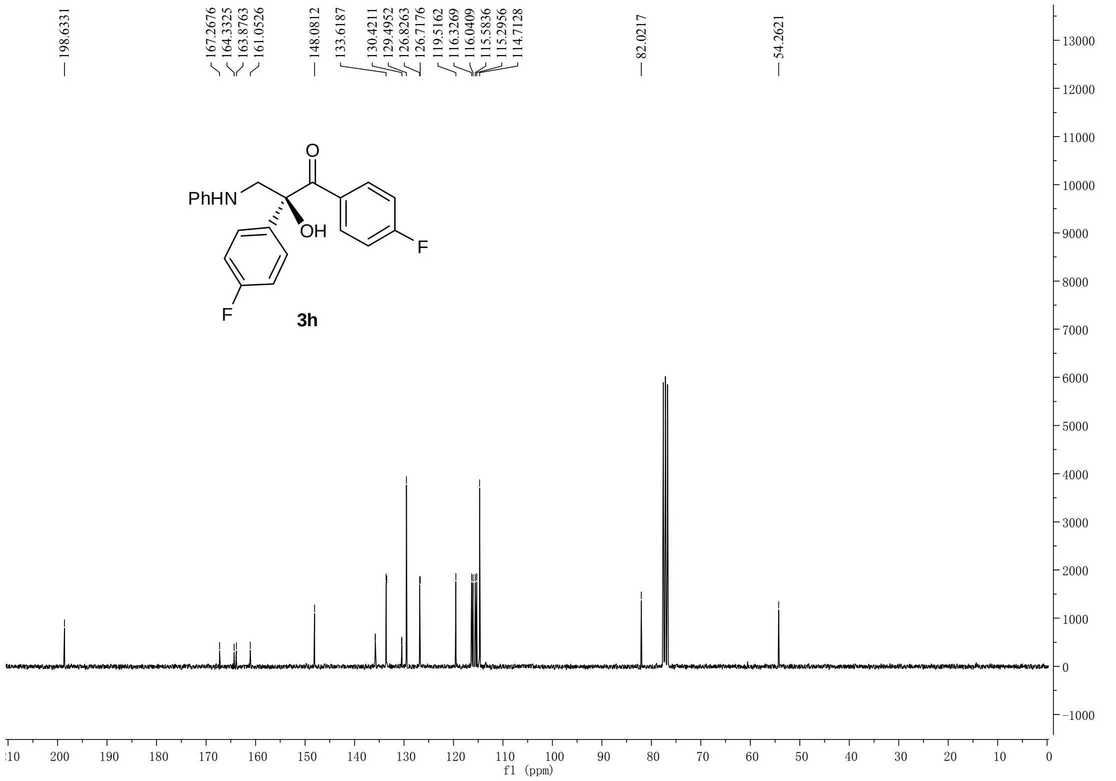
198.6331
167.2676
164.3325
163.8763
161.0526
148.0812
133.6187
130.4211
129.4952
126.8263
126.7176
119.5162
116.3269
116.0409
115.5836
115.2956
114.7128
82.0217
54.2621
PhHN
O
OH
F
F
3h
f1 (ppm)

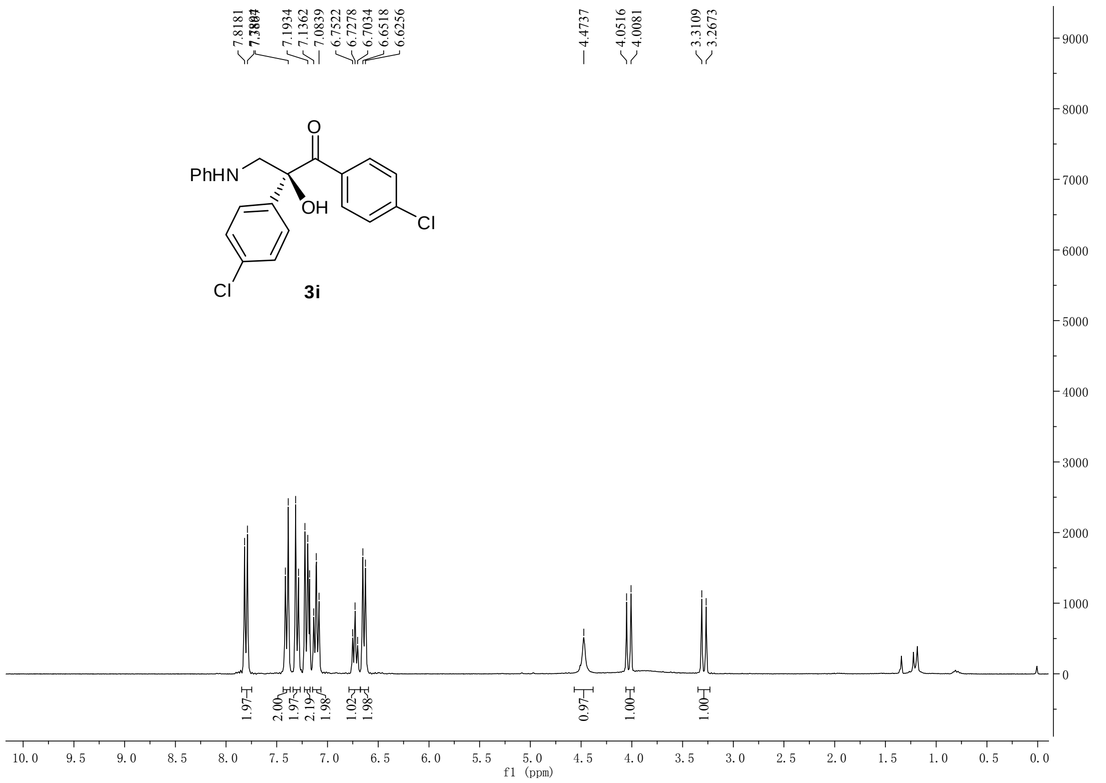
7.8181
7.1934
7.1362
7.0839
6.7522
6.7278
6.7034
6.6518
6.6256
4.4737
4.0516
4.0081
3.3109
3.2673
PhHN
O
OH
Cl
Cl
3i
1.97
2.00
1.97
2.19
1.98
1.02
1.98
0.97
1.00
1.00
f1 (ppm)

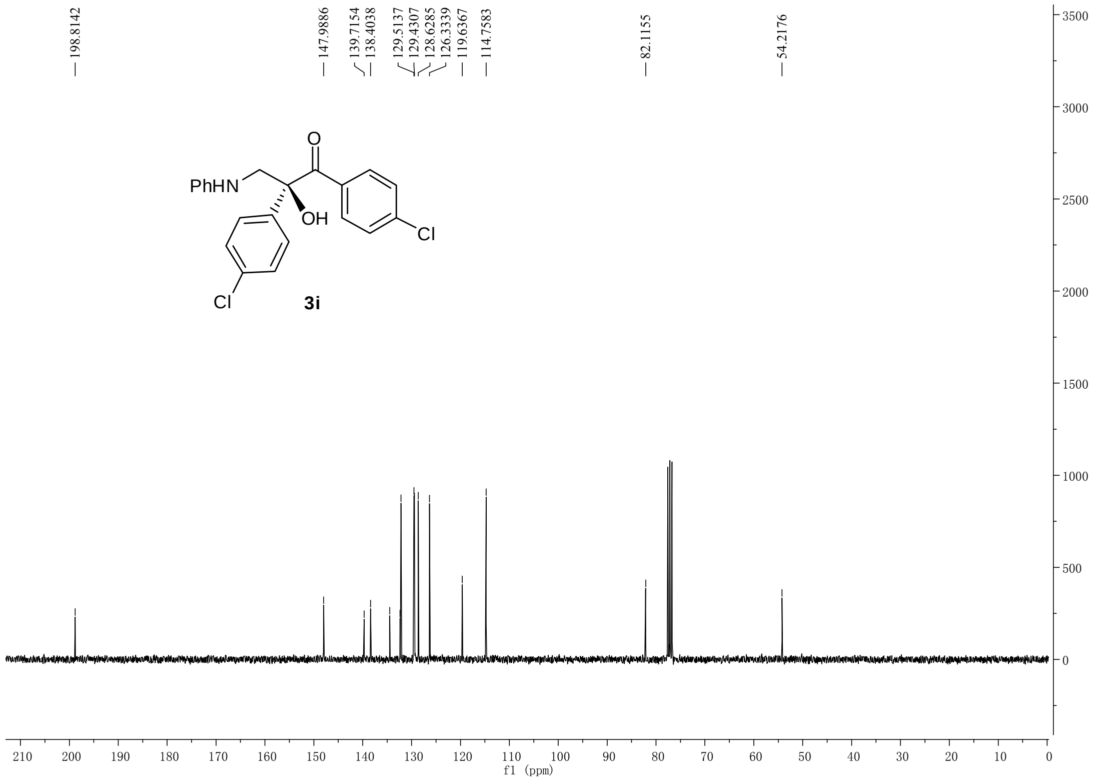
198.8142
147.9886
139.7154
138.4038
129.5137
129.4307
128.6285
126.3339
119.6367
114.7583
82.1155
54.2176
PhHN
O
OH
Cl
Cl
3i
f1 (ppm)

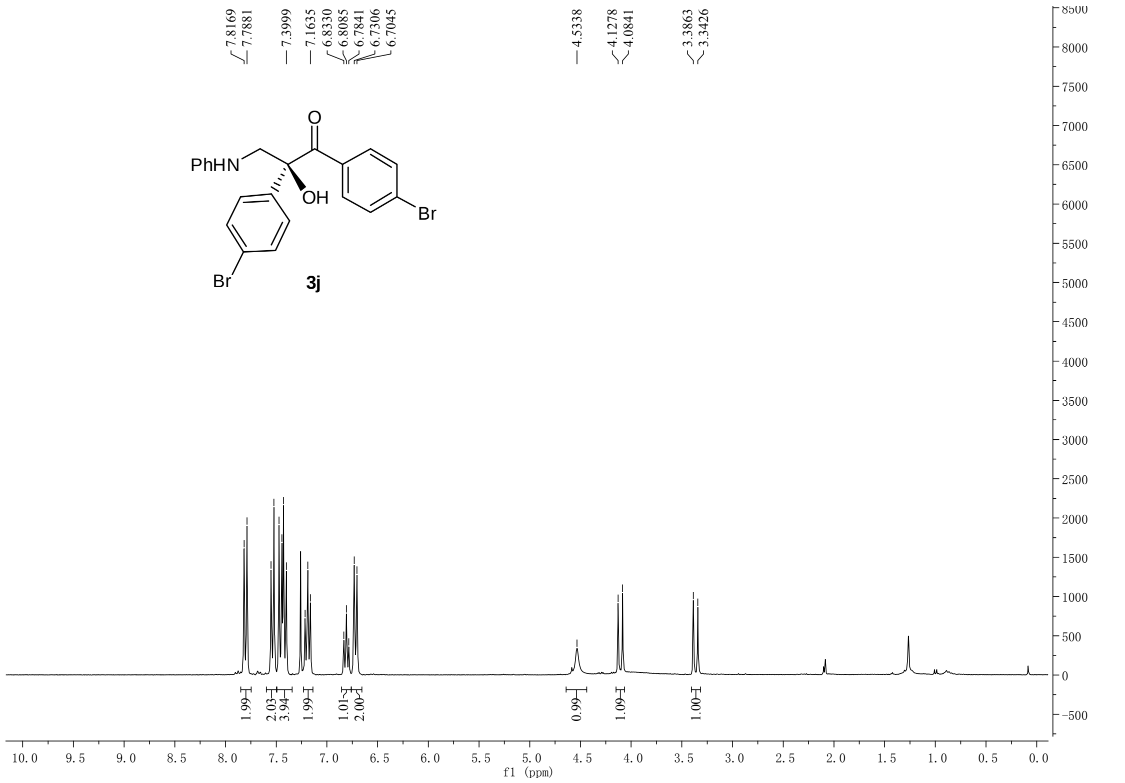
7.8169
7.7881
7.3999
7.1635
6.8330
6.8085
6.7841
6.7306
6.7045
4.5338
4.1278
4.0841
3.3863
3.3426
PhHN
O
OH
Br
Br
3j
1.99
2.03
3.94
1.99
1.01
2.00
0.99
1.09
1.00
f1 (ppm)

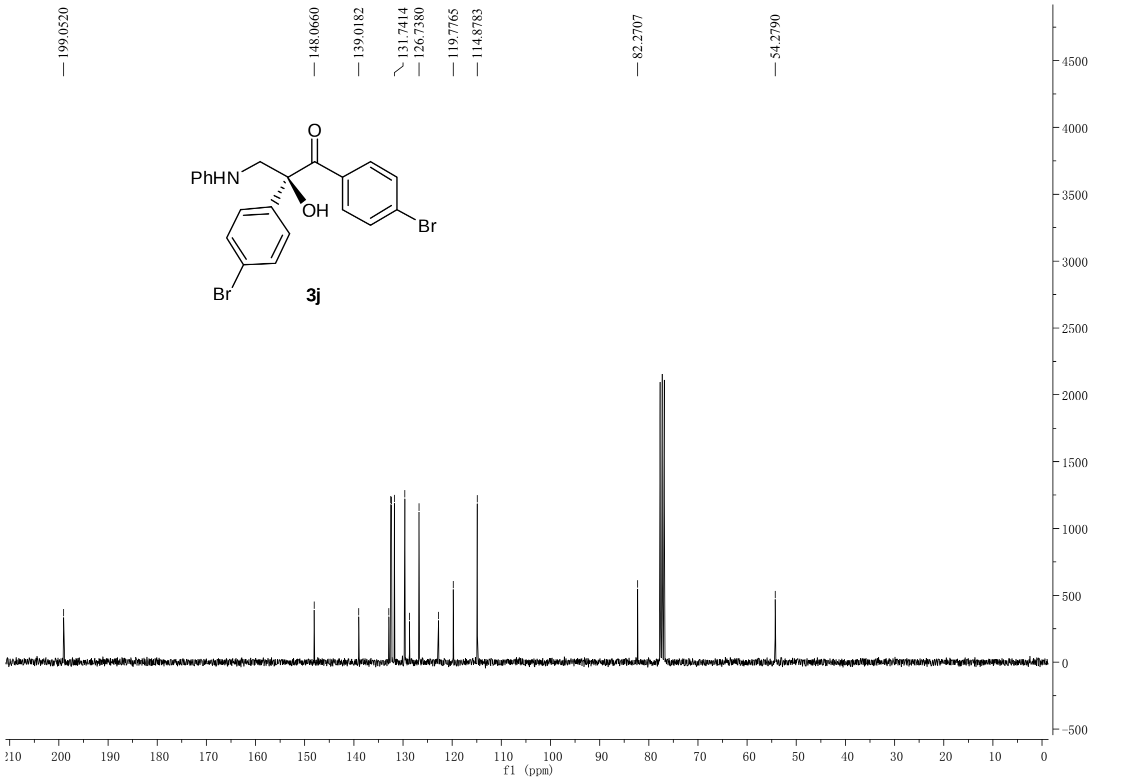
199.0520
148.0660
139.0182
131.7414
126.7380
119.7765
114.8783
82.2707
54.2790
PhHN
O
OH
Br
Br
3j
f1 (ppm)

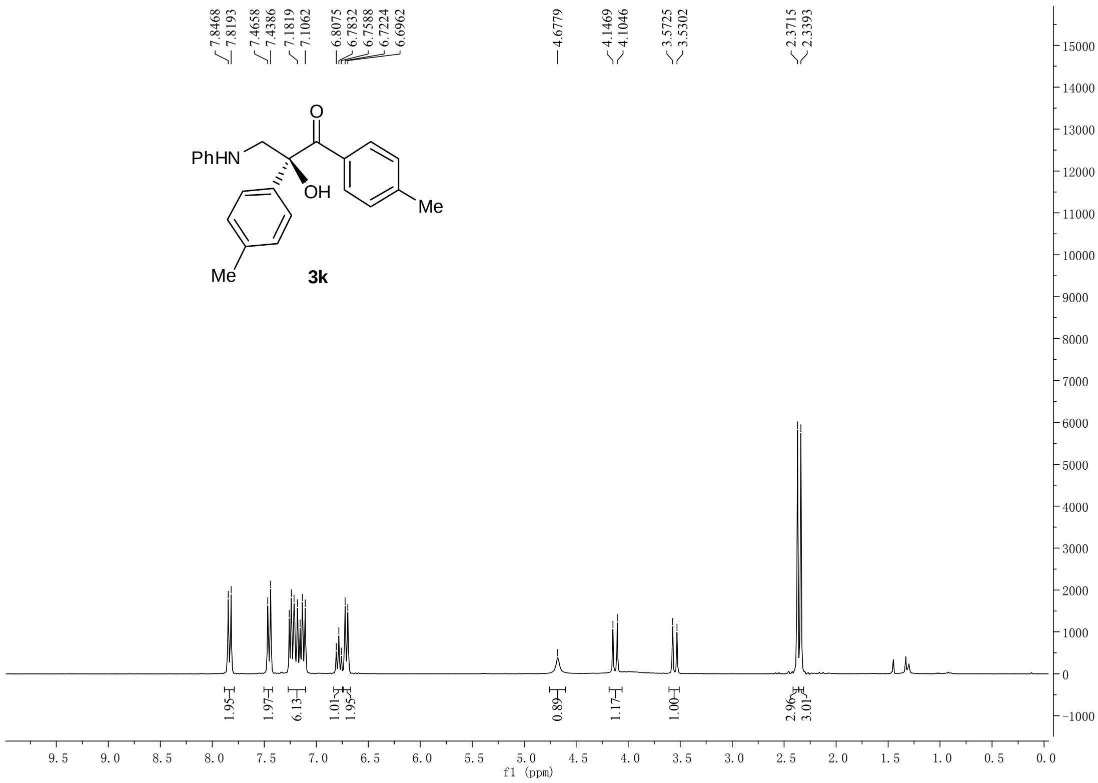
7.8468
7.8193
7.4658
7.4386
7.1819
7.1062
6.8075
6.7832
6.7588
6.7224
6.6962
4.6779
4.1469
4.1046
3.5725
3.5302
2.3715
2.3393
PhHN
O
OH
Me
Me
3k
1.95
1.97
6.13
1.01
1.95
0.89
1.17
1.00
2.96
3.01
f1 (ppm)

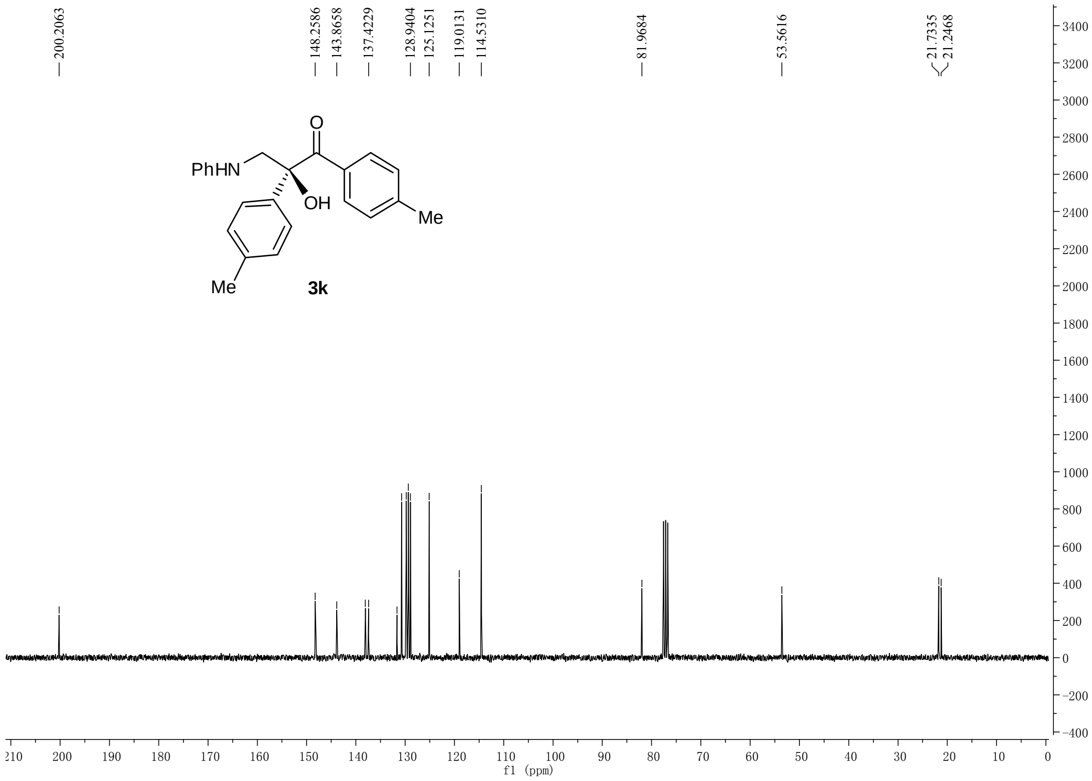
200.2063
148.2586
143.8658
137.4229
128.9404
125.1251
119.0131
114.5310
81.9684
53.5616
21.7335
21.2468
PhHN
O
OH
Me
Me
3k
f1 (ppm)

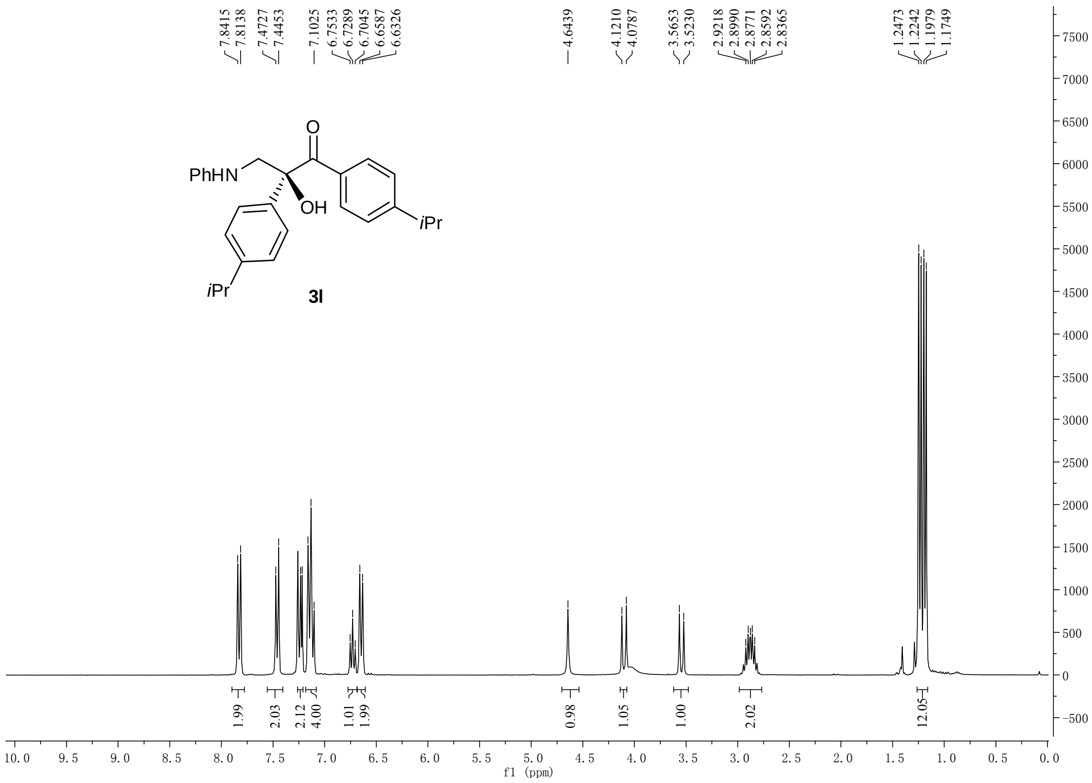
7.8415
7.8138
7.4727
7.4453
7.1025
6.7533
6.7289
6.7045
6.6587
6.6326
4.6439
4.1210
4.0787
3.5653
3.5230
2.9218
2.8990
2.8771
2.8592
2.8365
1.2473
1.2242
1.1979
1.1749
O
PhHN
OH
iPr
iPr
3l
1.99
2.03
2.12
4.00
1.01
1.99
0.98
1.05
1.00
2.02
12.05
f1 (ppm)

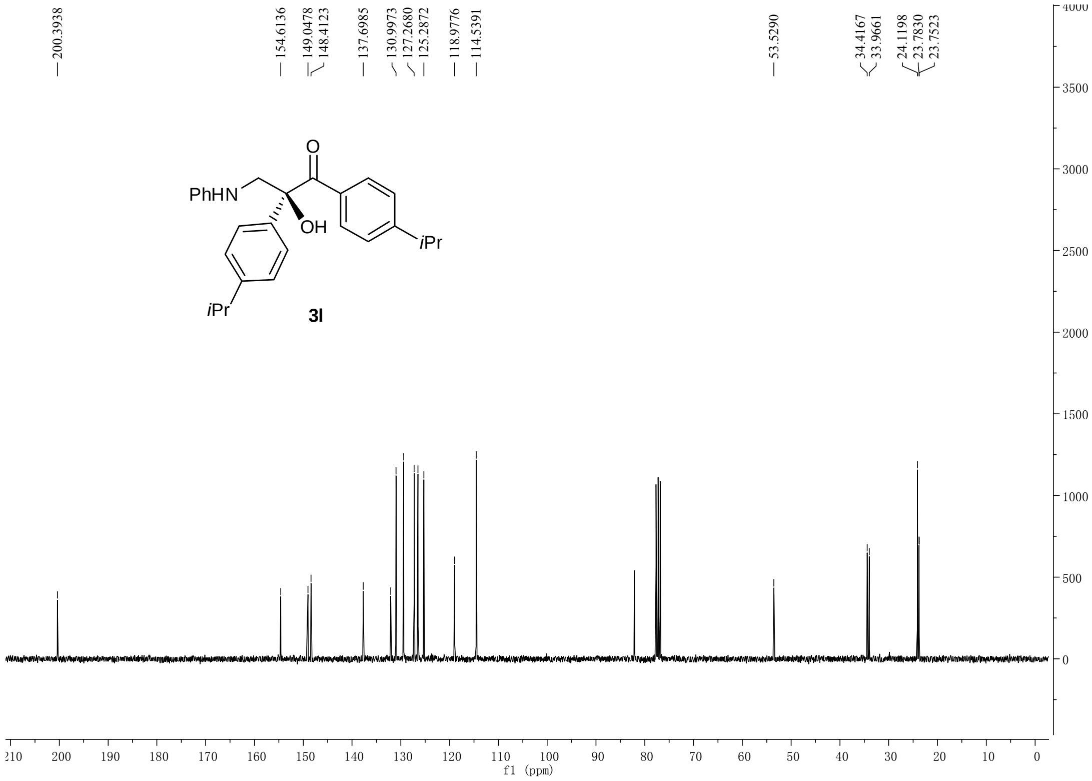
200.3938
154.6136
149.0478
148.4123
137.6985
130.9973
127.2680
125.2872
118.9776
114.5391
53.5290
34.4167
33.9661
24.1198
23.7830
23.7523
O
PhHN
OH
iPr
iPr
3l
f1 (ppm)

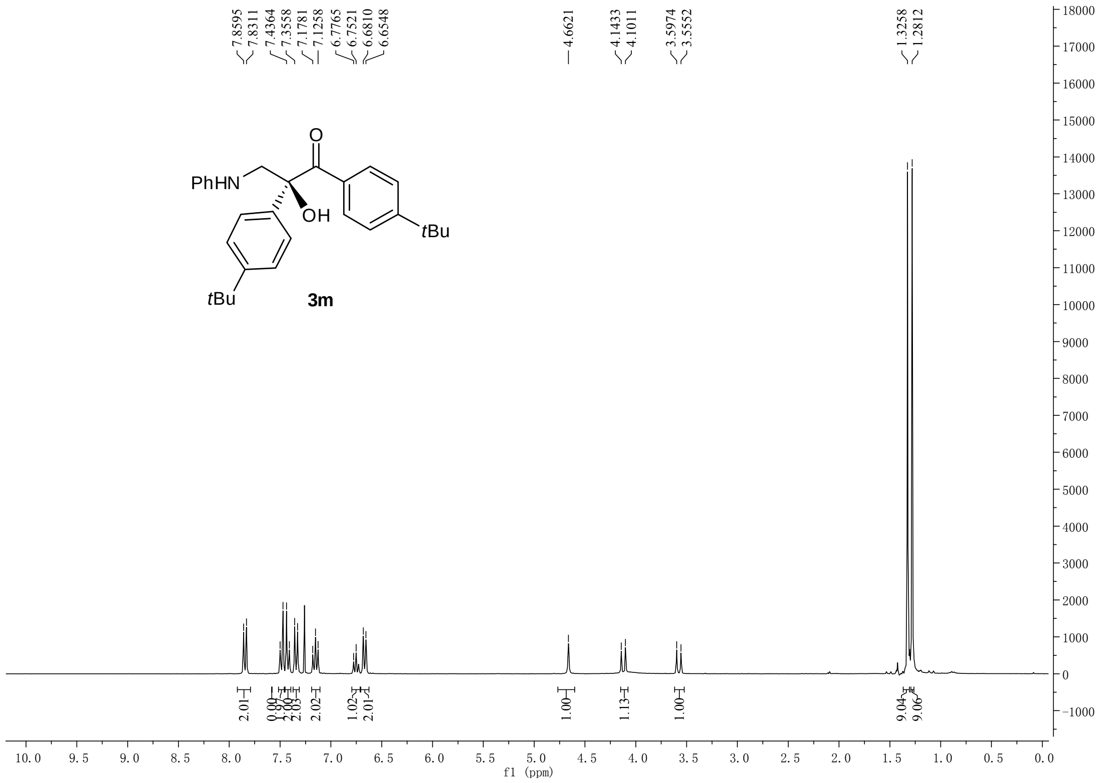
7.8595
7.8311
7.4364
7.3558
7.1781
7.1258
6.7765
6.7521
6.6810
6.6548
4.6621
4.1433
4.1011
3.5974
3.5552
1.3258
1.2812
PhHN
O
OH
tBu
tBu
3m
2.01
2.02
1.02
2.01
1.00
1.13
1.00
9.04
9.06
f1 (ppm)

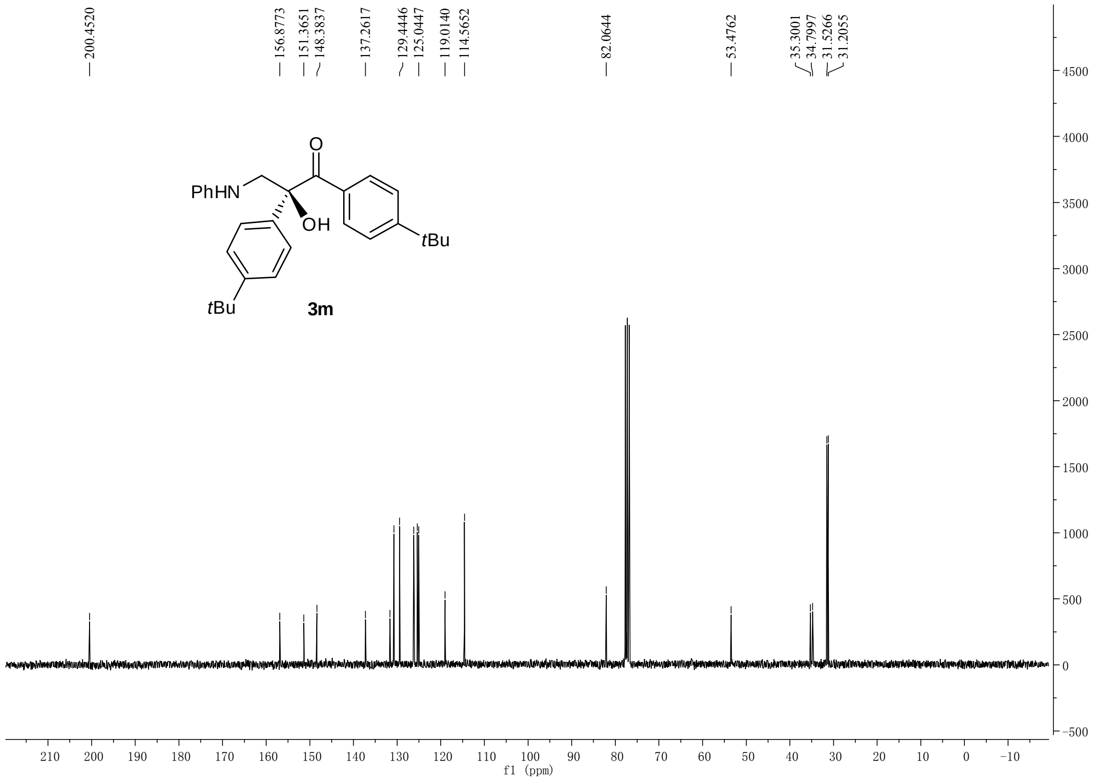
200.4520
156.8773
151.3651
148.3837
137.2617
129.4446
125.0447
119.0140
114.5652
82.0644
53.4762
35.3001
34.7997
31.5266
31.2055
PhHN
O
OH
tBu
tBu
3m
f1 (ppm)

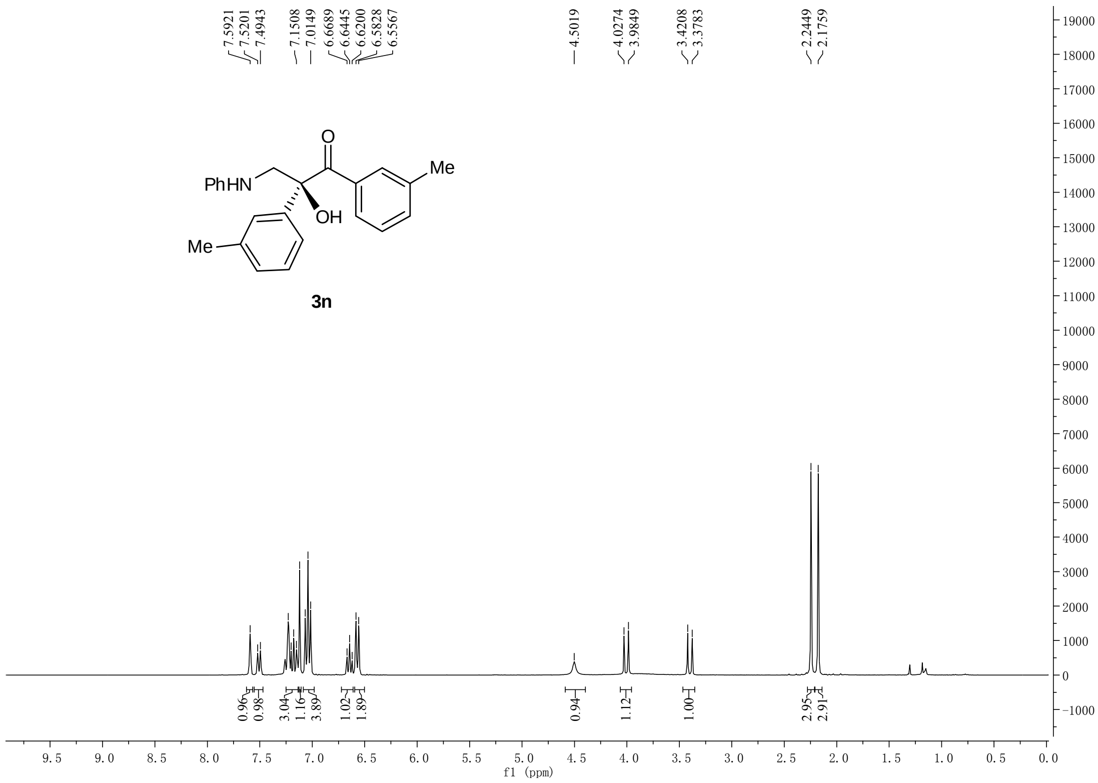
7.5921
7.5201
7.4943
7.1508
7.0149
6.6689
6.6445
6.6200
6.5828
6.5567
4.5019
4.0274
3.9849
3.4208
3.3783
2.2449
2.1759
PhHN
O
OH
Me
Me
3n
0.96
0.98
3.04
1.16
3.89
1.02
1.89
0.94
1.12
1.00
2.95
2.91
f1 (ppm)

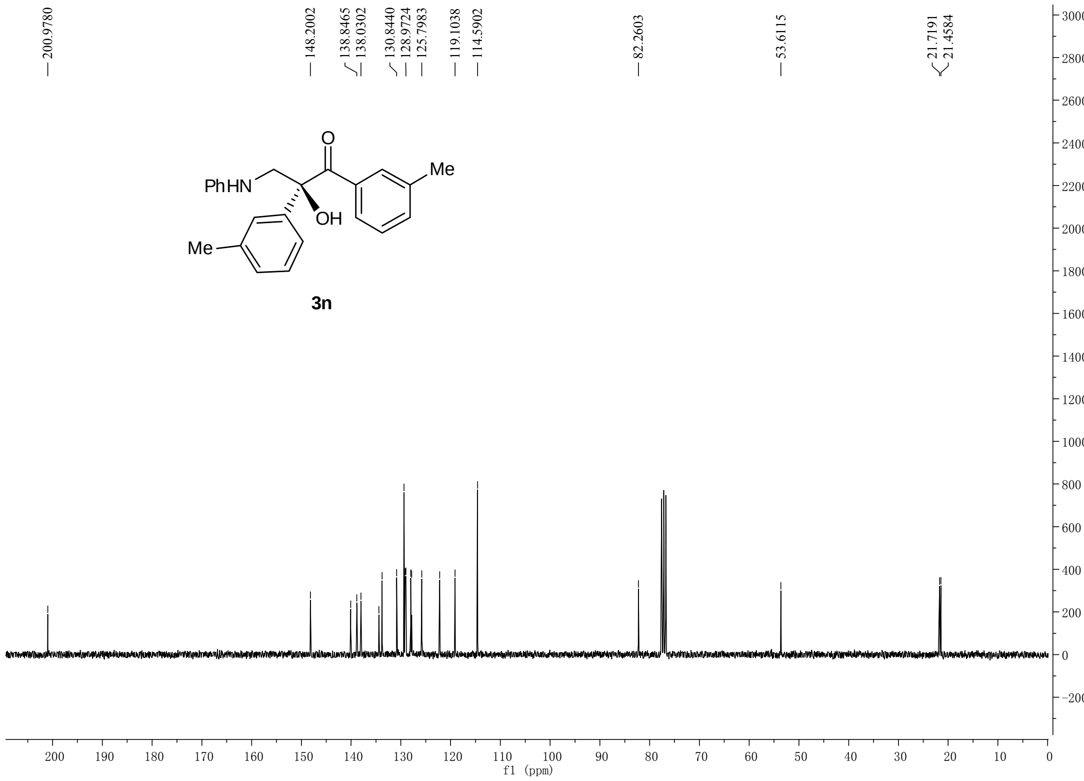
200.9780
148.2002
138.8465
138.0302
130.8440
128.9724
125.7983
119.1038
114.5902
82.2603
53.6115
21.7191
21.4584
PhHN
O
OH
Me
Me
3n
f1 (ppm)

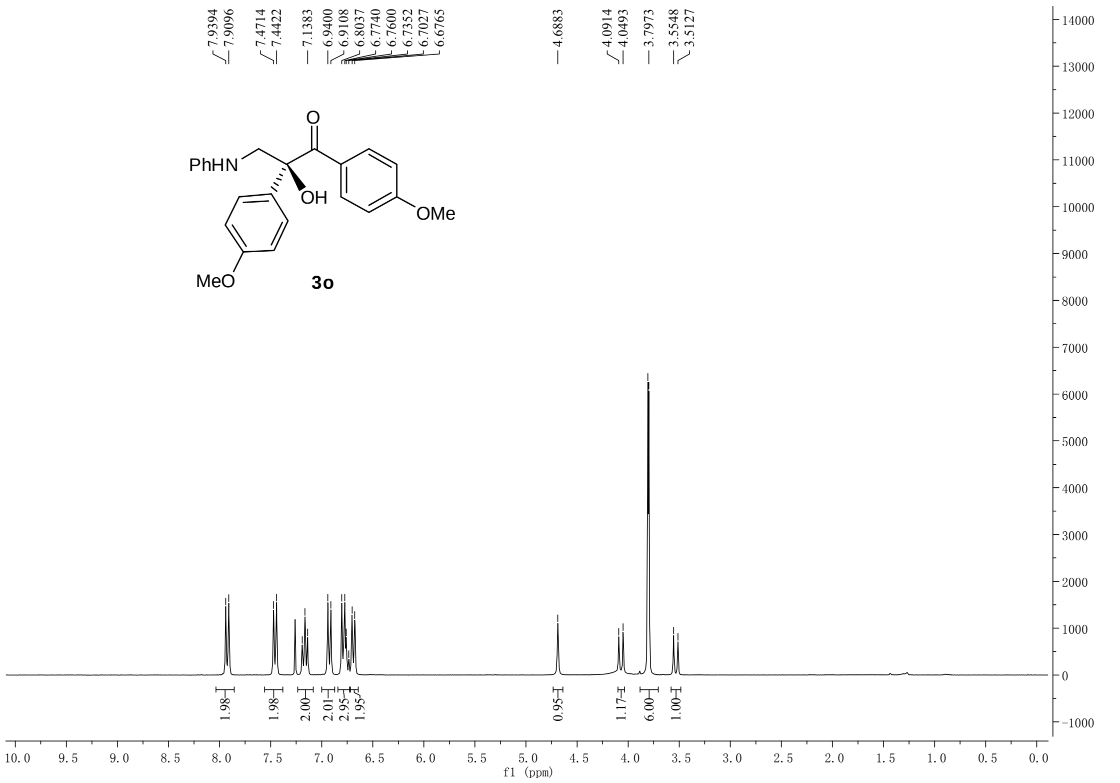
7.9394
7.9096
7.4714
7.4422
7.1383
6.9400
6.9108
6.8037
6.7740
6.7600
6.7352
6.7027
6.6765
4.6883
4.0914
4.0493
3.7973
3.5548
3.5127
PhHN
O
OH
OMe
MeO
3o
1.98
1.98
2.00
2.01
2.95
1.95
0.95
1.17
6.00
1.00
f1 (ppm)

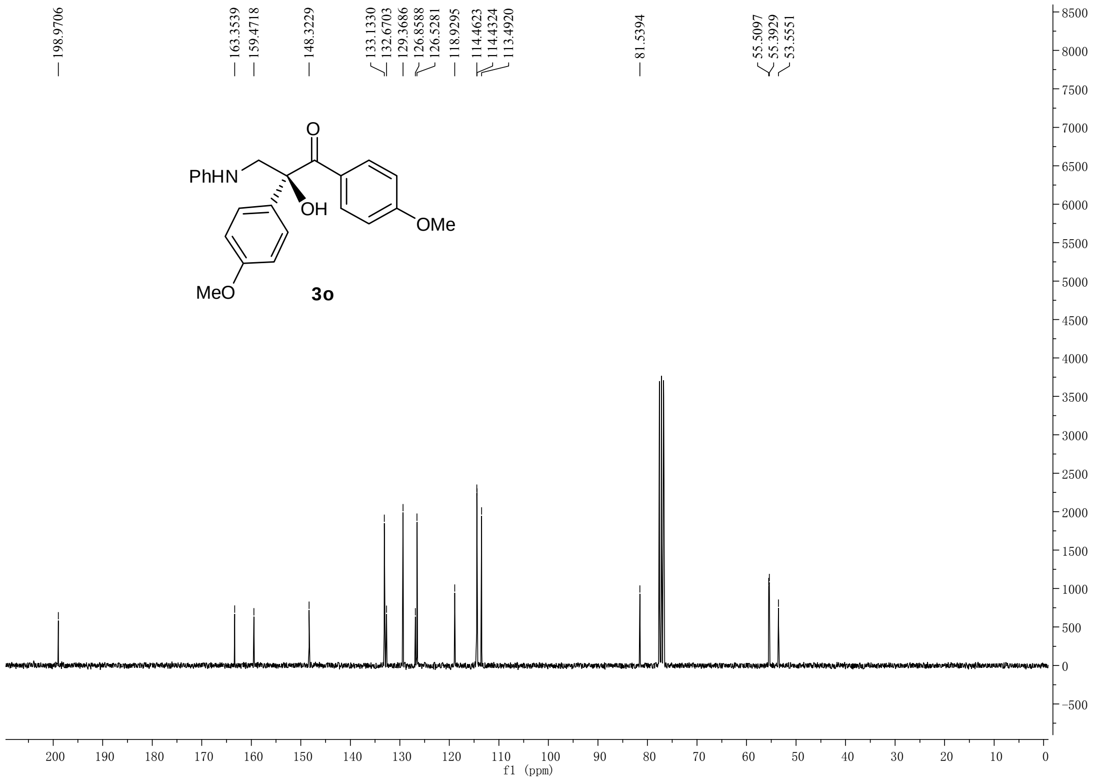
198.9706
163.3539
159.4718
148.3229
133.1330
132.6703
129.3686
126.8588
126.5281
118.9295
114.4623
114.4324
113.4920
81.5394
55.5097
55.3929
53.5551
PhHN
O
OH
OMe
MeO
3o
f1 (ppm)

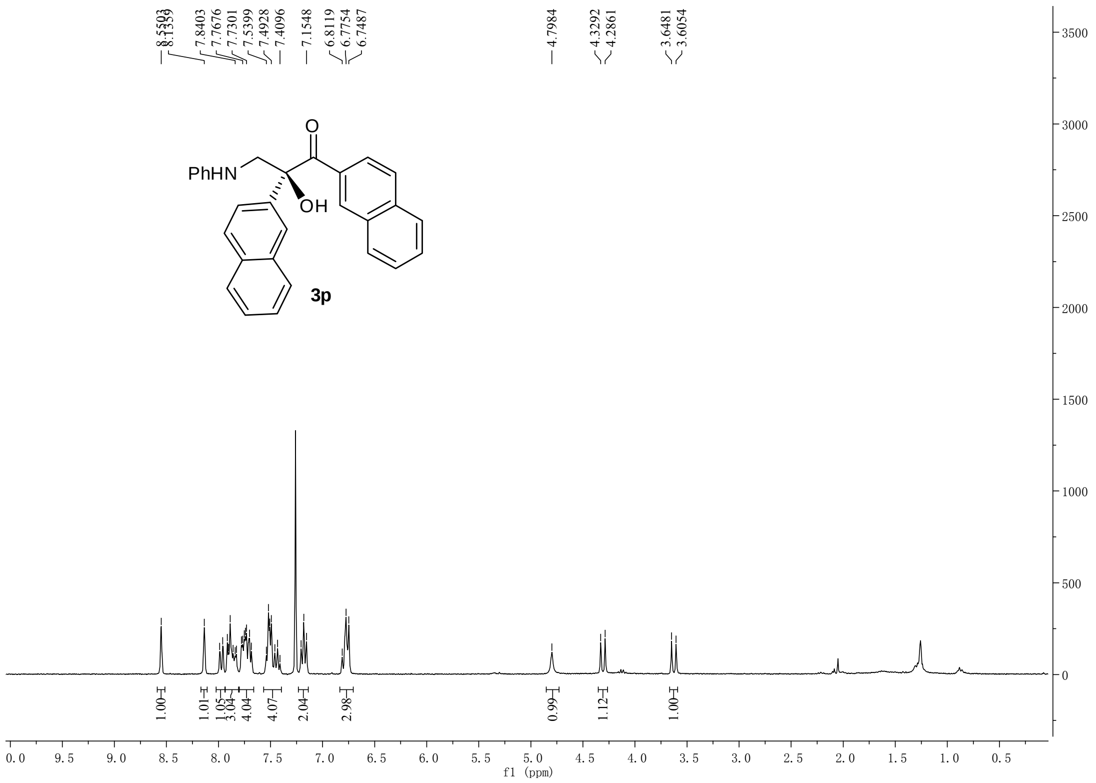

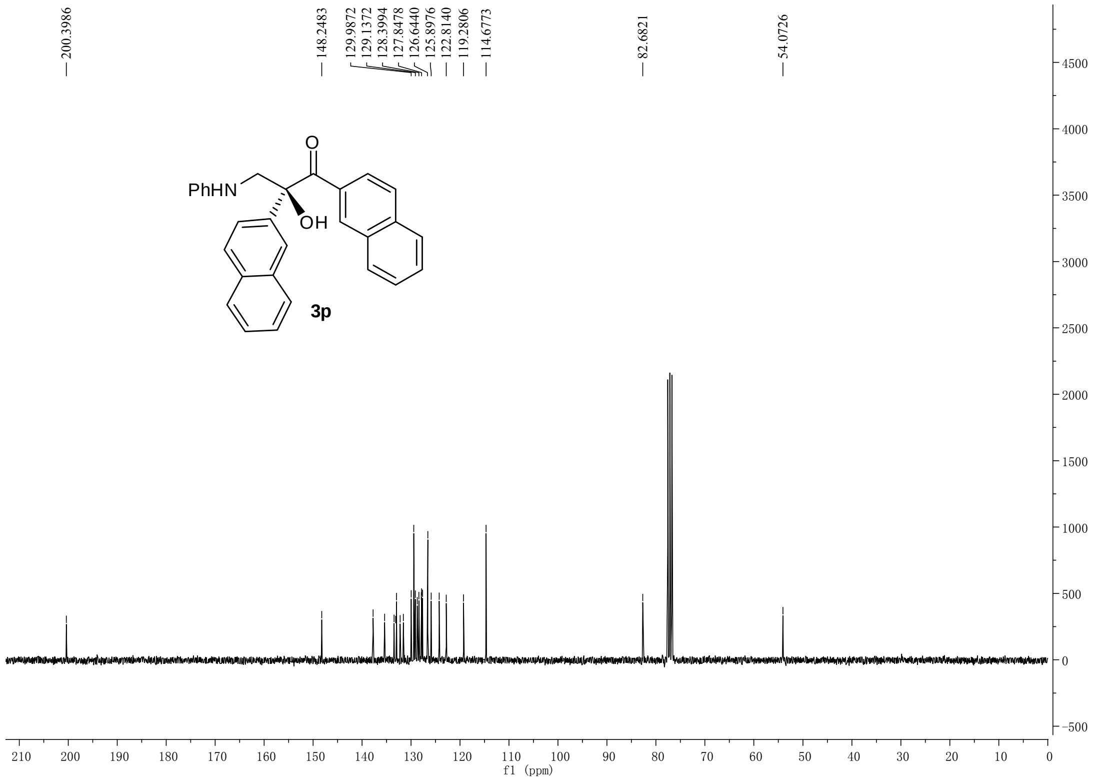

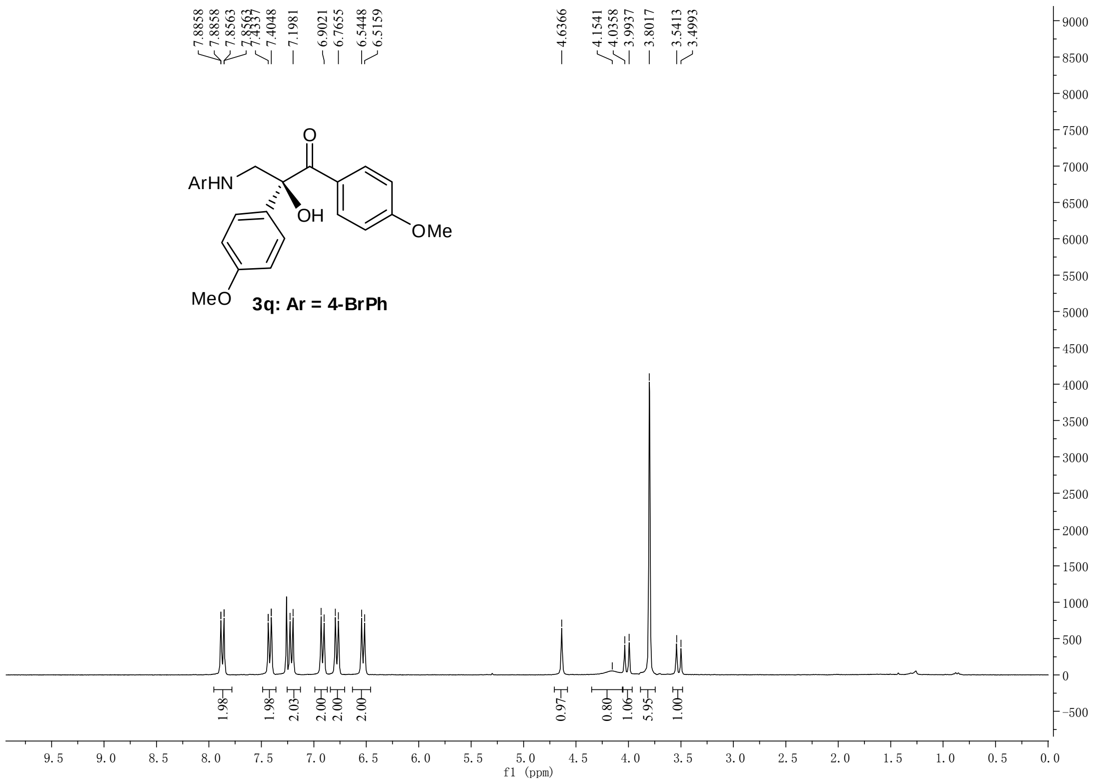
7.8858
7.8858
7.8563
7.8563
7.4337
7.4048
7.1981
6.9021
6.7655
6.5448
6.5159
4.6366
4.1541
4.0358
3.9937
3.8017
3.5413
3.4993
ArHN
O
OH
OMe
MeO
3q: Ar = 4-BrPh
1.98
1.98
2.03
2.00
2.00
2.00
0.97
0.80
1.06
5.95
1.00
f1 (ppm)

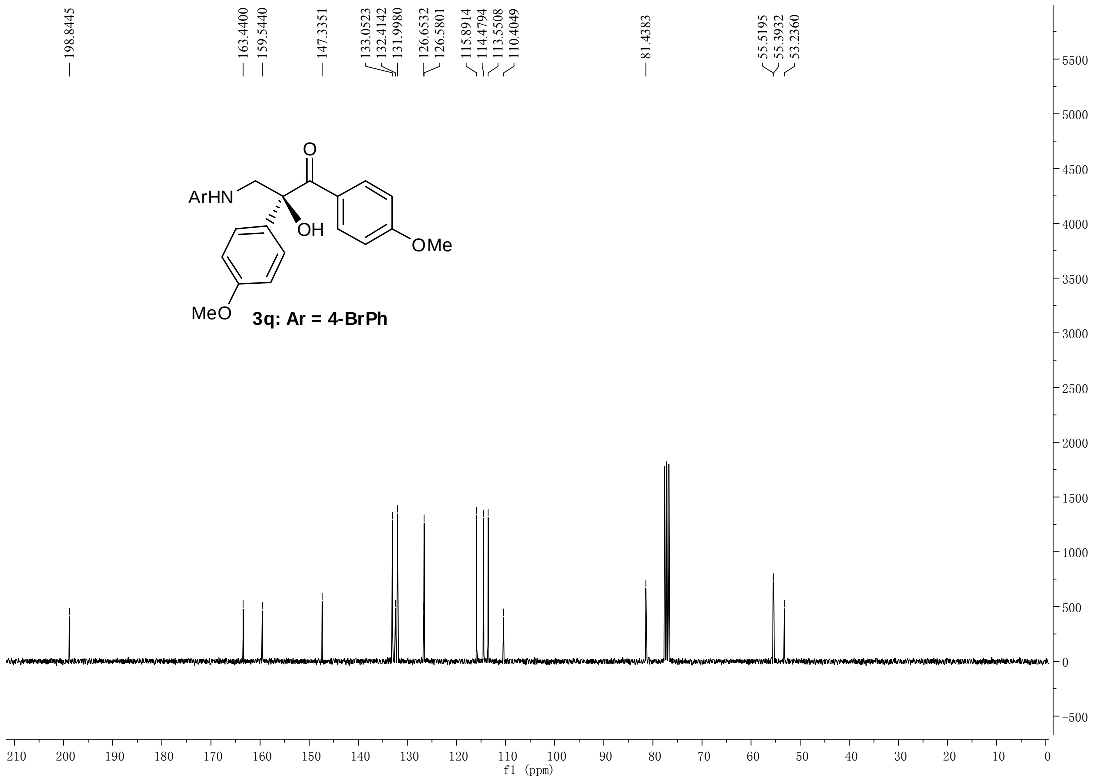
198.8445
163.4400
159.5440
147.3351
133.0523
132.4142
131.9980
126.6532
126.5801
115.8914
114.4794
113.5508
110.4049
81.4383
55.5195
55.3932
53.2360
ArHN
O
OH
OMe
MeO
3q: Ar = 4-BrPh
f1 (ppm)

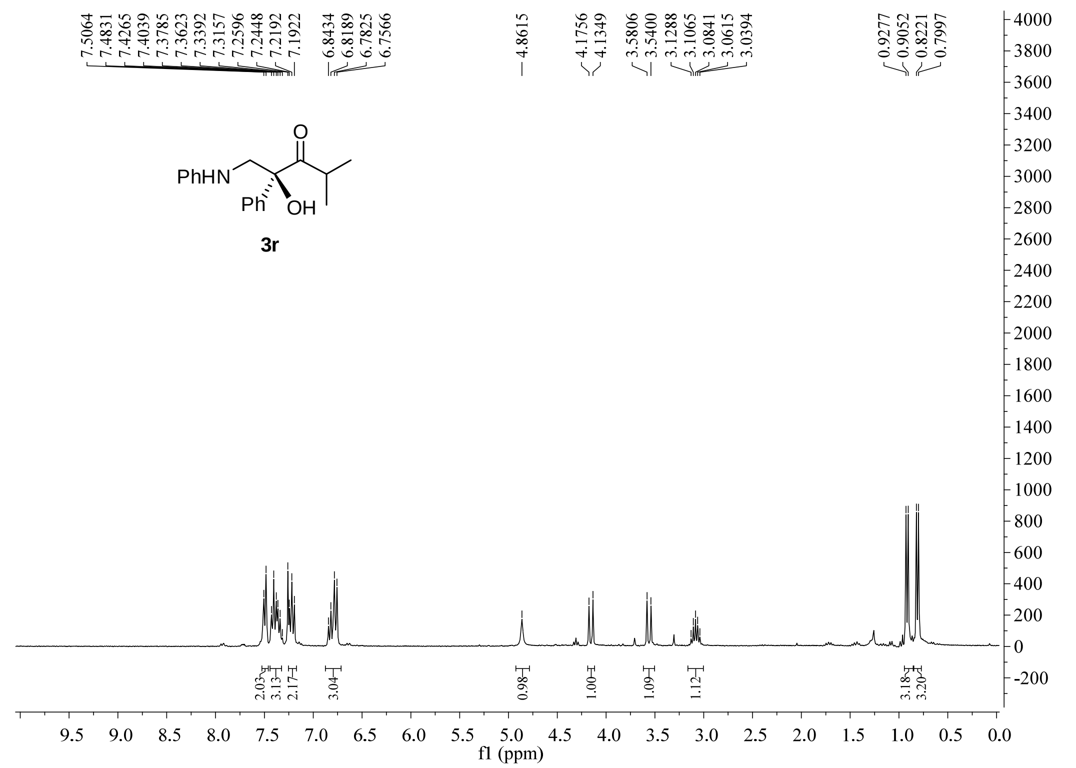
7.5064
7.4831
7.4265
7.4039
7.3785
7.3623
7.3392
7.3157
7.2596
7.2448
7.2192
7.1922
6.8434
6.8189
6.7825
6.7566
4.8615
4.1756
4.1349
3.5806
3.5400
3.1288
3.1065
3.0841
3.0615
3.0394
0.9277
0.9052
0.8221
0.7997
PhHN
O
Ph
OH
3r
2.03
3.13
2.17
3.04
0.98
1.00
1.09
1.12
3.18
3.20
f1 (ppm)

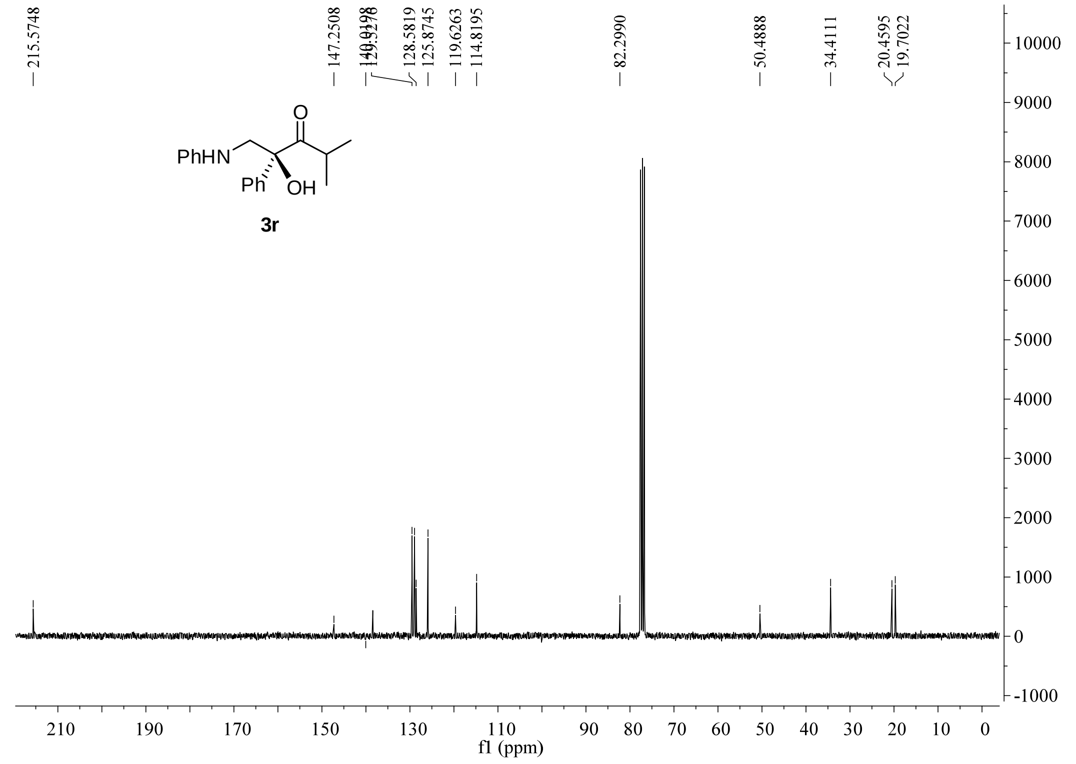
215.5748
147.2508
128.5819
125.8745
119.6263
114.8195
82.2990
50.4888
34.4111
20.4595
19.7022
PhHN
O
Ph
OH
3r
f1 (ppm)

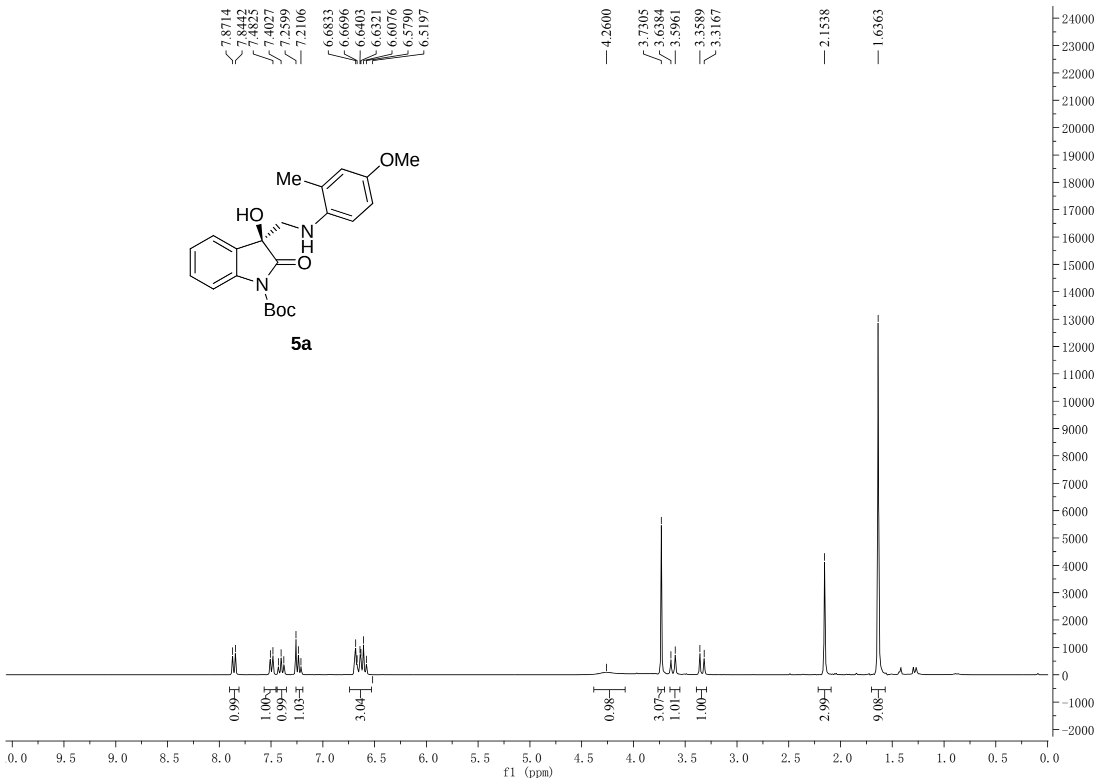
7.8714
7.8442
7.4825
7.4027
7.2599
7.2106
6.6833
6.6696
6.6403
6.6321
6.6076
6.5790
6.5197
4.2600
3.7305
3.6384
3.5961
3.3589
3.3167
2.1538
1.6363
OMe
Me
HO
N
H
O
N
Boc
5a
0.99
1.00
0.99
1.03
3.04
0.98
3.07
1.01
1.00
2.99
9.08
f1 (ppm)

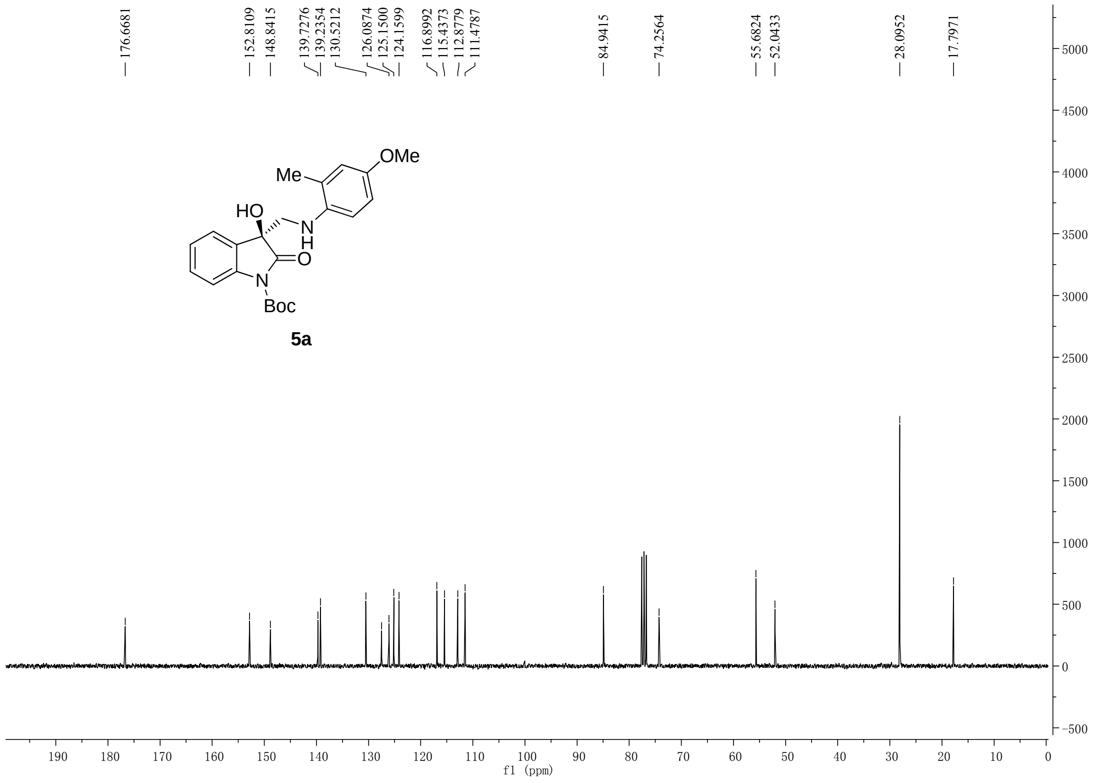
176.6681
152.8109
148.8415
139.7276
139.2354
130.5212
126.0874
125.1500
124.1599
116.8992
115.4373
112.8779
111.4787
84.9415
74.2564
55.6824
52.0433
28.0952
17.7971
OMe
Me
HO
N
H
O
N
Boc
5a
f1 (ppm)

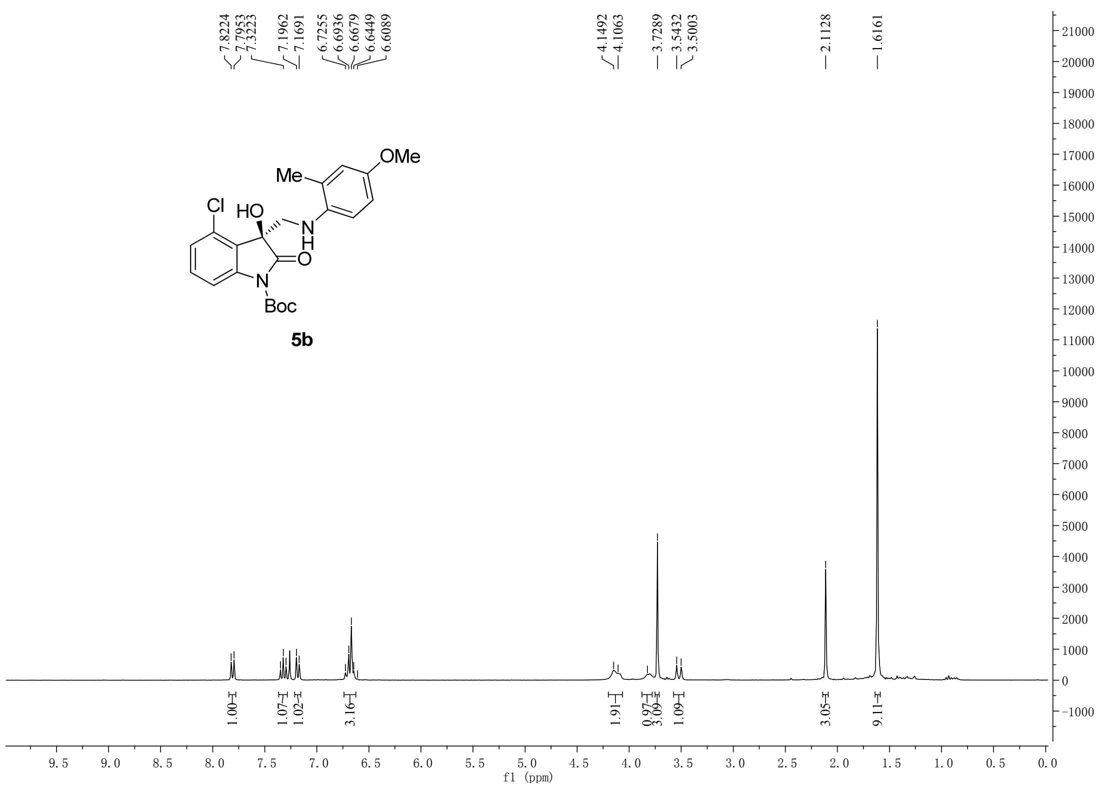
7.8224
7.7953
7.3223
7.1962
7.1691
6.7255
6.6936
6.6679
6.6449
6.6089
4.1492
4.1063
3.7289
3.5432
3.5003
2.1128
1.6161
OMe
Me
Cl
HO
N
H
O
N
Boc
5b
1.00
1.07
1.02
3.16
1.91
0.97
3.09
1.09
3.05
9.11
f1 (ppm)

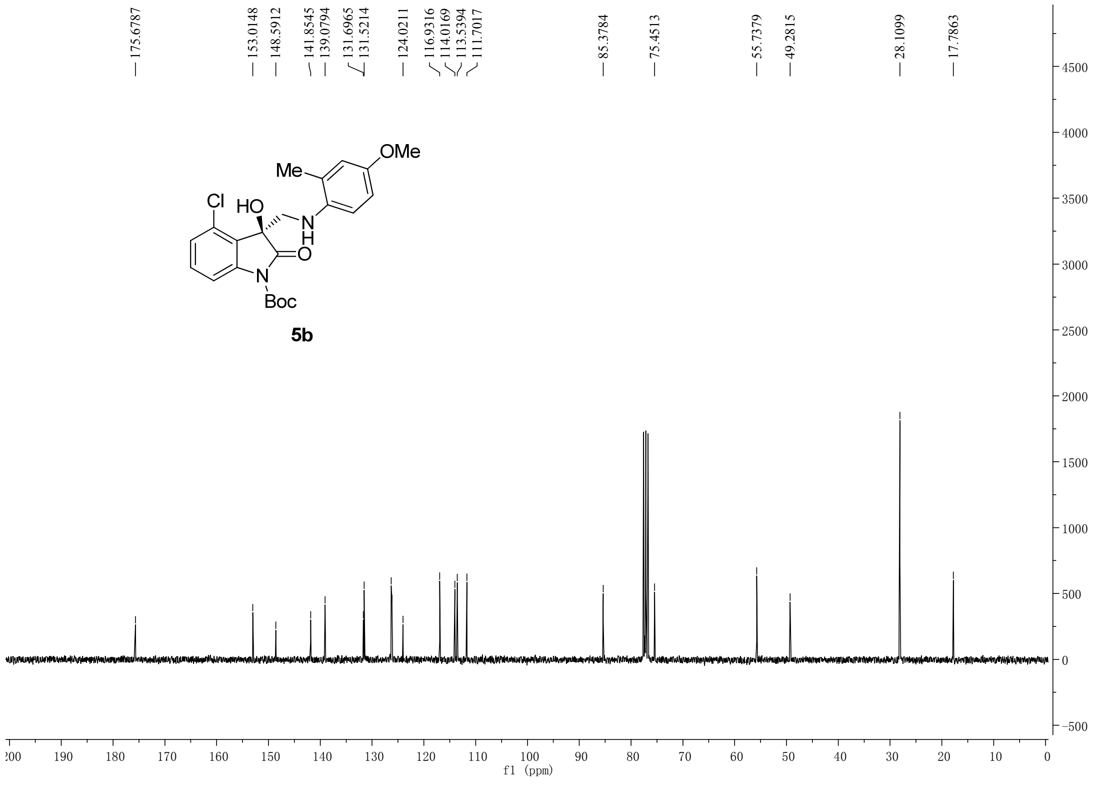
175.6787
153.0148
148.5912
141.8545
139.0794
131.6965
131.5214
124.0211
116.9316
114.0169
113.5394
111.7017
85.3784
75.4513
55.7379
49.2815
28.1099
17.7863
OMe
Me
Cl
HO
N
H
O
N
Boc
5b
f1 (ppm)

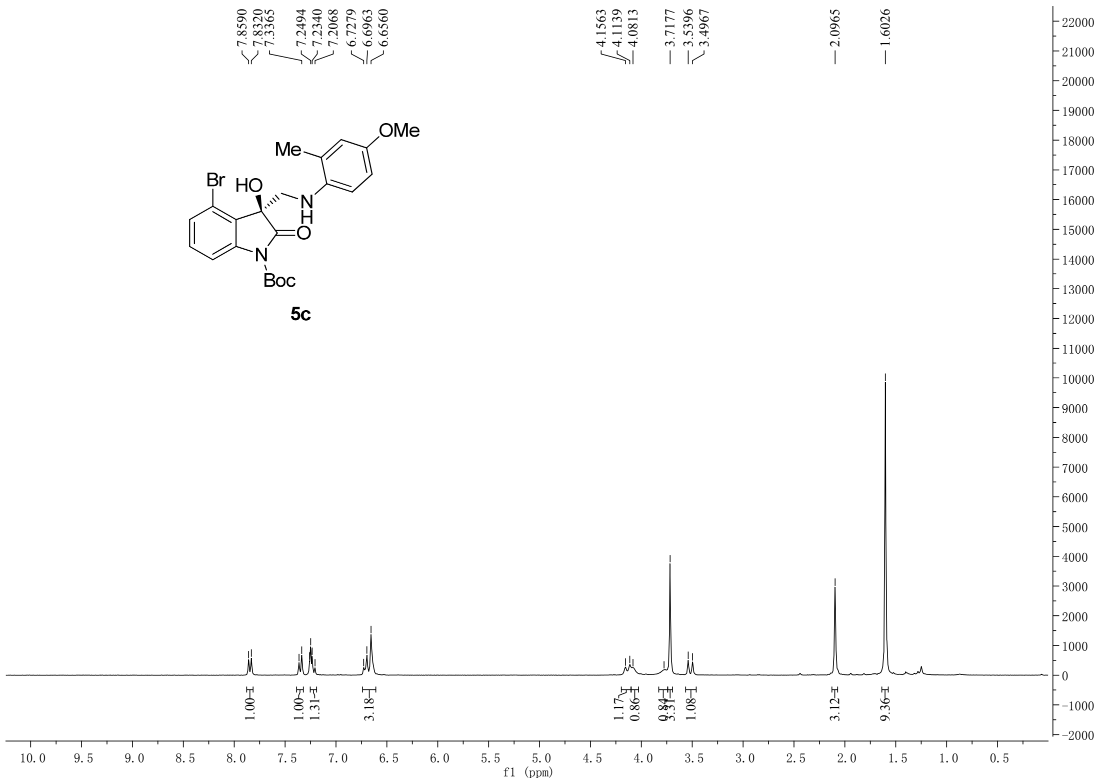
7.8590
7.8320
7.3365
7.2494
7.2340
7.2068
6.7279
6.6963
6.6560
4.1563
4.1139
4.0813
3.7177
3.5396
3.4967
2.0965
1.6026
OMe
Me
Br
HO
N
H
O
N
Boc
5c
1.00
1.00
1.31
3.18
1.17
0.86
0.84
3.31
1.08
3.12
9.36
f1 (ppm)

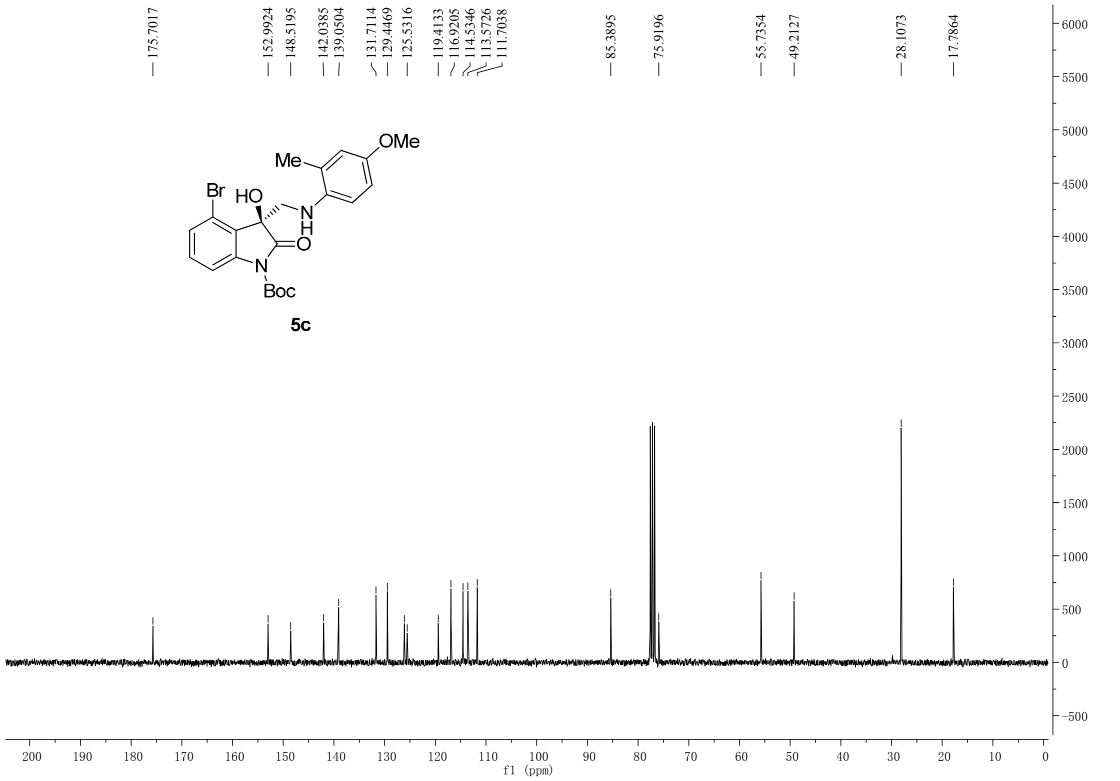
175.7017
152.9924
148.5195
142.0385
139.0504
131.7114
129.4469
125.5316
119.4133
116.9205
114.5346
113.5726
111.7038
85.3895
75.9196
55.7354
49.2127
28.1073
17.7864
OMe
Me
Br
HO
N
H
O
N
Boc
5c
f1 (ppm)

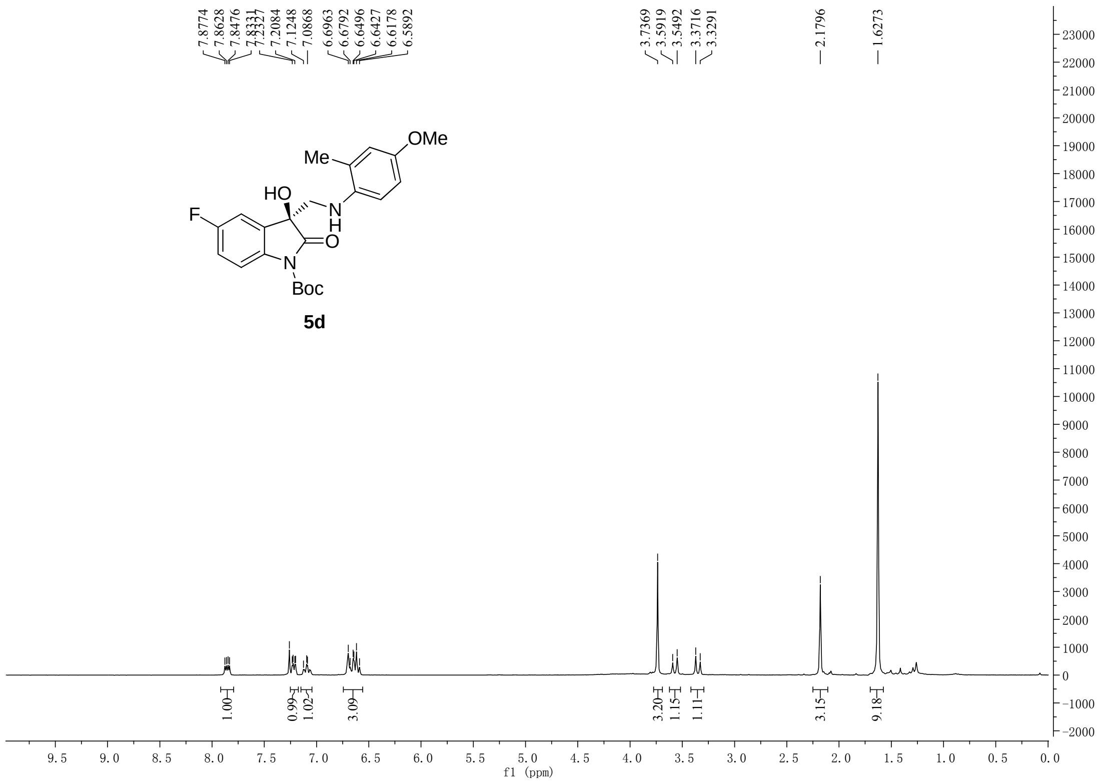
7.8774
7.8628
7.8476
7.8331
7.2327
7.2084
7.1248
7.0868
6.6963
6.6792
6.6496
6.6427
6.6178
6.5892
3.7369
3.5919
3.5492
3.3716
3.3291
2.1796
1.6273
OMe
Me
HO
N
H
F
O
N
Boc
5d
1.00
0.99
1.02
3.09
3.20
1.15
1.11
3.15
9.18
f1 (ppm)

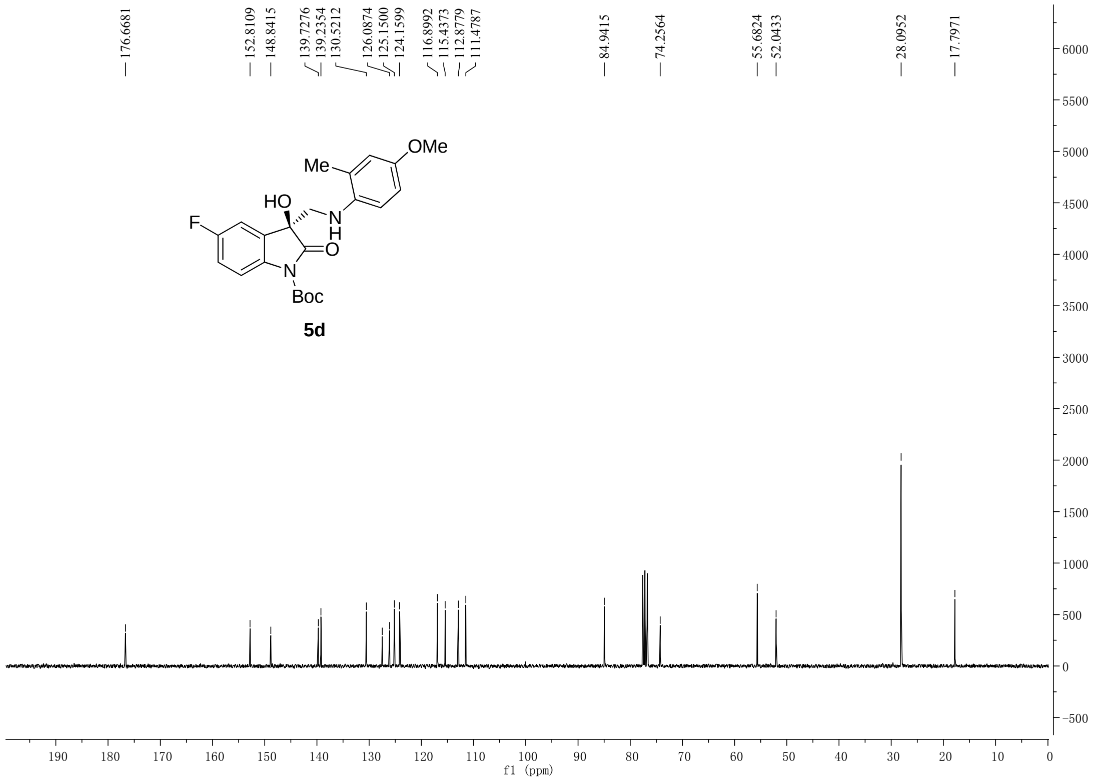
176.6681
152.8109
148.8415
139.7276
139.2354
130.5212
126.0874
125.1500
124.1599
116.8992
115.4373
112.8779
111.4787
84.9415
74.2564
55.6824
52.0433
28.0952
17.7971
OMe
Me
HO
N
H
F
O
N
Boc
5d
f1 (ppm)

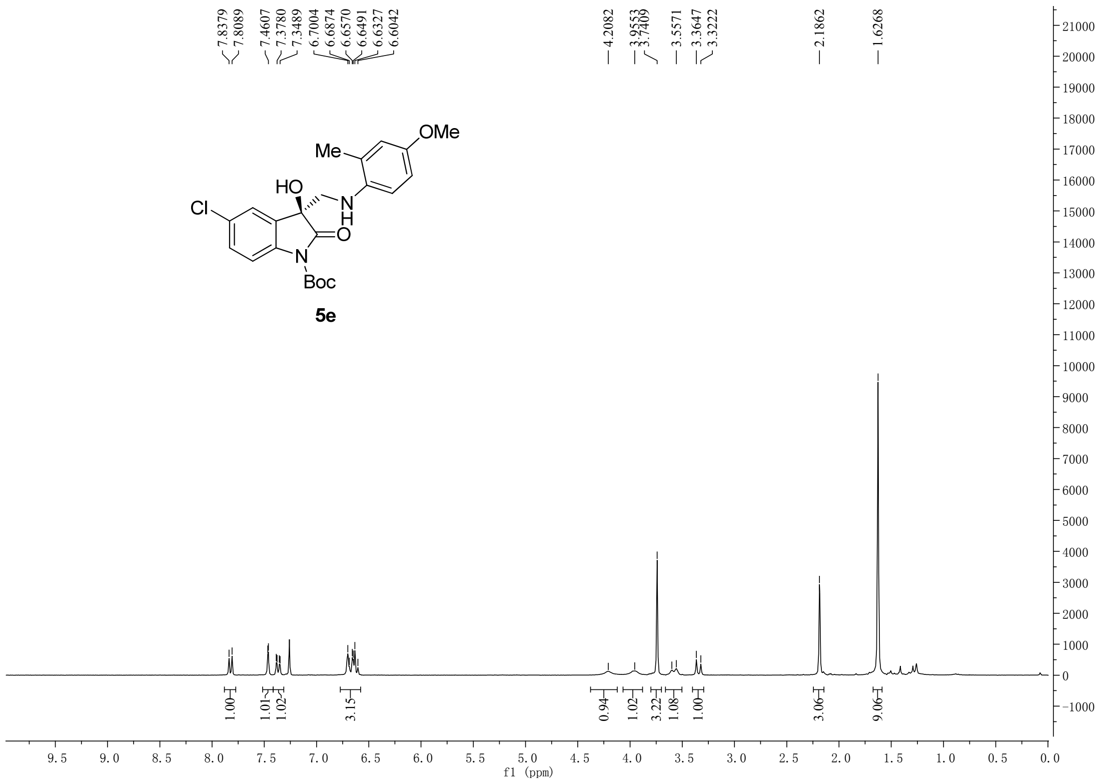
7.8379
7.8089
7.4607
7.3780
7.3489
6.7004
6.6874
6.6570
6.6491
6.6327
6.6042
4.2082
3.9553
3.7409
3.5571
3.3647
3.3222
2.1862
1.6268
OMe
Me
HO
N
H
Cl
O
N
Boc
5e
1.00
1.01
1.02
3.15
0.94
1.02
3.22
1.08
1.00
3.06
9.06
f1 (ppm)

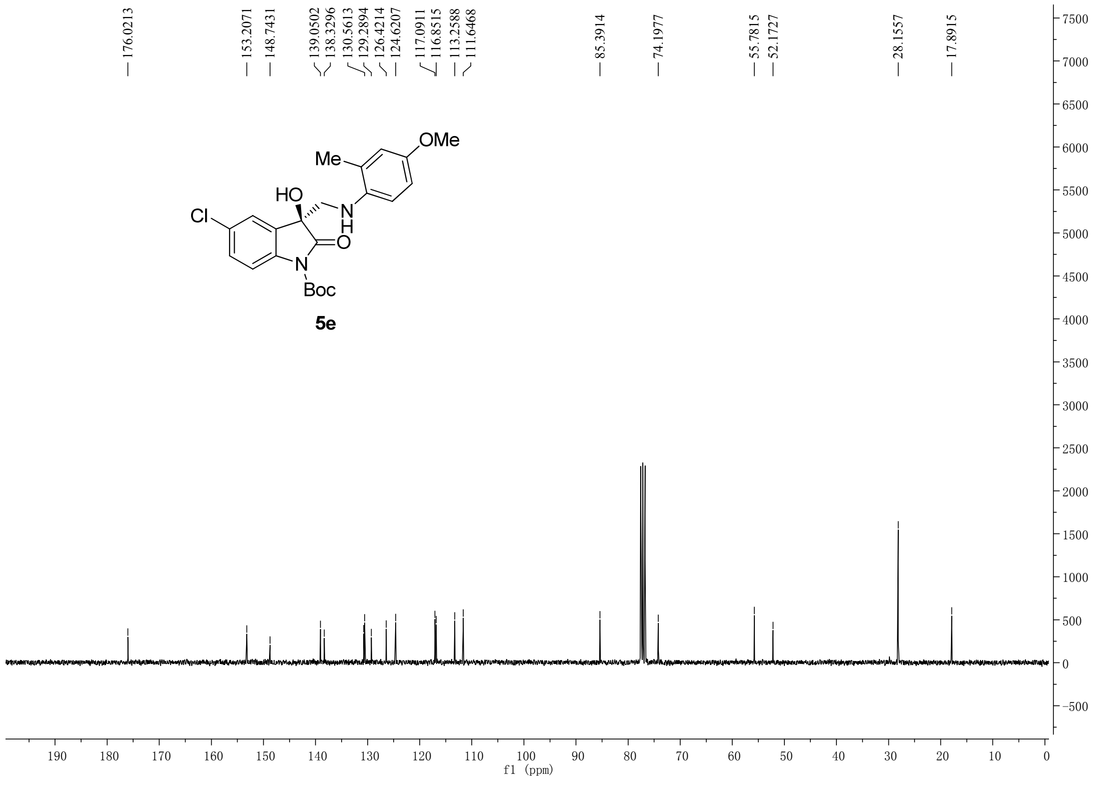
176.0213
153.2071
148.7431
139.0502
138.3296
130.5613
129.2894
126.4214
124.6207
117.0911
116.8515
113.2588
111.6468
85.3914
74.1977
55.7815
52.1727
28.1557
17.8915
OMe
Me
HO
N
H
Cl
O
N
Boc
5e
f1 (ppm)

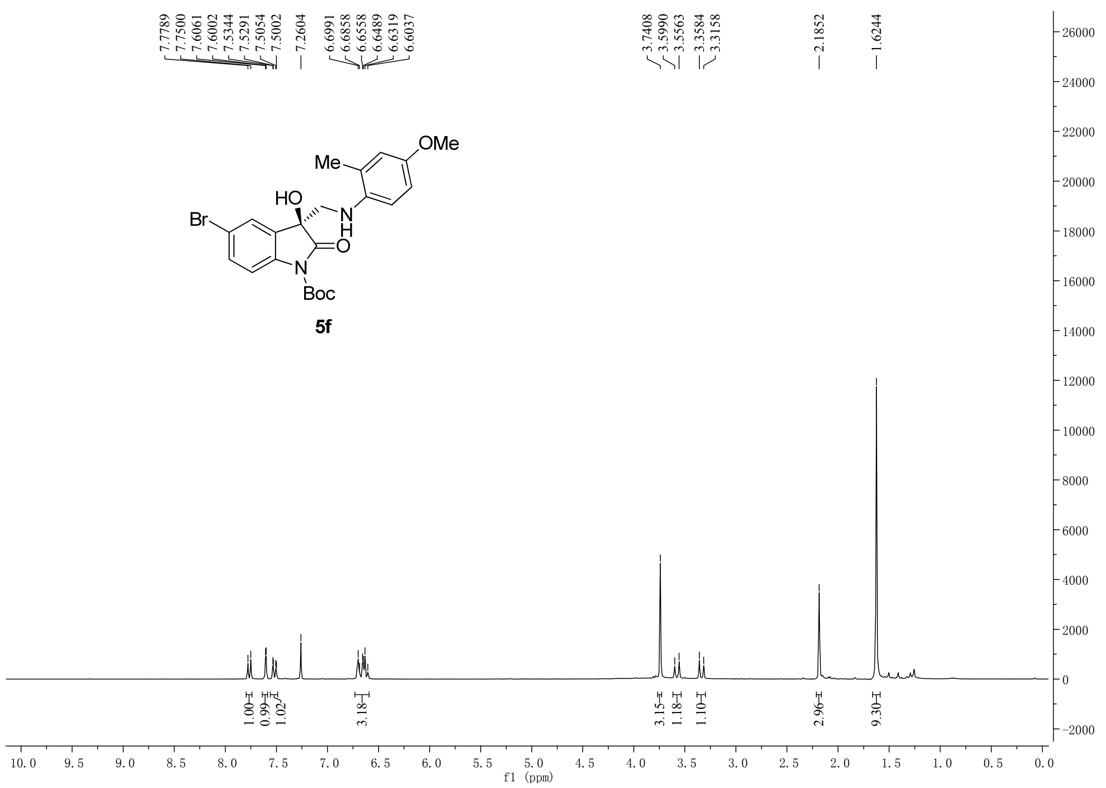
7.7789
7.7500
7.6061
7.6002
7.5344
7.5291
7.5054
7.5002
7.2604
6.6991
6.6858
6.6558
6.6489
6.6319
6.6037
3.7408
3.5990
3.5563
3.3584
3.3158
2.1852
1.6244
OMe
Me
HO
Br
N
H
O
N
Boc
5f
1.00
0.99
1.02
3.18
3.15
1.18
1.10
2.96
9.30
f1 (ppm)

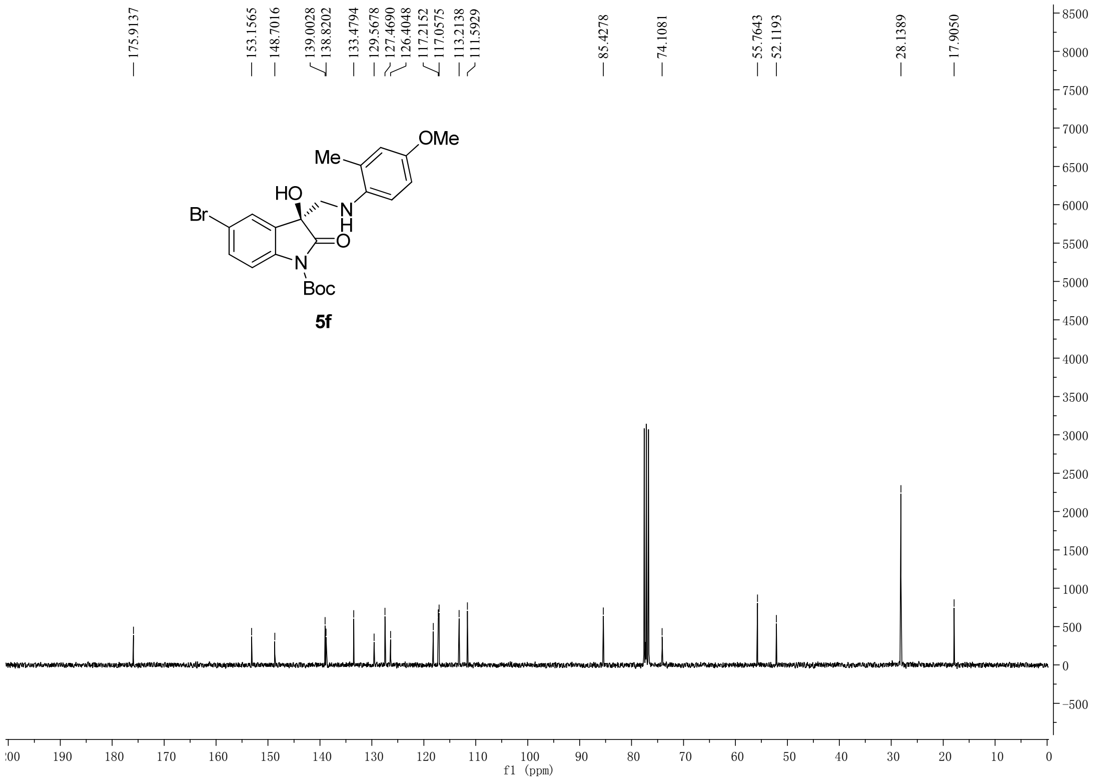
175.9137
153.1565
148.7016
139.0028
138.8202
133.4794
129.5678
127.4690
126.4048
117.2152
117.0575
113.2138
111.5929
85.4278
74.1081
55.7643
52.1193
28.1389
17.9050
OMe
Me
HO
Br
N
H
O
N
Boc
5f
f1 (ppm)

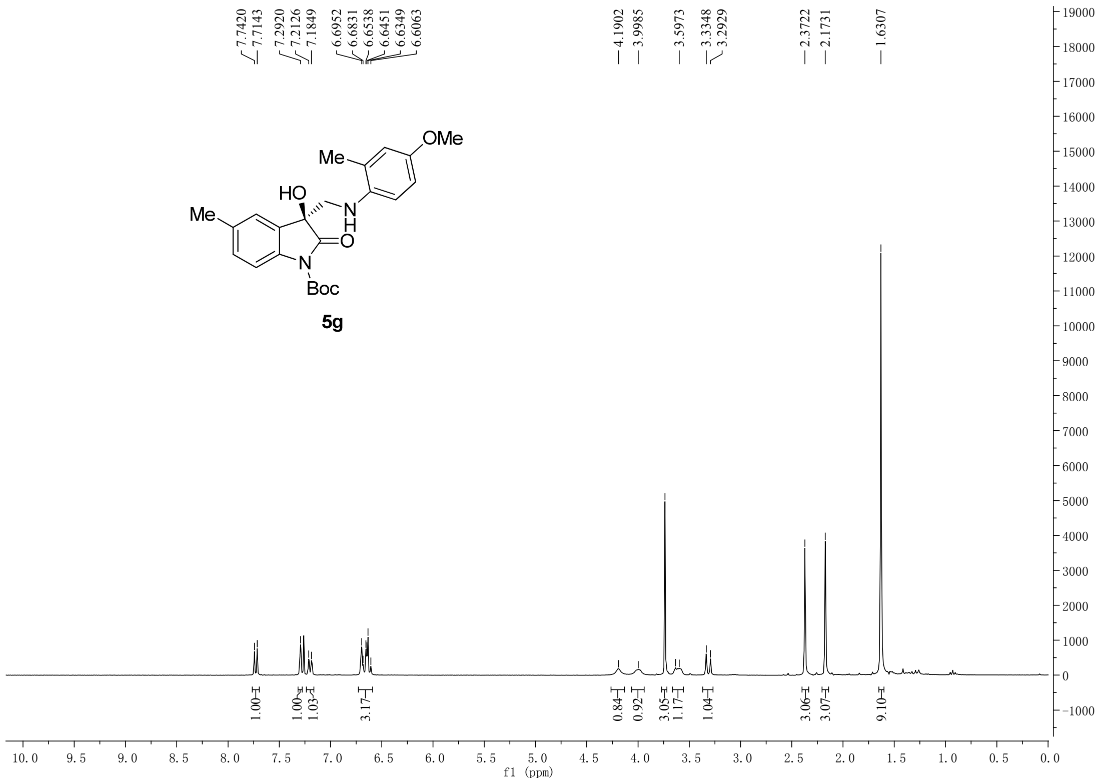
7.7420
7.7143
7.2920
7.2126
7.1849
6.6952
6.6831
6.6538
6.6451
6.6349
6.6063
4.1902
3.9985
3.5973
3.3348
3.2929
2.3722
2.1731
1.6307
OMe
Me
HO
NH
Me
N
O
Boc
5g
1.00
1.00
1.03
3.17
0.84
0.92
3.05
1.17
1.04
3.06
3.07
9.10
f1 (ppm)

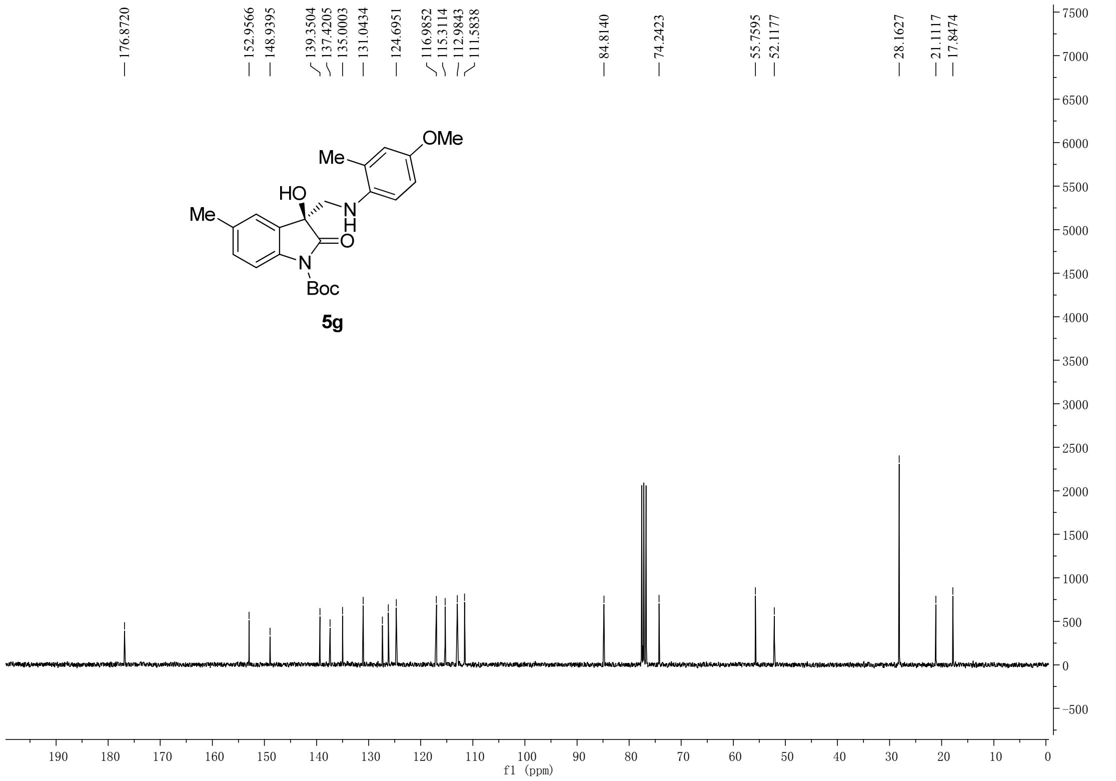
176.8720
152.9566
148.9395
139.3504
137.4205
135.0003
131.0434
124.6951
116.9852
115.3114
112.9843
111.5838
84.8140
74.2423
55.7595
52.1177
28.1627
21.1117
17.8474
OMe
Me
HO
NH
Me
N
O
Boc
5g
f1 (ppm)

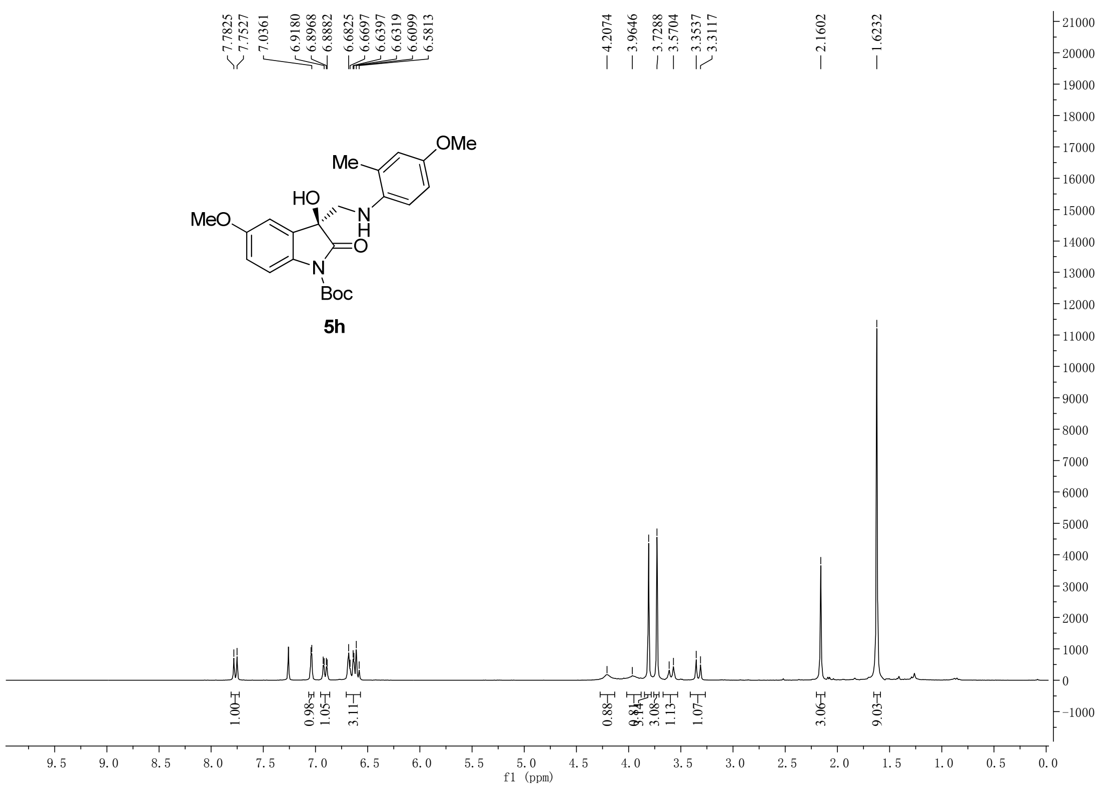
7.7825
7.7527
7.0361
6.9180
6.8968
6.8882
6.6825
6.6697
6.6397
6.6319
6.6099
6.5813
4.2074
3.9646
3.7288
3.5704
3.3537
3.3117
2.1602
1.6232
OMe
Me
HO
MeO
N
H
O
N
Boc
5h
1.00
0.98
1.05
3.11
0.88
3.08
1.13
1.07
3.06
9.03
f1 (ppm)

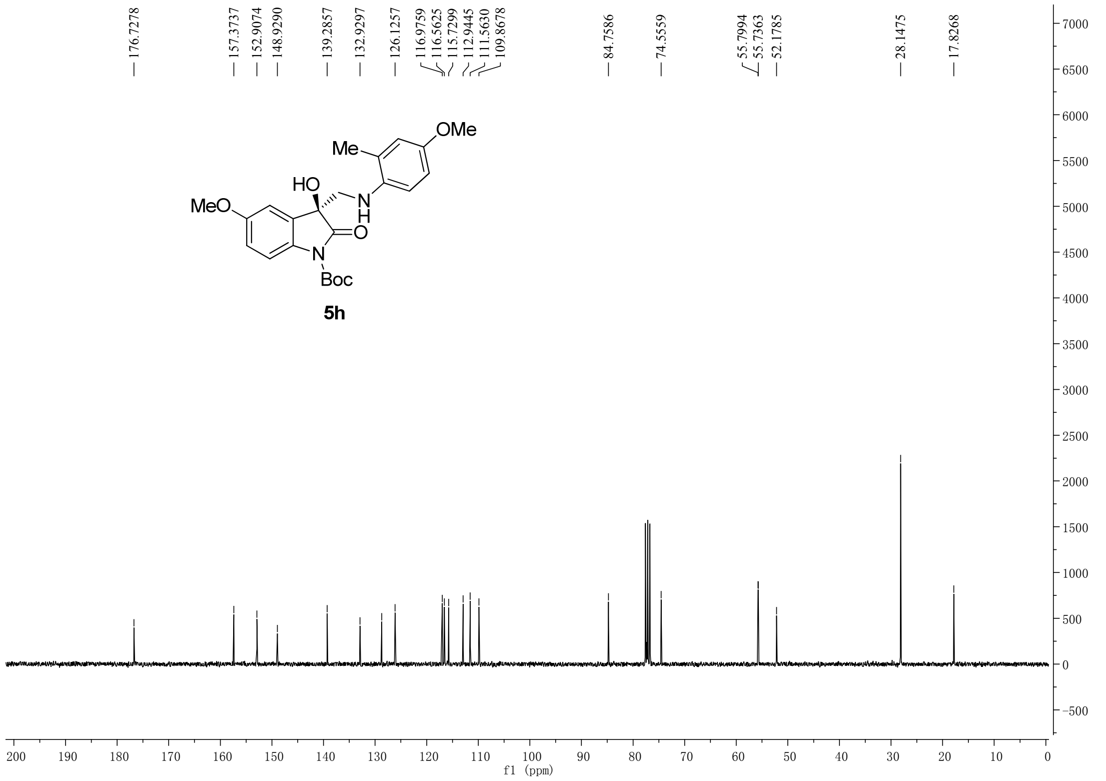
176.7278
157.3737
152.9074
148.9290
139.2857
132.9297
126.1257
116.9759
116.5625
115.7299
112.9445
111.5630
109.8678
84.7586
74.5559
55.7994
55.7363
52.1785
28.1475
17.8268
OMe
Me
HO
MeO
N
H
O
N
Boc
5h
f1 (ppm)

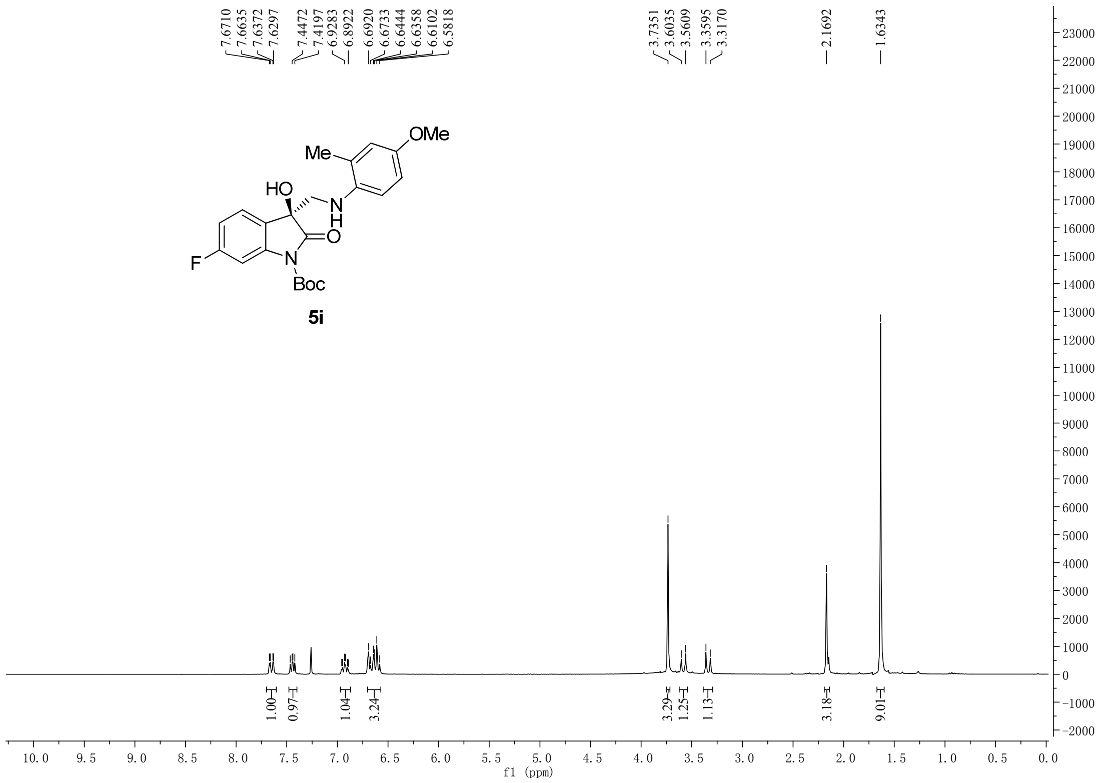
7.6710
7.6635
7.6372
7.6297
7.4472
7.4197
6.9283
6.8922
6.6920
6.6733
6.6444
6.6358
6.6102
6.5818
3.7351
3.6035
3.5609
3.3595
3.3170
2.1692
1.6343
OMe
Me
HO
N
H
O
F
N
Boc
5i
1.00
0.97
1.04
3.24
3.29
1.25
1.13
3.18
9.01
f1 (ppm)

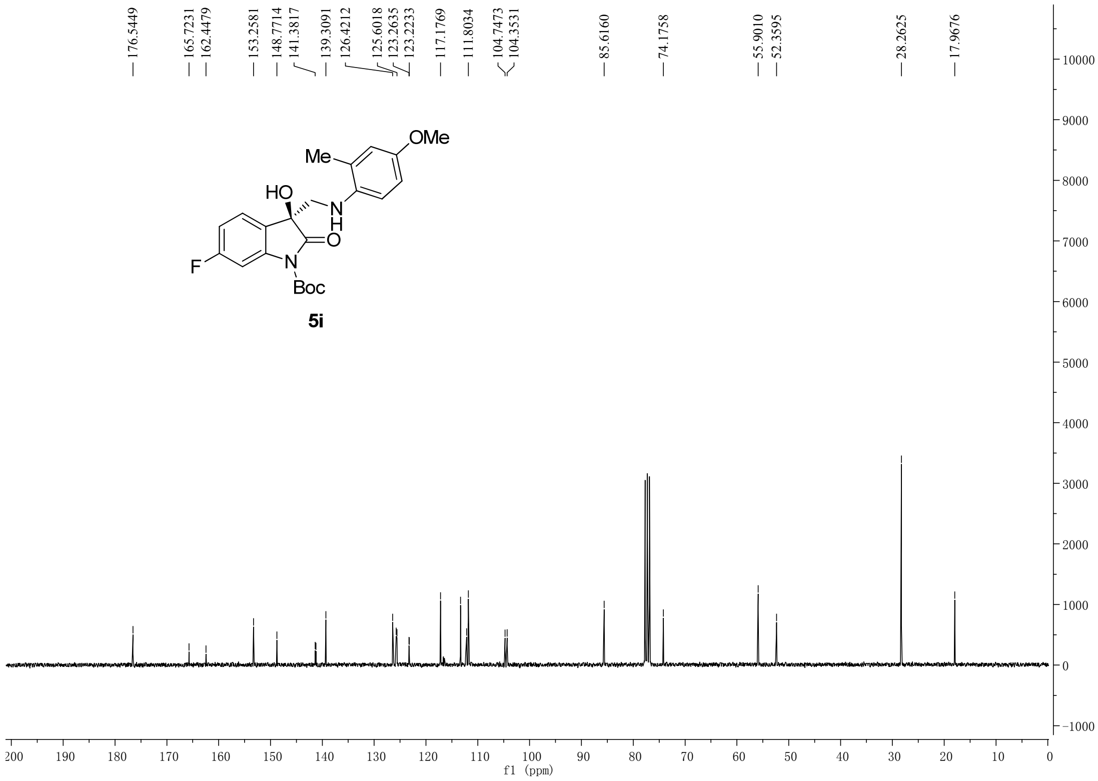
176.5449
165.7231
162.4479
153.2581
148.7714
141.3817
139.3091
126.4212
125.6018
123.2635
123.2233
117.1769
111.8034
104.7473
104.3531
85.6160
74.1758
55.9010
52.3595
28.2625
17.9676
OMe
Me
HO
N
H
O
F
N
Boc
5i
f1 (ppm)

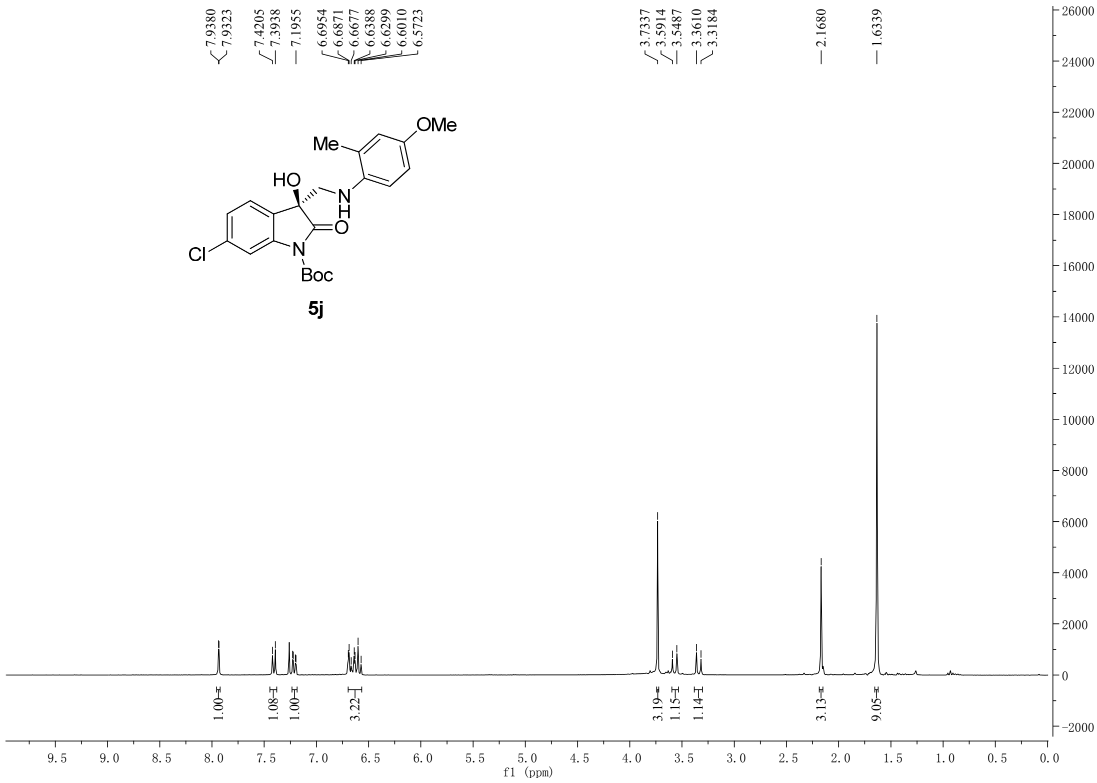
7.9380
7.9323
7.4205
7.3938
7.1955
6.6954
6.6871
6.6677
6.6388
6.6299
6.6010
6.5723
3.7337
3.5914
3.5487
3.3610
3.3184
2.1680
1.6339
OMe
Me
HO
N
H
O
Cl
N
Boc
5j
1.00
1.08
1.00
3.22
3.19
1.15
1.14
3.13
9.05
f1 (ppm)

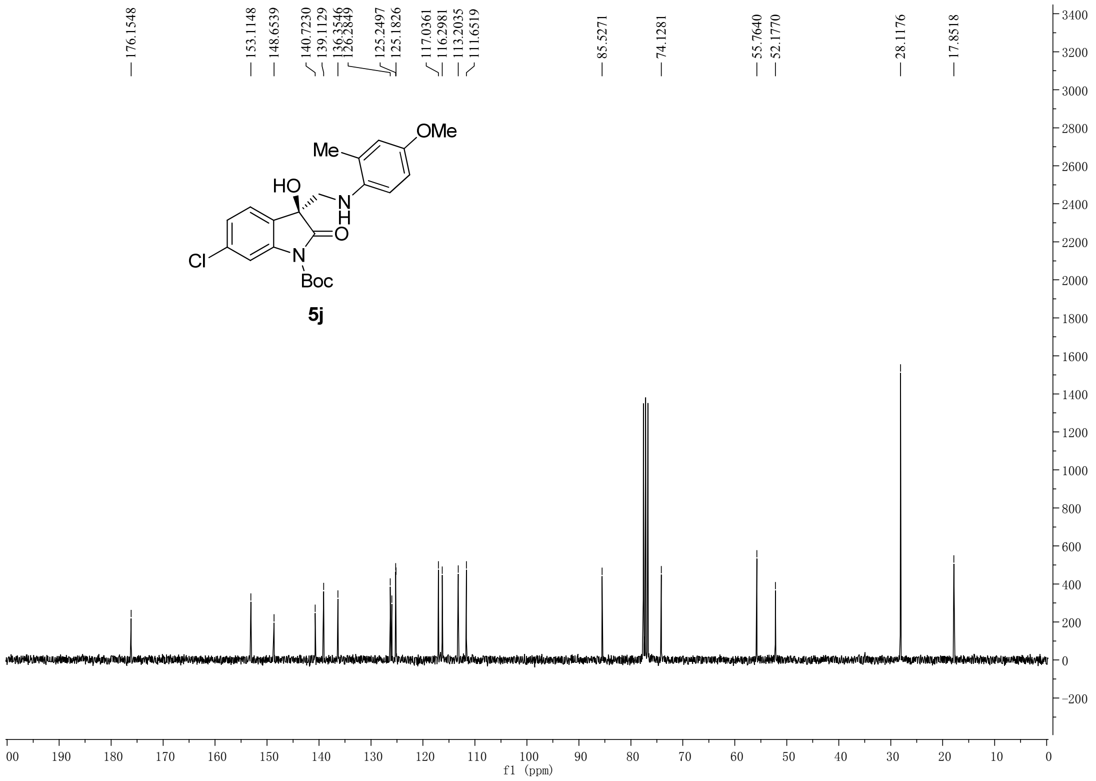
176.1548
153.1148
148.6539
140.7230
139.1129
136.3546
126.2849
125.2497
125.1826
117.0361
116.2981
113.2035
111.6519
85.5271
74.1281
55.7640
52.1770
28.1176
17.8518
OMe
Me
HO
N
H
O
Cl
N
Boc
5j
f1 (ppm)

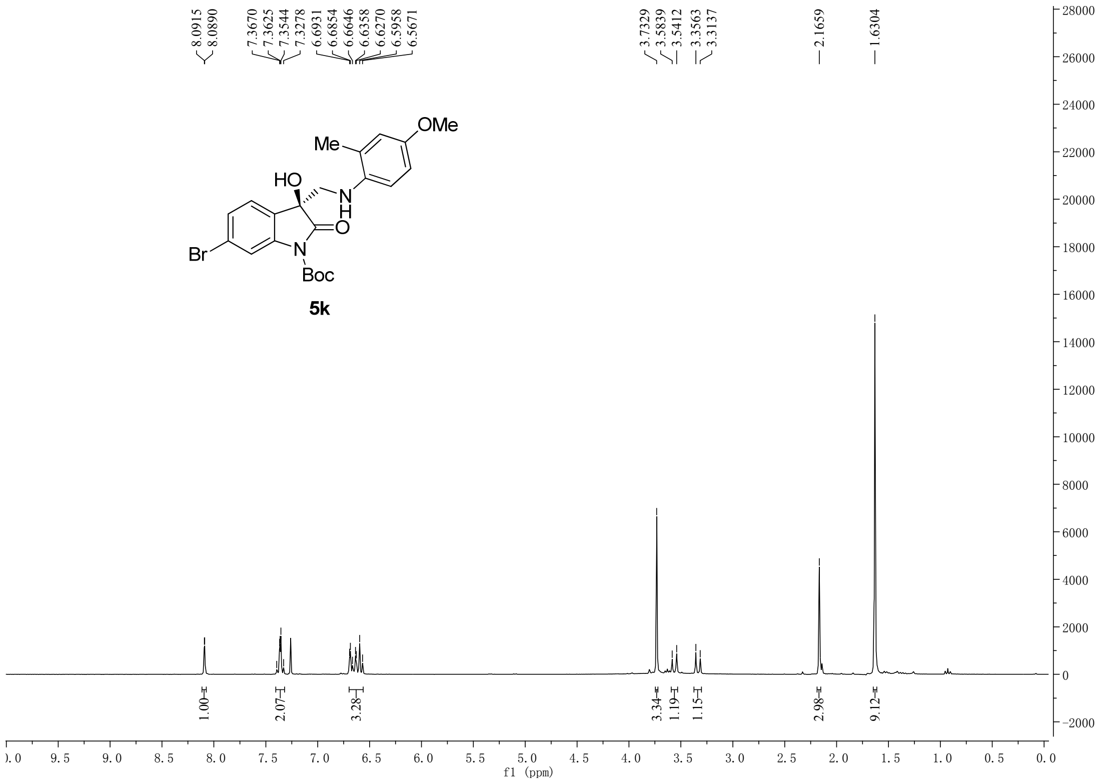
8.0915
8.0890
7.3670
7.3625
7.3544
7.3278
6.6931
6.6854
6.6646
6.6358
6.6270
6.5958
6.5671
3.7329
3.5839
3.5412
3.3563
3.3137
2.1659
1.6304
OMe
Me
HO
N
H
O
Br
N
Boc
5k
1.00
2.07
3.28
3.34
1.19
1.15
2.98
9.12
f1 (ppm)

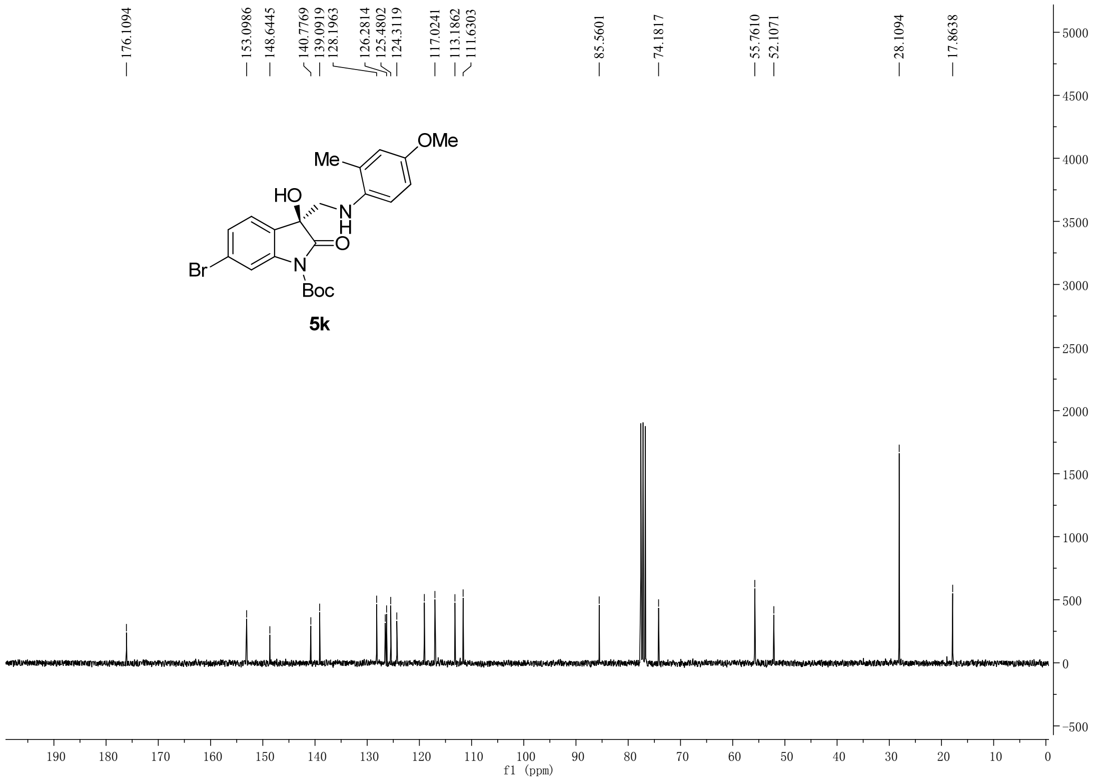
176.1094
153.0986
148.6445
140.7769
139.0919
128.1963
126.2814
125.4802
124.3119
117.0241
113.1862
111.6303
85.5601
74.1817
55.7610
52.1071
28.1094
17.8638
OMe
Me
HO
N
H
O
Br
N
Boc
5k
f1 (ppm)

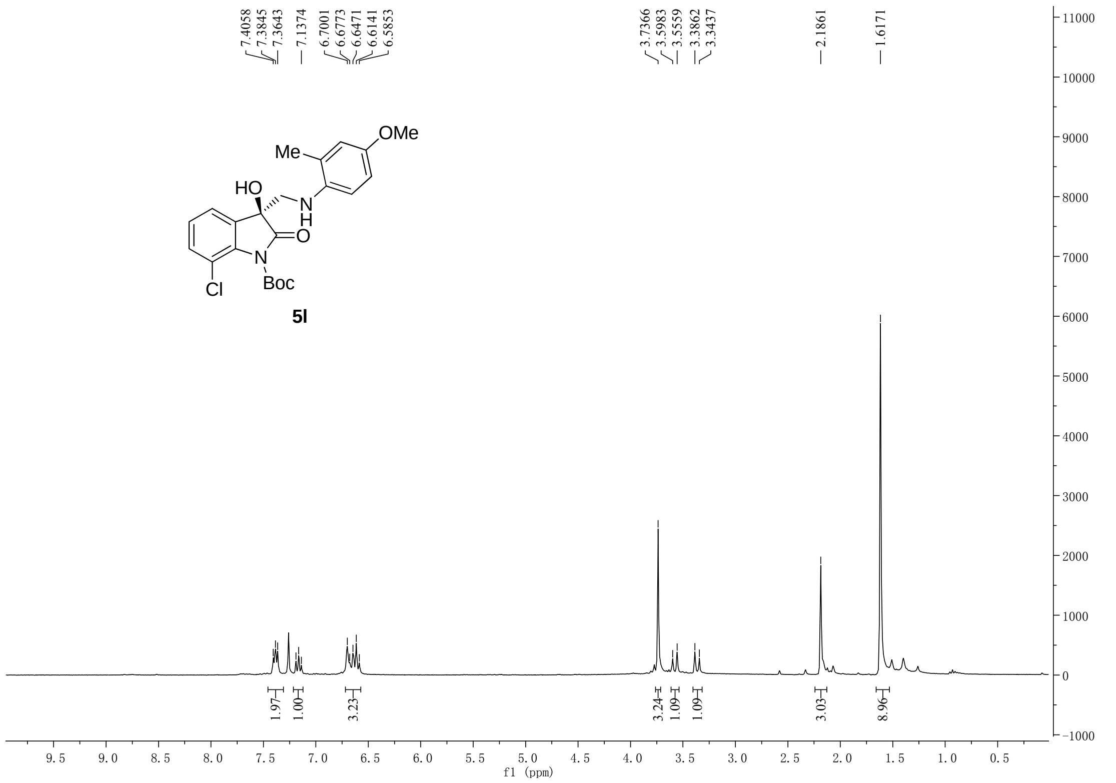
7.4058
7.3845
7.3643
7.1374
6.7001
6.6773
6.6471
6.6141
6.5853
3.7366
3.5983
3.5559
3.3862
3.3437
2.1861
1.6171
OMe
Me
HO
N
H
O
N
Cl
Boc
5l
1.97
1.00
3.23
3.24
1.09
1.09
3.03
8.96
f1 (ppm)

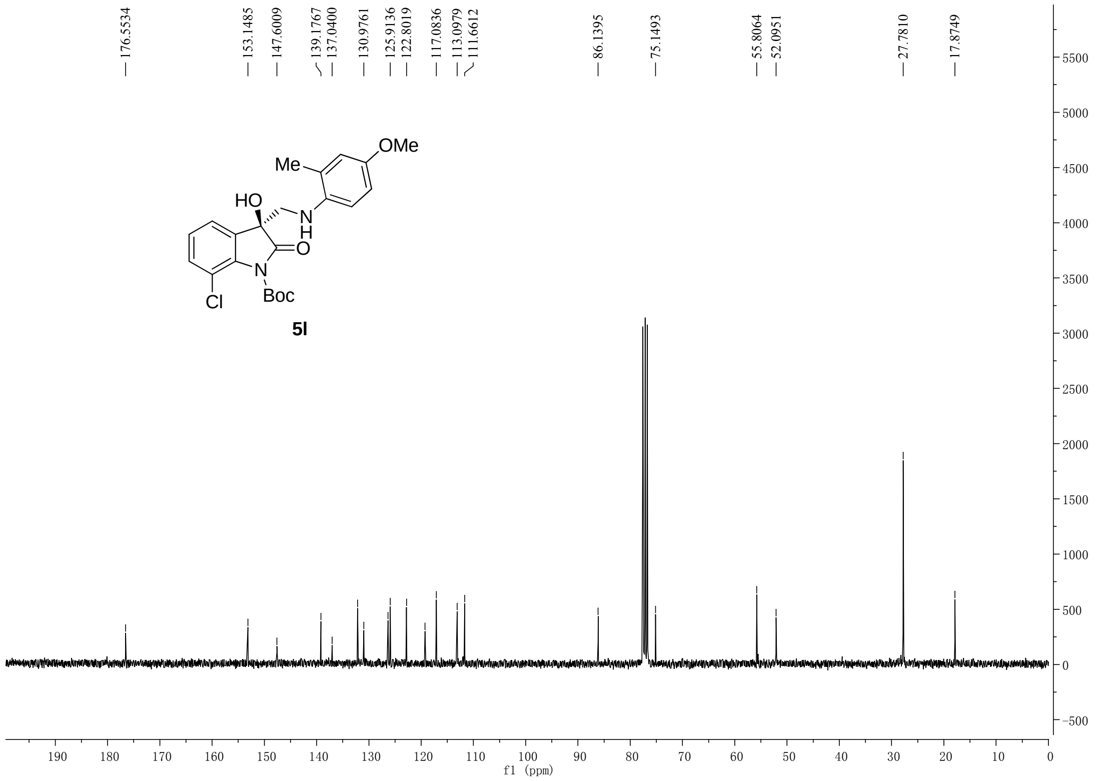
176.5534
153.1485
147.6009
139.1767
137.0400
130.9761
125.9136
122.8019
117.0836
113.0979
111.6612
86.1395
75.1493
55.8064
52.0951
27.7810
17.8749
OMe
Me
HO
N
H
O
N
Cl
Boc
5l
f1 (ppm)

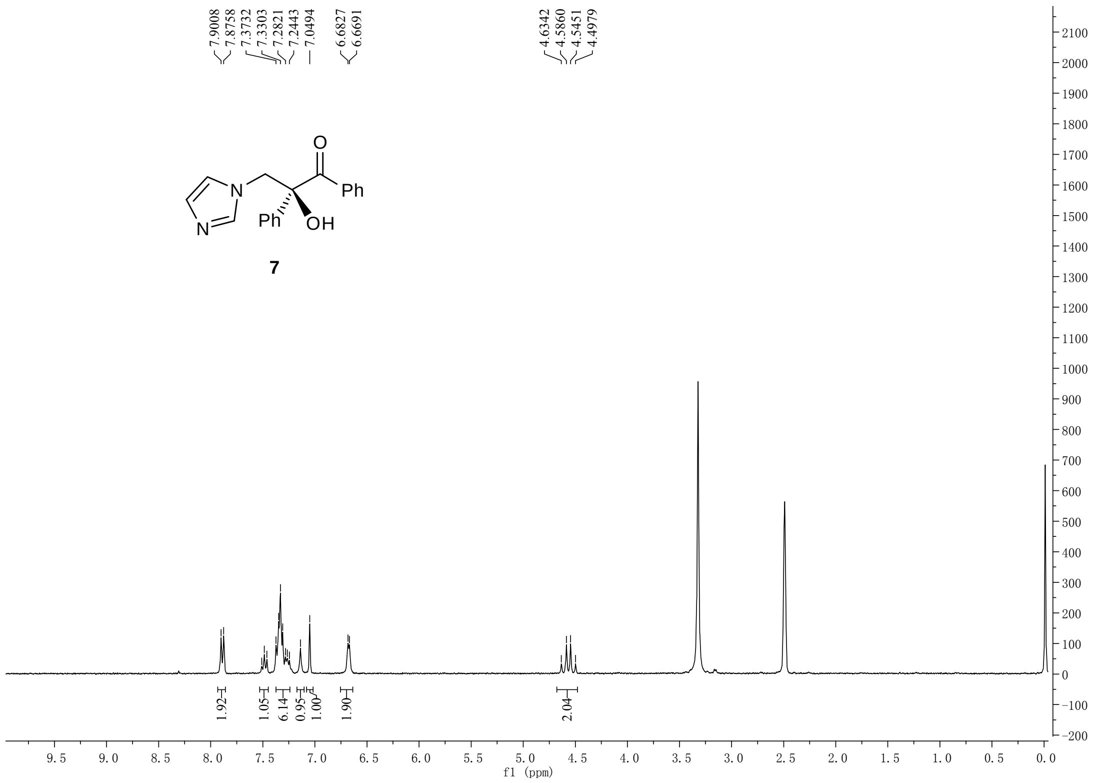
7.9008
7.8758
7.3732
7.3303
7.2821
7.2443
7.0494
6.6827
6.6691
4.6342
4.5860
4.5451
4.4979
O
Ph
N
N
Ph
OH
7
1.92
1.05
6.14
0.95
1.00
1.90
2.04
f1 (ppm)

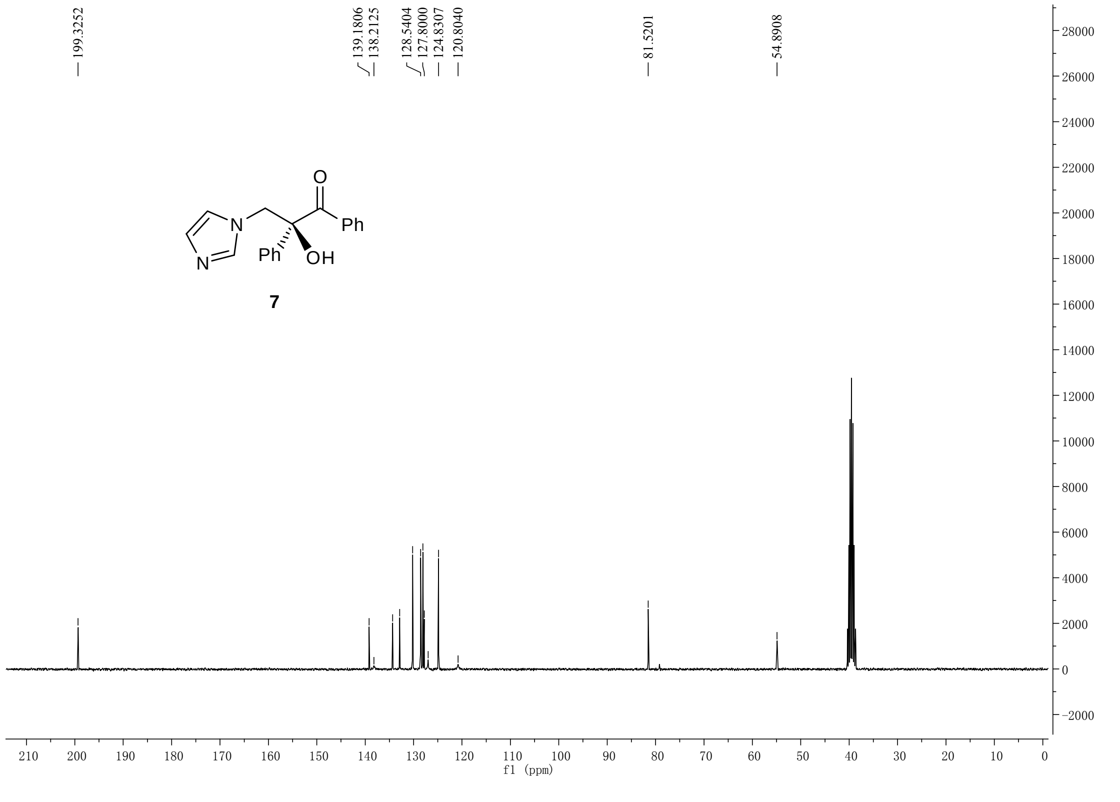
199.3252
139.1806
138.2125
128.5404
127.8000
124.8307
120.8040
81.5201
54.8908
O
Ph
N
N
Ph
OH
7
f1 (ppm)

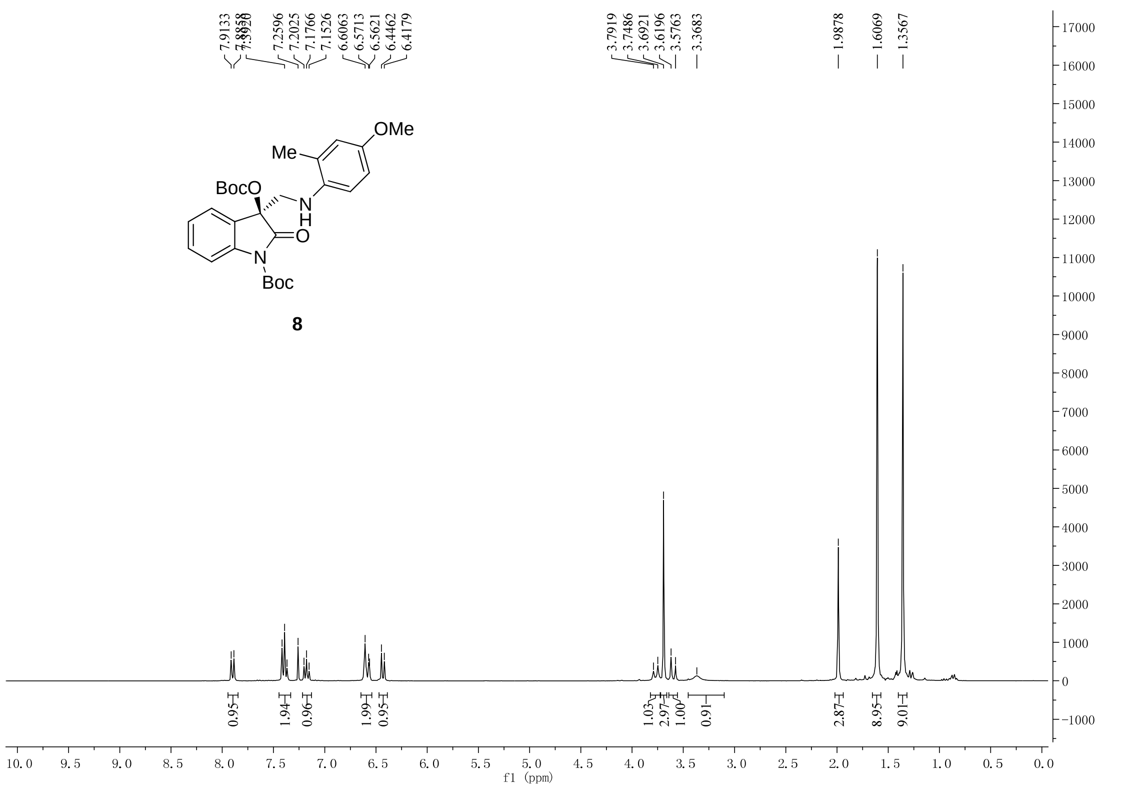
7.9133
7.2596
7.2025
7.1766
7.1526
6.6063
6.5713
6.5621
6.4462
6.4179
3.7919
3.7486
3.6921
3.6196
3.5763
3.3683
1.9878
1.6069
1.3567
OMe
Me
BocO
N
H
O
N
Boc
8
0.95
1.94
0.96
1.99
0.95
1.03
2.97
1.00
0.91
2.87
8.95
9.01
f1 (ppm)

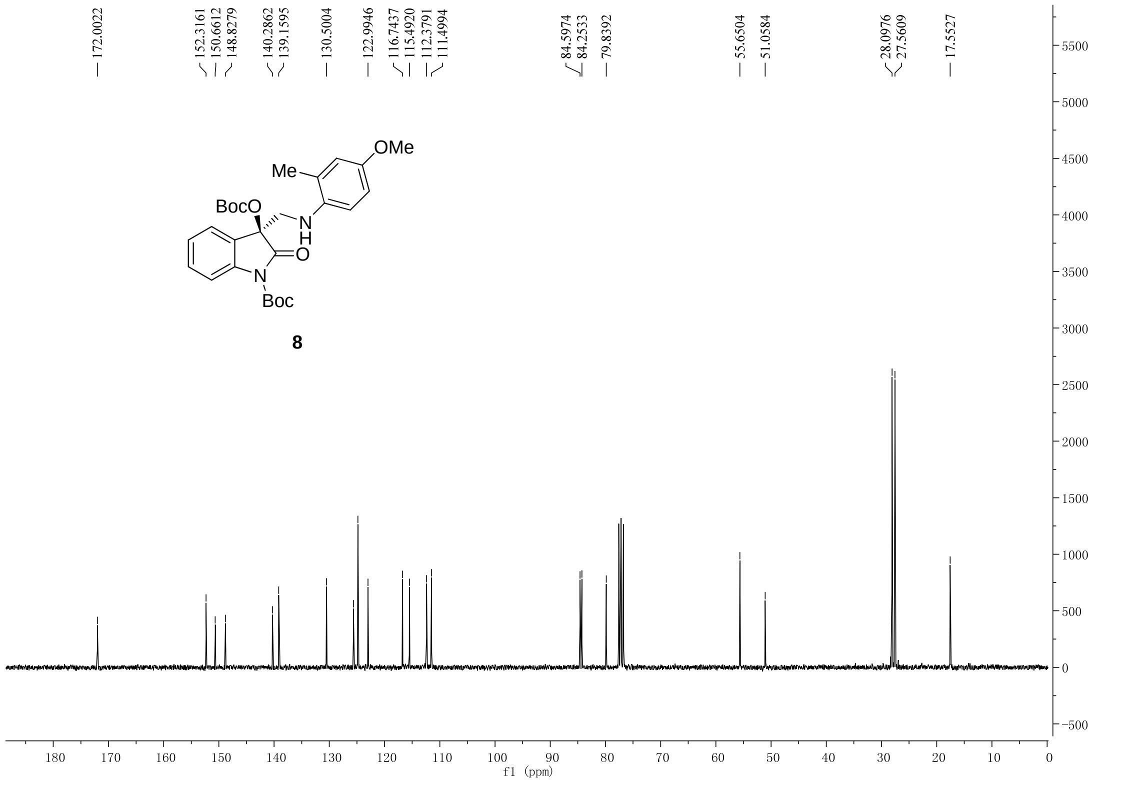
172.0022
152.3161
150.6612
148.8279
140.2862
139.1595
130.5004
122.9946
116.7437
115.4920
112.3791
111.4994
84.5974
84.2533
79.8392
55.6504
51.0584
28.0976
27.5609
17.5527
OMe
Me
BocO
N
H
O
N
Boc
8
f1 (ppm)

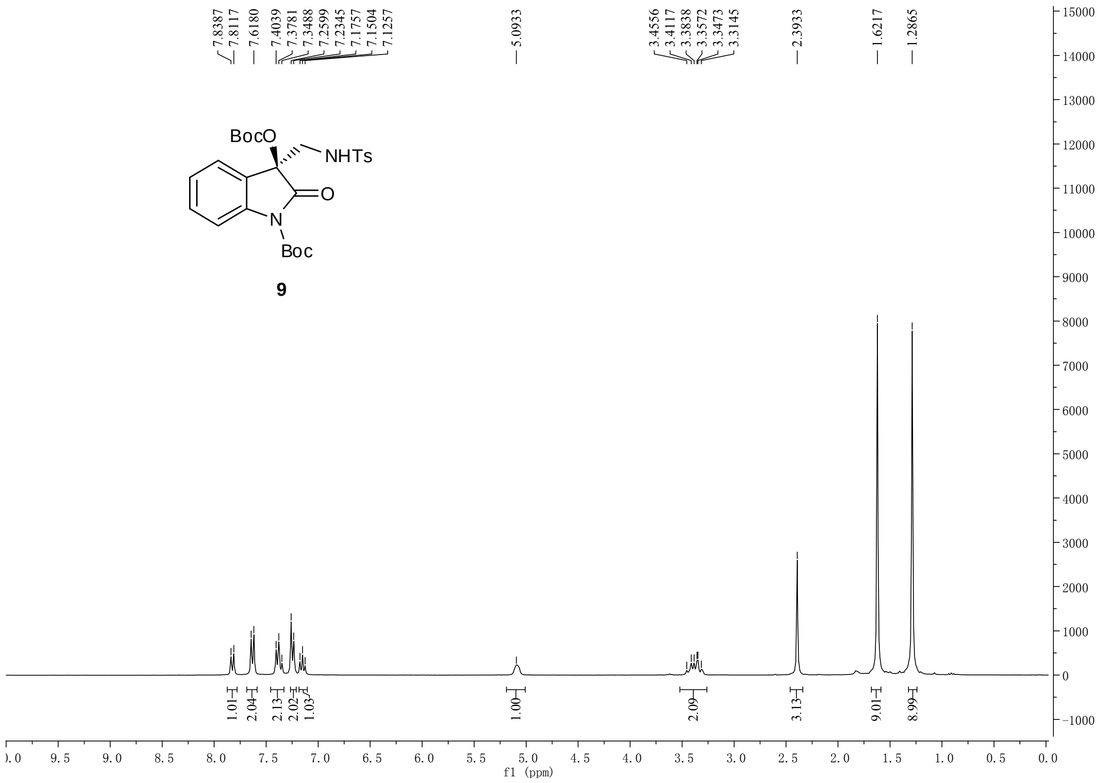
7.8387
7.8117
7.6180
7.4039
7.3781
7.3488
7.2599
7.2345
7.1757
7.1504
7.1257
5.0933
3.4556
3.4117
3.3838
3.3572
3.3473
3.3145
2.3933
1.6217
1.2865
BocO
NHTs
O
N
Boc
9
1.01
2.04
2.13
2.02
1.03
1.00
2.09
3.13
9.01
8.99
f1 (ppm)

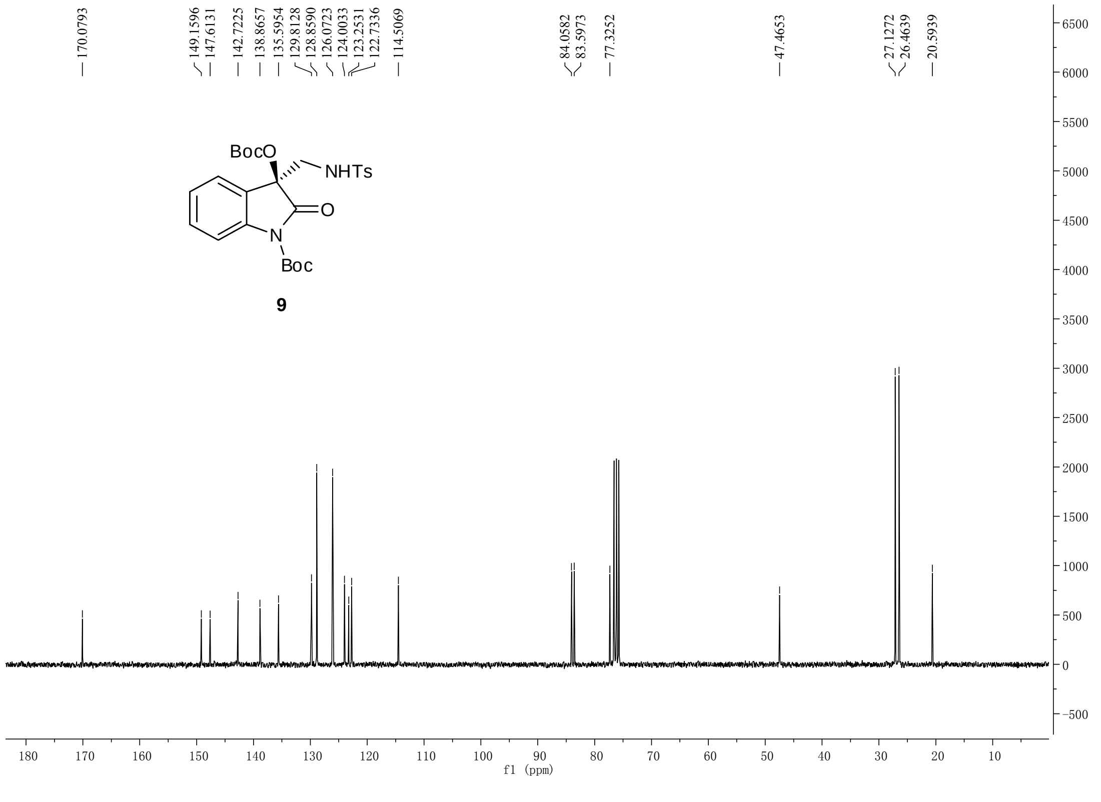
170.0793
149.1596
147.6131
142.7225
138.8657
135.5954
129.8128
128.8590
126.0723
124.0033
123.2531
122.7336
114.5069
84.0582
83.5973
77.3252
47.4653
27.1272
26.4639
20.5939
BocO
NHTs
O
N
Boc
9
f1 (ppm)

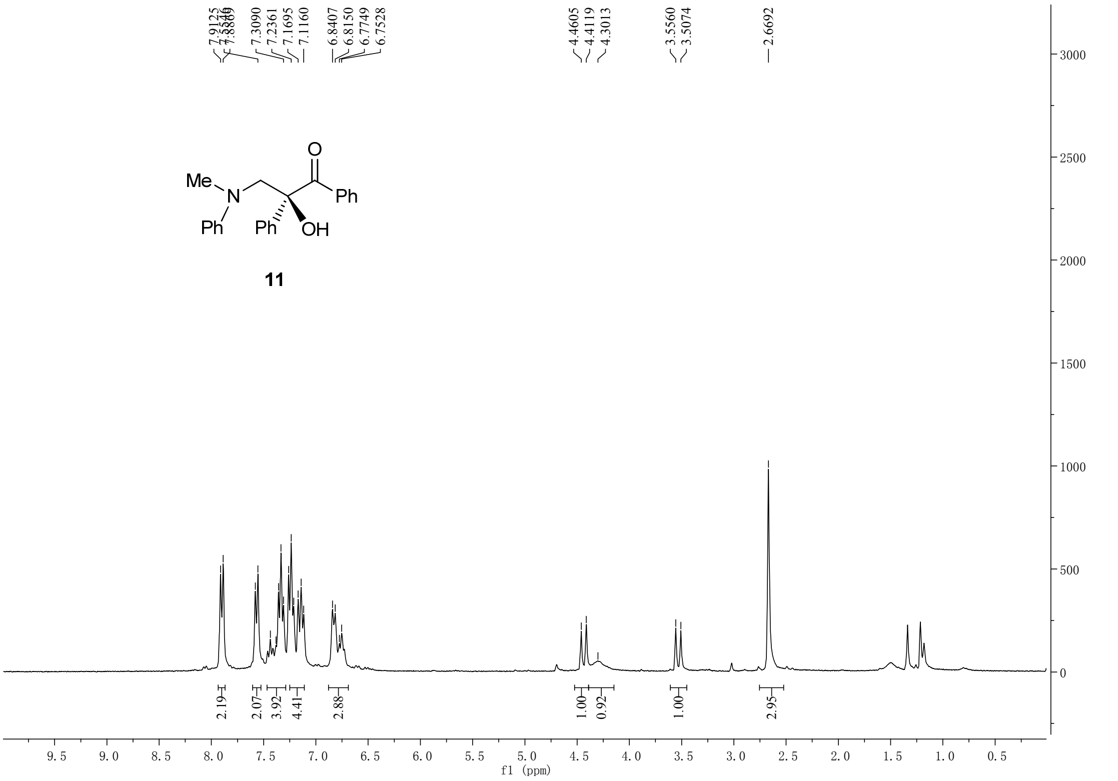
7.9125
7.3090
7.2361
7.1695
7.1160
6.8407
6.8150
6.7749
6.7528
4.4605
4.4119
4.3013
3.5560
3.5074
2.6692
Me
N
O
Ph
Ph
Ph
OH
11
2.19
2.07
3.92
4.41
2.88
1.00
0.92
1.00
2.95
f1 (ppm)

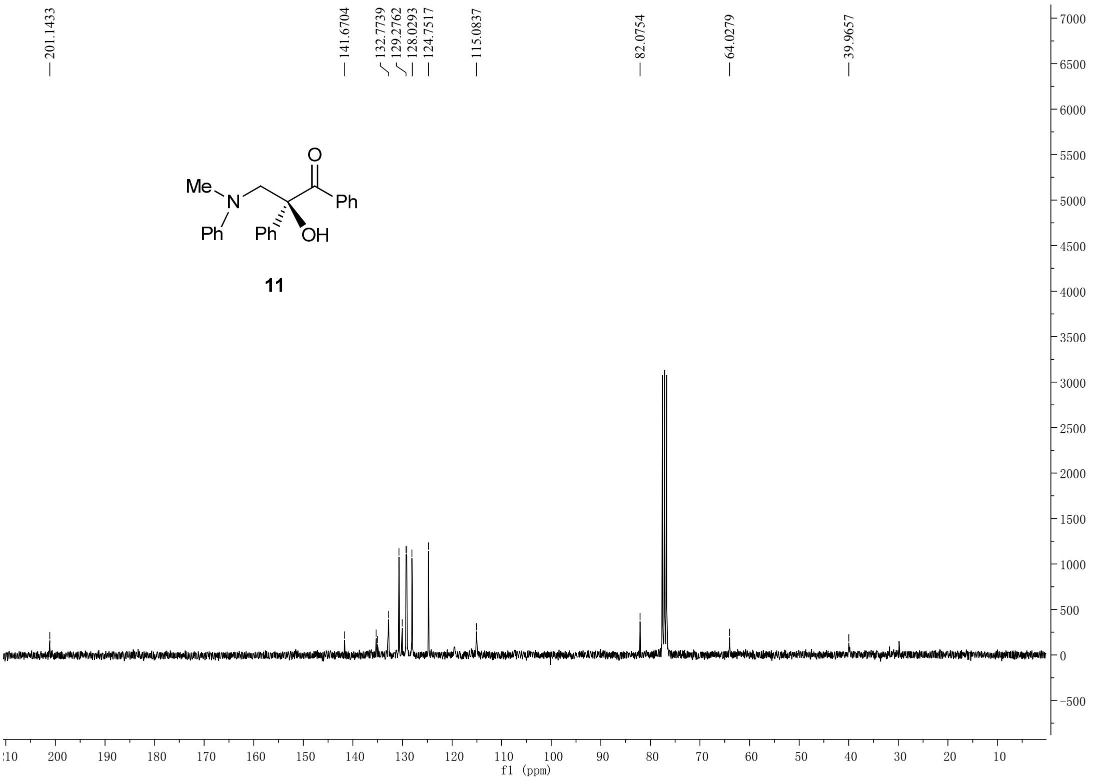
201.1433
141.6704
132.7739
129.2762
128.0293
124.7517
115.0837
82.0754
64.0279
39.9657
Me
N
O
Ph
Ph
Ph
OH
11
f1 (ppm)
